# Multi-level Modeling of Light-Induced Stomatal Opening Offers New Insights into Its Regulation by Drought

**DOI:** 10.1371/journal.pcbi.1003930

**Published:** 2014-11-13

**Authors:** Zhongyao Sun, Xiaofen Jin, Réka Albert, Sarah M. Assmann

**Affiliations:** 1 Department of Physics, The Pennsylvania State University, University Park, Pennsylvania, United States of America; 2 Department of Biology, The Pennsylvania State University, University Park, Pennsylvania, United States of America; National Center for Biotechnology Information (NCBI), United States of America

## Abstract

Plant guard cells gate CO_2_ uptake and transpirational water loss through stomatal pores. As a result of decades of experimental investigation, there is an abundance of information on the involvement of specific proteins and secondary messengers in the regulation of stomatal movements and on the pairwise relationships between guard cell components. We constructed a multi-level dynamic model of guard cell signal transduction during light-induced stomatal opening and of the effect of the plant hormone abscisic acid (ABA) on this process. The model integrates into a coherent network the direct and indirect biological evidence regarding the regulation of seventy components implicated in stomatal opening. Analysis of this signal transduction network identified robust cross-talk between blue light and ABA, in which [Ca^2+^]_c_ plays a key role, and indicated an absence of cross-talk between red light and ABA. The dynamic model captured more than 10^31^ distinct states for the system and yielded outcomes that were in qualitative agreement with a wide variety of previous experimental results. We obtained novel model predictions by simulating single component knockout phenotypes. We found that under white light or blue light, over 60%, and under red light, over 90% of all simulated knockouts had similar opening responses as wild type, showing that the system is robust against single node loss. The model revealed an open question concerning the effect of ABA on red light-induced stomatal opening. We experimentally showed that ABA is able to inhibit red light-induced stomatal opening, and our model offers possible hypotheses for the underlying mechanism, which point to potential future experiments. Our modelling methodology combines simplicity and flexibility with dynamic richness, making it well suited for a wide class of biological regulatory systems.

## Author Summary

Stomata are microscopic pores surrounded and regulated by pairs of guard cells located on the surface of plant leaves. Stomata participate in CO_2_ uptake, O_2_ release and water vapor loss. Blue and red light induce stomatal opening (enlargement of the pores), which allows the uptake of CO_2_, providing the raw material for photosynthesis, and the release of O_2_ into the atmosphere. The stress hormone abscisic acid induces minimization of pore width, decreasing water loss through transpiration. During drought conditions these counteracting stimuli jointly determine the overall stomatal movement through an integrated guard cell signalling cascade. We synthesized this interaction network between blue light, red light, and abscisic acid by aggregating and interpreting the abundant biological evidence that has been accumulated to date. We used the resulting network as the basis of a multi-level dynamic model of stomatal opening regulation in response to multiple stimuli. The model is validated by comparing its results to a large number of published experimental observations. Our model, and our experiments inspired by it, reveal an unexplored facet of the interplay between light and abscisic acid in guard cell signalling. The model directs future experiments, and its methodology can readily be applied to other systems.

## Introduction

Stomata are small pores located in the epidermes of plants that allow carbon dioxide (CO_2_) uptake for photosynthesis as well as diffusion of O_2_, produced by photosynthetic reactions, from the plant to the atmosphere. They are also the sites of water vapour loss through transpiration. Stomata are bordered by pairs of guard cells, the swelling of which leads to stomatal opening (enlargement of the pore), while their shrinking leads to stomatal closure. The size and shape change of the guard cells is due to their uptake or loss of water, which is driven by changes in cellular osmotic potential as a result of the accumulation or depletion of solutes. Guard cells are sensitive to multiple external and internal stimuli, e.g. light, intercellular CO_2_ concentration (C_i_), the stress hormone abscisic acid (ABA), and vapour pressure difference (VPD) between the leaf interior and the surrounding atmosphere [1]–[9]. Guard cells have photoreceptors for red and blue light, and guard cell responses to light of these wavelengths are the main focus of our work. As stomatal aperture regulation has a major impact on both the hydration status and the photosynthetic status of the plant, guard cells' sensitivity to stimuli is vital to the survival of vascular terrestrial plants. Plants' successful adaptation to the environment influences all life-forms on Earth. In particular, better understanding of the signalling and regulatory networks involved in stomatal responses is a necessary step toward improving the drought tolerance of crops.

Guard cells have long been a popular system for dissecting the functions of individual genes and proteins within signalling cascades. The most studied signals are blue light and ABA [10]. There has been extensive experimentation carried out to elucidate the roles of key signaling proteins, enzymes, and small molecules in these signal transduction pathways, and to identify the relationships between diverse components in the system. Numerous experiments have addressed the roles of light quality [3], [5], [6], C_i_ [11], and VPD [5], [12]. A synergistic action between blue and red light in the formation of malate, a major intracellular osmoticum, was discovered [13]. Phototropins were identified as blue light-specific photoreceptors of guard cells [14]–[16], mediating blue light-specific stomatal opening. New evidence constantly adds to our knowledge on guard cell functioning, e.g. the recently discovered inhibition by phosphatidic acid (PA) of blue light-induced stomatal opening via type 1 protein phosphatase (PP1) [17] and the relationship between the activation of the H^+^-ATPase and light quality [18]. Much less has been done, however, on a systems level to synthesize all existing evidence into a network model of light-regulated stomatal opening, or to elucidate the crosstalk between different signal transduction cascades, such as those triggered by light and ABA. One such pioneering work was done by Li *et al.* on modeling the ABA signal transduction network leading to stomatal closure [19]. That work synthesized the published evidence for direct interactions and indirect causal effects between cellular components into a consistent network of ABA-induced closure and formulated a Boolean dynamic model that recapitulated or predicted a large number of knockout phenotypes. Another recent systems level advance is the development of the OnGuard software that incorporates ion transporters at the guard cell plasma and vacuolar membrane, the salient features of osmolyte metabolism, and the major controls of cytosolic Ca^2+^ concentration and pH [20]. In this software, and models that use it [21], [22], the light signal transduction pathways are approximated by a pre-defined, light-dependent increase in the activities of all ion-translocating ATPases at the plasma and vacuolar membrane, and in sucrose and malate synthesis. That work does not consider light of different wavelengths nor the specific mechanisms through which the different types of light signals are perceived and transduced.

Given the abundance of experimental results regarding stomatal opening and its regulation, dynamic modelling of the full light-stimulated stomatal opening process and its inhibition by ABA is now tenable, and is the focus of this work. We synthesize more than 85 articles describing experimental observations into a comprehensive network of 70 components, of which 4 are signals (blue light, red light, CO_2_, and ABA), and stomatal opening is the sole output. The network incorporates in a parsimonious manner more than 150 interactions or causal relationships between components. We develop a dynamic model based on the network by characterizing each component with discrete activity levels and by describing its regulation with a combination of logic and algebraic functions. The multiple activity levels of the components and the detailed updating functions offer a biologically more accurate representation of the system than Boolean models; for example, the output node, stomatal opening, has more than 20 levels in the model, ranging from 0 to 14.2. The model has a repertoire of more than 10^31^ distinct states (see Text S1), which gives it substantial dynamic richness and makes it one of the most complex dynamic models of biological systems (see also [23]–[26]). At the same time the discreteness of the states maintains the computational simplicity of the model. The model recapitulates a comprehensive array of known behaviours and phenotypes. Since the model is made up of node-level information (i.e. the regulatory function of each component), this agreement serves as validation. The model enables an unprecedented understanding of the regulation of stomatal opening and predicts new phenotypes caused by the disruption of components. Moreover, the model reveals aspects of the system, particularly in the interplay between red light and ABA, where critical experimental evidence is lacking.

## Results

### Assembly of the light-induced stomatal opening signal transduction network

The first step in building the model is to construct the regulatory network that represents the system. A network is an abstraction of a system in which each element is represented as a node, and each pairwise interaction or regulatory relationship is represented by an edge. Edges in signal transduction networks are generally directed (meaning that the interaction has a source and a target) and signed (positive or negative). The majority of the known components involved in stomatal opening are proteins, including receptors, enzymes, channels, protein kinases and phosphatases, thus most of the nodes of the network represent proteins. To be able to incorporate the metabolic processes and ion fluxes also involved in stomatal opening, we also include important inorganic compounds, ions, certain biological processes (i.e. photophosphorylation, carbon fixation, stomatal opening) and entities (e.g. mitochondria) as nodes. In some cases, the subcellular localization of a molecule or enzyme can change, making a key difference in the modulation of stomatal opening. In these cases we use multiple nodes, one for each location. Positive edges in our network correspond to activation, up-regulation, or biochemical synthesis, and are represented with a terminating arrowhead, while negative edges indicate deactivation, inhibition, or consumption, and are shown as terminating in a solid circle. The translocation of a protein or the transport of solutes through channels or carriers is also represented by an edge. A relationship stimulated by another component of the network is represented by an edge starting from the stimulus node and incident on the stimulated edge. For instance, malate exits the cytosol and enters the apoplast through active anion efflux channels (AnionCh); this is represented by an edge from AnionCh incident on the edge that starts from cytosolic malate and ends in apoplastic malate. Certain causal regulatory relationships may be mediated by other nodes; a path (a sequence of nodes and edges) is a better representation of such indirect relationships between nodes. We used logical inference to incorporate the components suggested by the totality of relevant experiments to mediate such indirect causal relationships; this process has been formalized previously [19], [27]. We distilled more than 85 articles from the literature into 153 edges among 70 nodes, summarized in Table S1. Text S2 provides an illustration of the process of network construction based on the literature, in which the pathway that starts from blue light and ends at the H^+^-ATPase is used as an example.

The plant hormone ABA, produced in response to environmental stresses such as drought, opposes the effect of light on guard cells and reduces stomatal apertures [7], [10], [28], [29]. To maintain our focus on stomatal response to light, yet to be able to investigate the cross-talk between different signals, the ABA-response section of the model is a condensed representation of the relevant pathways. We followed two contraction principles to achieve a simpler yet dynamically equivalent representation of the system [30]. Functionally redundant pathways in this section are merged; for instance, the two mechanisms by which NO can elicit calcium release from intracellular stores (CaR) (by cyclic ADP-ribose or by 8-nitro-cyclic guanosine monophosphate [31], [32]) are compressed into a single edge from NO to CaR. Further, if the sole known function of an element is to pass on the signal it received, i.e. it has a single incoming activation edge and a single outgoing activation edge, the element is not shown in our model and its upstream regulator is directly connected to its downstream target.

**Figure 1:**
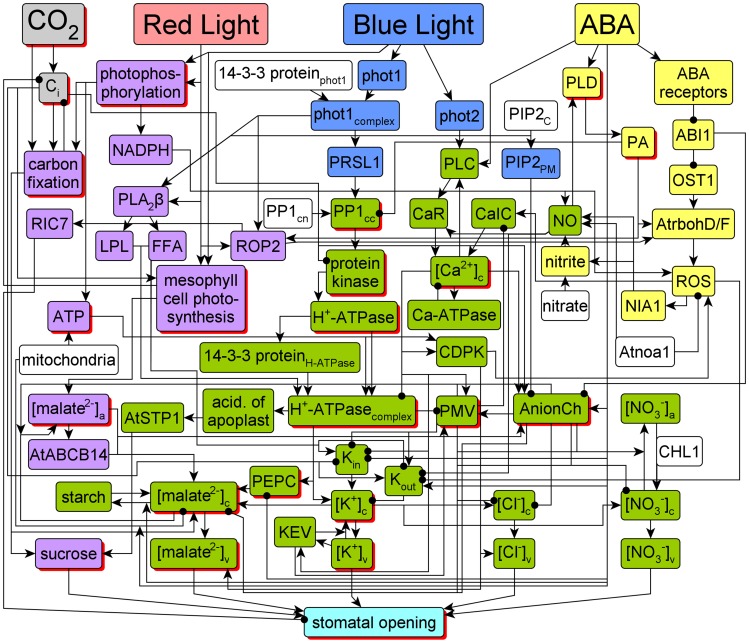
Current knowledge of light-induced stomatal opening and its regulation by CO_2_ and ABA. The color of the nodes represents their functional connectivity relative to the four signal nodes: CO_2_, red light, blue light, and ABA. CO_2_ and C_i_ are coloured grey. Nodes that can be activated by blue light alone are coloured blue. Nodes that can be activated by either red or blue light are coloured purple. Nodes are coloured yellow if they respond to the plant hormone ABA, and green if they are affected by both ABA and blue light. Nodes with no upstream effectors (called source nodes) are colored white, stomatal opening is coloured teal. We use a red shadow to indicate nodes that are characterized by three or more levelsin the dynamic model. To improve the visualization, multiple edges that originate from a single node may start together and bifurcate later toward their individual targets. Similarly, multiple positive edges that end at the same node may merge before reaching the target. Edge bifurcation or merging forms T-shaped junctions, while the crossing of two edges forms plus-shaped junctions. The full names of the network components denoted by abbreviated node names are: 14-3-3 protein_H-ATPase_, 14-3-3 protein that binds to H^+^-ATPase; 14-3-3 protein_phot1_, 14-3-3 protein that binds to phototropin 1; ABA, abscisic acid; ABI1, 2C-type protein phosphatase; acid. of apoplast, the acidification of the apoplast; AnionCh, anion efflux channels at the plasma membrane; AtABCB14, ABC transporter gene AtABCB14; Atnoa1, protein nitric oxide-associated 1; AtrbohD/F, NADPH oxidase D/F; AtSTP1, H-monosaccharide symporter gene AtSTP1; Ca-ATPase, Ca-ATPases and Ca^2+^/H^+^ antiporters responsible for Ca^2+^ efflux from the cytosol; CaIC, inward Ca^2+^ permeable channels; CaR, Ca^2+^ release from intracellular stores; carbon fixation, light-independent reactions of photosynthesis; CDPK, Ca^2+^-dependent protein kinases; CHL1, dual-affinity nitrate transporter gene AtNRT1.1; C_i_, intercellular CO_2_ concentration; FFA, free fatty acids; H^+^-ATPase, the phosphorylated H-ATPase at the plasma membrane prior to the binding of the H^+^-ATPase 14-3-3 protein; H^+^-ATPase_complex_, 14-3-3 protein bound H^+^-ATPase; KEV, K^+^ efflux from vacuole to the cytosol; K_in_, K^+^ inward channels at the plasma membrane; K_out_, K^+^ outward channels at plasma membrane; LPL, lysophospholipids; NADPH, reduced form of nicotinamide adenine dinucleotide phosphate; NIA1, nitrate reductase; NO, nitric oxide; OST1, protein kinase open stomata 1; PA, phosphatidic acid; PEPC, phospho*enol*pyruvate carboxylase; phot1, phototropin 1; phot1_complex_, 14-3-3 protein bound phototropin 1; phot2, phototropin 2; photophosphorylation, light-dependent reactions of photosynthesis; PIP2_C_, phosphatidylinositol 4,5-bisphosphate located in the cytosol; PIP2_PM_, phosphatidylinositol 4,5-bisphosphate located at the plasma membrane; PLA_2_β, phospholipase A_2_β; PLC, phospholipase C; PLD, phospholipase D; PMV, electric potential difference across the plasma membrane; PP1_cn_, the catalytic subunit of type 1 phosphatase located in the nucleus; PP1_cc_, the catalytic subunit of type 1 phosphatase located in the cytosol; protein kinase, a serine/threonine protein kinase that directly phosphorylates the plasma membrane H-ATPase; PRSL1, type 1 protein phosphatase regulatory subunit 2-like protein1; RIC7, ROP-interactive CRIB motif-containing protein 7; ROP2, small GTPase ROP2; ROS, reactive oxygen species; [Ca^2+^]_c_, cytosolic Ca^2+^ concentration; [Cl^-^]_c/v_, cytosolic/vacuolar Cl^-^ concentration; [K^+^]_c/v_, cytosolic/vacuolar K^+^ concentration; [malate^2-^]_a/c/v_, apoplastic/cytosolic/vacuolar malate^2-^ concentration; [NO_3_^-^]_a/c/v_, apoplastic/cytosolic/vacuolar nitrate concentration.

Figure 1 represents the resulting network of 70 nodes and 153 edges. The colour coding of the nodes signifies the functional connectivity of each node to the four signals, which is based on the existence of paths between a signal and the respective node but is also informed by the specific combinatorial regulation of the node (described in detail in the section “Elements of the dynamic model”). A brief description of the biology represented by the network is as follows; Text S3 provides a detailed description of the network. Both red and blue light activate guard cell photophosphorylation, providing adenosine triphosphate (ATP), the primary chemical energy transporter within the cell, for metabolic processes [33]. Subsequent carbon fixation provides sugars, primarily sucrose, as osmotica for guard cell swelling and stomatal opening [34], [35]. This pathway is formed by purple coloured nodes in the left side of the network. A blue light-specific pathway (blue coloured symbols) leads to the activation of the plasma membrane H^+^-ATPase [4], [36]. H^+^-ATPase activity hyperpolarizes the plasma membrane [4], with subsequent uptake of K^+^ [37], [38] and accumulation of its counterions, Cl^-^, NO_3_^-^, and malate^2-^ [13], [33]. These ions also function as osmotica during light-induced stomatal opening [6], [39], [40]. The stress hormone ABA initiates a signal transduction network (yellow nodes) which ultimately inhibits the plasma membrane H^+^-ATPase, inhibits malate synthesis, and induces malate breakdown and release [2], [41]–[45]. Thus the majority of the nodes in the network (the green-coloured nodes) are regulated by blue light and ABA. The twenty-three nodes that have more than two levels in our model are highlighted with a red shadow.

### Structural analysis of the network

Representing a system with a network reveals important characteristics and interrelationships that have been hidden previously, and enables researchers to test prevailing theories and to identify new hypotheses [46]. We started by looking at the node degree, defined as the number of edges to which the node is connected, of the 70 nodes. The degree can be broken into the in-degree, i.e. the number of incoming edges (and therefore, of direct upstream regulators), and the out-degree, i.e. the number of outgoing edges (and therefore, of direct downstream targets). The four signal nodes, blue light, red light, CO_2_, and ABA, have an in-degree of zero. The node stomatal opening is the only node in the system with an out-degree of zero. Table 1 lists the 10% of nodes with the highest in-degree, out-degree, and total degree, respectively. Most nodes in this list are known key mediators or regulators of light-induced opening. For instance, the node that represents the cytosolic malate^2-^ concentration has the highest in-degree and also the highest total degree in the network. Malate, the major counterion for K^+^ in guard cells and a common organic metabolite, is indeed involved in multiple metabolic pathways. The node that represents the 14-3-3 protein-bound H^+^ ATPase, H^+^-ATPase_complex,_ is also among the nodes with highest in-degree and total degree, indicating its multi-tiered regulation and its important role in determining the membrane potential and hence the flow of multiple ions. The ion channels K_in_, K_out_, and anion efflux channels are also among the highly regulated nodes in the system. The stress hormone ABA has the highest out-degree, due to its targeting of multiple nodes in the pathway of blue light-induced stomatal opening and in the ABA signalling network. Cytosolic Ca^2+^ concentration ([Ca^2+^]_c_) is an important secondary messenger for both blue light and ABA signalling, as reflected by its high out-degree and total degree. The node PMV, which denotes the potential across the plasma membrane, also has high out-degree and total degree, reflecting its control of channel activities.

**Table 1:** The top 10% of nodes in terms of in-degree, out-degree, and total degree in the network.

Degree Types and Values		List of Nodes
In-degree	10	[malate^2-^]_c_
	6	H^+^-ATPase_complex_, K_out_, AnionCh, stomatal opening
	5	K_in_, [K^+^]_c_, [NO_3_^-^]_c_
Out-degree	9	ABA
	8	[Ca^2+^]_c_, PMV
	6	AnionCh, C_i_
	5	phot2
Degree	13	[malate^2-^]_c_
	12	PMV, AnionCh
	11	[Ca^2+^]_c_
	10	H^+^-ATPase_complex_
	9	[K^+^]_c_, ABA

For example, the node AnionCh has an in-degree of 6, an out degree of 6, and a total degree of 12.

**Figure 2:**
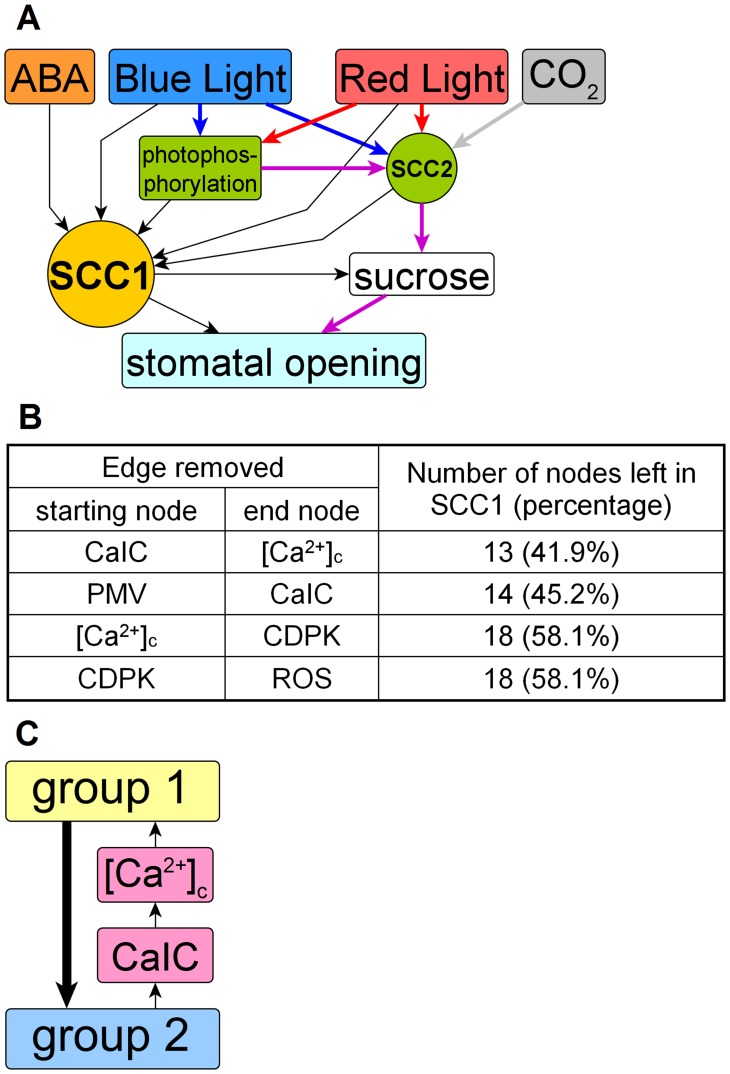
Structural analysis of the signalling network. (A) Compressed representation of the network that shows the four signals (input nodes) of the network, two composite nodes that represent SCC1 (which contains 31 nodes) and SCC2 (which contains 3 nodes), as well as photophosphorylation, sucrose, and the output node of the network, stomatal opening. The nodes not shown do not affect the network's connectivity and are contracted into the edges shown in black. Five paths do not cross SCC1; they start from blue light, red light, or CO_2_, pass through photophosphorylation, merge at SCC2, reach sucrose, and lead to stomatal opening. Signal-specific edges are coloured blue (for blue light), red (for red light), grey (for CO_2_); the edges shared by both blue and red light are purple. (B) The 4 edges whose removal results in the highest node loss from SCC1. The unperturbed SCC1 contains 31 nodes, which is the basis for the percentage calculation. (C) Sub-structure of SCC1. Group 1 contains 12 nodes, and group 2 contains 17 nodes.

Next, we identified the strongly connected components of the system. A strongly connected component is a group of nodes wherein any node is reachable from any other node through a path (a series of consecutive nodes and edges). Intuitively, a strongly connected component is a closely-knit group of nodes with interwoven feedback that usually forms an important functional module of a network. The stomatal opening network contains three strongly connected components (SCCs), comprising 31 nodes (SCC1), 3 nodes (SCC2) and 2 nodes (SCC3), respectively (Figure 2A). The 3-node SCC2 represents the interplay amongst C_i_ and carbon fixation processes in guard cells and mesophyll cells: C_i_ is required by photosynthesis and photosynthesis lowers C_i_ in turn. The 2-node SCC3 represents the two directions of transport between apoplastic and cytosolic NO_3_^-^. The largest SCC signifies the crosstalk between the different signals of the system, since all four signals of our model connect to it. Eight of the thirteen high-degree nodes listed in Table 1 are in the largest SCC. Most of the remaining high degree nodes have only outgoing or incoming edges and thus cannot be strongly connected. Twenty-seven nodes, including the nodes of SCC2, can reach the nodes of SCC1 through directed paths. Eleven nodes, including SCC3, can be reached from SCC1 through directed paths. Only a single node, CHL1, is not connected to SCC1 by a directed path.

There are 19,436 simple paths (i.e., paths with no repeated nodes) between the four signal nodes and stomatal opening. The vast majority of these paths pass through SCC1. Remarkably, five important paths bypass SCC1 (see Figure 2A). The five paths start from blue light, red light and CO_2_, respectively, pass through photophosphorylation and SCC2, and then through sucrose, and finish at stomatal opening. Photophosphorylation and SCC2 together represent the photosynthetic carbon fixation processes in guard cells and mesophyll cells. Two conclusions can be drawn based on the existence of these SCC1-bypassing paths: i) The five paths represent photosynthetic carbon reduction pathways. ABA does not inhibit photosynthesis in mesophyll cells [47], and there is no indication that ABA would inhibit guard cell photosynthesis. Thus there is no current wet bench evidence that ABA would be able to affect the accumulation of sucrose via guard cell photosynthesis (see Figure 2A). ii) Based on current knowledge, sucrose accumulation does not introduce any feedback into the system.

We next ranked the 60 edges of SCC1 according to their importance to SCC1 integrity. If a strongly connected component is densely connected, the loss of a single edge should not affect the reachability of node pairs in it. However, if after the loss of an edge certain nodes can no longer reach other nodes, or cannot be reached from other nodes, they are no longer part of the strongly connected component and thus the number of nodes in the strongly connected component decreases. Table S2 provides information on the effects of removal of each edge. Removal of any one of 26 edges led to no change in the composition of SCC1. Loss of any one of 19 edges led to minimal changes, i.e. the loss of a single node from SCC1. Among the edge removals that do induce significant breakdown, the four listed in Figure 2B lead to loss of more than 40% of the nodes in SCC1. All four edges are closely related to [Ca^2+^]_c_, indicating the critical role of [Ca^2+^]_c_ in the formation of SCC1. A closer examination revealed that SCC1 contains two smaller groups of strongly connected nodes (Figure 2C). Group 1 contains 12 nodes from the ABA signalling pathways. Group 2 contains 17 nodes, including the H^+^-ATPase, its four regulators, nodes denoting major ions, and PMV, which are major mediators of blue light signalling. Seven edges connect group 1 to group 2, which is the reason why the nodes in group 2 are coloured green in Figure 1. However, there is a single path from group 2 back to group 1, mediated by CaIC and [Ca^2+^]_c_. The loss of any of the nodes or edges involved in this path results in a major breakdown of SCC1 (Figure 2B). The fact that both groups are strongly connected with [Ca^2+^]_c_ indicates that [Ca^2+^]_c_ bridges the signalling between blue light and ABA. This conclusion is corroborated experimentally [48]–[53]. Indeed, it is known that [Ca^2+^]_c_ is an important secondary messenger in both blue light [53], [54] and ABA signalling [55], [56]. Our strongly connected component analysis offers additional insight into the role of [Ca^2+^]_c_ and reveals that it is a key participant in a feedback loop formed by these pathways.

### Elements of the dynamic model

The signal transduction network described in Figure 1 forms the basis of our dynamic model of light-induced stomatal opening. The dynamic model characterizes each node with a state variable (which we will also refer to as “level”) and with a regulatory function (also called update function) that indicates the future state of the node as a function of the current state of its regulators. Iterative determination of each node's state from a suitable initial condition yields the dynamic behaviour of the whole system. Importantly, the global dynamics of the whole system is an emergent property that is not predetermined by the modeller but arises from the local dynamics (the regulation of each component).

We developed a discrete dynamic model in which the nodes are assigned two or more qualitative levels. We aimed to employ the minimal number of levels that was sufficient to describe the experimentally observed relative outcomes for various conditions (e.g. combinations of signals and manipulations of node states). The two possible levels of binary nodes (0 and 1) can be interpreted as “OFF”, “low” or “inactive”, versus “ON”, “high”, or “active”. Three levels can be interpreted as “low”, “medium” and “high”. The benefit of having three-level nodes is most evident when three qualitatively different categories of values are observed under three or more different experimental conditions, e.g. stomatal opening under red light alone, blue light alone or under dual beam. For such scenarios, having nodes with only two levels would force the grouping of qualitatively different values, and therefore lead to information loss. Among the observations that necessitated the use of more than two levels are the synergistic (stronger than additive) effect between red light and blue light in malate formation [13] and stomatal opening [3], [5], [9], [39], [57]–[59], and the complex behaviour of [Ca^2+^]_c_ as a secondary messenger during blue light-induced stomatal opening [50], [51], [60] and ABA-induced stomatal closure [43], [48], [49], [61]. In addition, the osmotic potential difference across the plasma membrane that leads to stomatal movement results from the totality of all solutes, whose effect is biophysically additive. In our model, 47 nodes have two levels, nine nodes have three levels (including photophosphorylation, carbon fixation, [Ca^2+^]_c,_ CO_2_), two nodes have four levels (ATP, C_i_), three nodes have five levels (Protein Kinase, H^+^-ATPase_complex_, PMV) and nine nodes have more than five levels (including protein kinase, H^+^-ATPase_complex_, [K^+^]_c_, [malate^2-^]_c_, stomatal opening). Stomatal opening, in particular, has more than 20 reachable levels, ranging from 0 to 14.2. The numerical values of these levels are not meaningful in isolation; rather, their relationships are reflective of the experimentally observed relative outcomes. Text S1 provides a listing of all node levels.

The four signals of the model were assigned a set of levels that represent a particular experimental condition (light condition, CO_2_ concentration and ABA presence or absence). The possible levels of blue light, red light and ABA are ON (1) or OFF (0), indicating their presence or absence. CO_2_ has three levels, 0 (reduced CO_2_), 1 (ambient atmospheric CO_2_), and 2 (high CO_2_). The signal levels can be externally changed, e.g. to simulate a light pulse experiment. The 64 internal nodes were chosen to have an initial state of 1 (7 nodes) or 0 (57 nodes) based on experimental information. Text S1 describes these initial states and their justification.

Time is discretized into steps in our model. In one time step, the state of each node is updated according to the update function assigned to it [19]. We followed random order asynchronous update [62]. A random permutation of the nodes (except the node stomatal opening) is first established at the beginning of each time step, and then all nodes are updated according to this sequence. Stomatal opening, as the sole output of our model, is always updated last within each time step. This algorithm effectively implements a random sampling of process durations. We have chosen this random sampling due to the scarcity of experimental data on relative reaction speeds of signalling pathways and on the timing of specific intracellular events. The degree of randomness can be reduced as timing information becomes available. A delay of 10 time steps is implemented for the node sucrose (see the update function for the node sucrose in Text S1). We determined empirically that for our network a total of 18 time steps in each simulation is sufficient for all components to reach a time-invariant state (steady state or, for a minority of nodes such as [Ca^2+^]_c_, sustained oscillation).

The update function of a target node indicates the future state of the target node as a function of the current states of the nodes that have a directed edge impinging on the target. The update functions were developed with information from the literature, such as the state of the target node when one of its regulators is knocked out, and basic biochemical or physical principles when applicable. We aimed to construct the simplest update functions to minimize the number of unknown parameters in the model. The update functions combine logic clauses (using the Boolean operators NOT, AND, OR) with addition, subtraction, and multiplication. This approach enables a more detailed and accurate representation than traditional Boolean models, while maintaining simplicity and using few parameters. Two examples of update functions are given in the Materials and Methods section, and Text S1 provides a full list of the update functions and their justifications.

We tested different numbers of replicate simulations, and found that 2,000 replicate simulations were sufficient for a high reproducibility of our results (see Text S4). We also demonstrated that our model is robust against uncertainty in the update rules without losing its sensitivity to new information on critical nodes (see Text S4). For each experimental condition studied, a total of 2,000 simulations were performed, and for each node, the activity level averaged over all simulations is reported. Experimental condition refers to the level of the four signals and/or any other elements of the system that might be silenced to represent knockout (KO) experiments or made constitutively active; these factors are then invariant across all 2,000 runs.

Importantly, since the input to our model is local (the relationships among pairs of nodes, see Table S1), an agreement between the global dynamic results of simulations from the model and wet bench results is not an inherent property of the model. As shown below, however, the model does in fact successfully reproduce known dynamic features exhibited by stomatal opening under various conditions, providing strong support for the validity of the model.

### The model recapitulates and elucidates wild type responses to light

**Figure 3:**
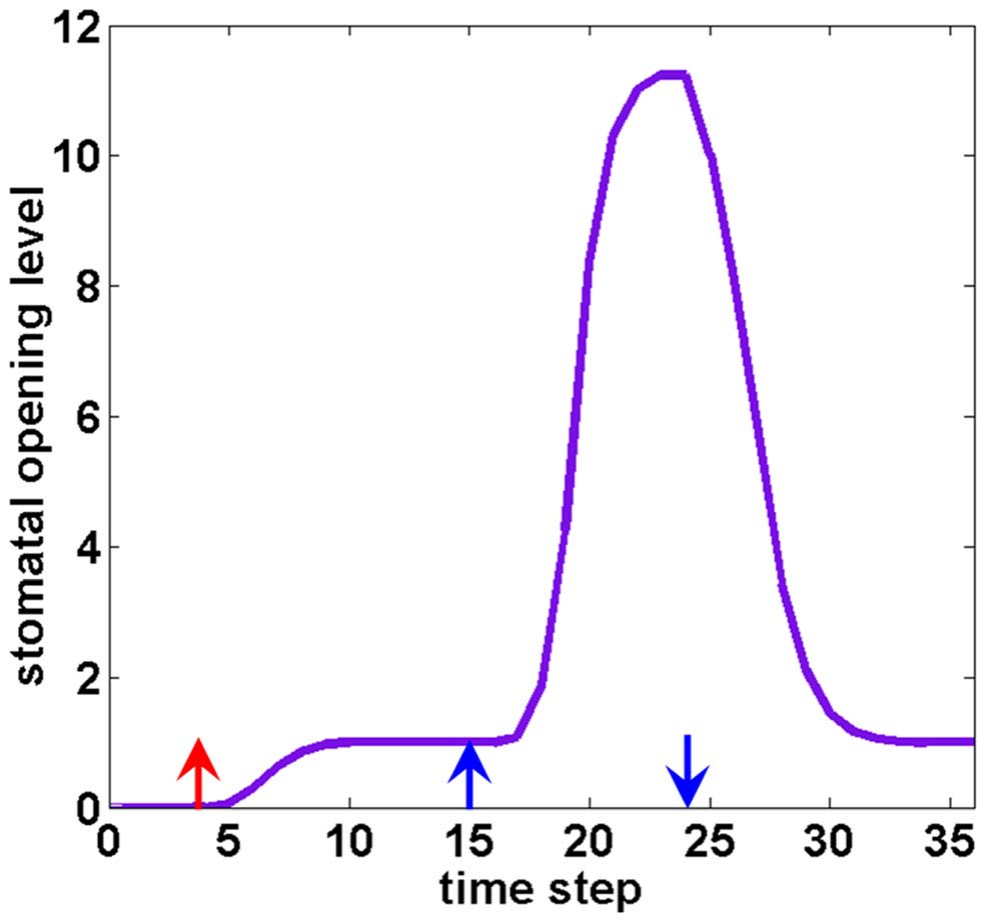
Simulation of stomatal opening level in response to a sequence of light conditions. The arrows with corresponding colours and directions signify the imposition (upward) or removal (downward) of a specific light signal. The system is in darkness at time step 0; a red light signal is added at step 4; a blue light signal is turned on at step 15 and off at step 24. The blue light signal induces a sharp increase in the stomatal opening level. The stomatal opening level gradually returns to the red light-induced steady state level after the blue light pulse.

We started by comparing the model's results to experiments under different qualities of light in ambient air. In signature experiments that investigated the roles of red and blue light in stimulating stomatal opening, leaves were illuminated with constant background red light upon which a short blue light pulse was superimposed. The stomatal conductance increased slightly in response to the red light, then displayed dramatic transient increase in response to the blue light pulse [5], [9], [57], [58]. As depicted in Figure 3, our model successfully reproduces this temporal pattern of stomatal opening.

**Figure 4:**
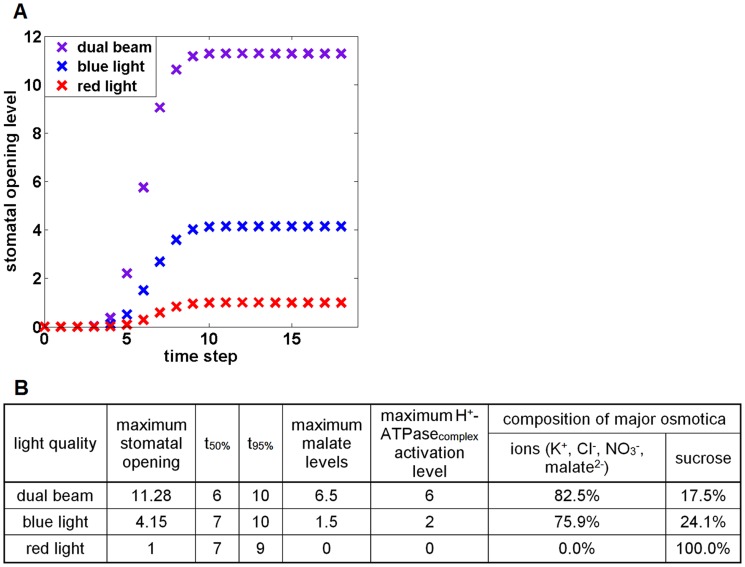
Simulation of stomatal opening under different conditions of light quality in ambient air. (A) Mean stomatal opening levels as a function of time step from 2,000 simulations. Purple: dual beam (blue light = red light = 1, CO_2_ = 1); blue: blue light (blue light = 1, red light = 0, CO_2_ = 1); red: red light (blue light = 0, red light = 1, CO_2_ = 1). The standard error of the mean for the stomatal opening level is smaller than the symbols, and is consequently not shown. (B) Summary table of results for several simulated variables. The first three columns summarize the results shown in (A) indicating the maximum (steady-state) opening level, the number of time steps at which 50% of simulations reach 50% of the maximum level (t_50%_) and the number of time steps at which 95% of simulations reach 95% of the maximum level (t_95%_). The next two columns indicate the maximum malate levels and the maximum activation levels of the H^+^-ATPase_complex_. The two right-most columns present the contribution of different osmotica (ions vs. sucrose) to stomatal opening in response to different light qualities.

We simulated wild type stomatal responses to sustained light in ambient air, as illustrated in Figure 4A. The specific combination of signals for each curve (blue light, red light, or dual beam) is initiated at time step 0 and maintained throughout the simulation. All three time courses of average stomatal opening levels (over the 2000 simulations) have similar sigmoidal shapes. We consistently observed sigmoidal timecourses for stomatal opening and other variables and in the following summarize them by three parameters (Figure 4B): the maximal (steady state) value of the mean level, the number of time steps at which 50% of simulations reach 50% of the maximal (steady state) level (t_50%_), and the number of time steps at which 95% of all simulations reach 95% of the maximal level (t_95%_). In the presence of both blue and red light, the average stomatal opening level reaches a maximum of ∼11.28 in ∼10 steps, whereas red light alone only generates an opening level of 1.00. Notably, blue light, with an opening level of 4.15, is more effective than red light in inducing opening, which is consistent with experimental observations of stomatal apertures [3], [5]. A synergistic action of red and blue light on stomatal opening, which has been observed experimentally [5], [9], [39], [57], is reproduced in Figure 4A: the stomatal opening level under both blue and red light (dual beam) is larger than the sum of opening levels under each type of light alone.

Malate, a common organic compound found in plants, is one of the major counterions for K^+^, causing guard cell swelling and stomatal opening. The action spectrum of malate formation shows a synergistic action between red and blue light, i.e. the malate synthesis level under blue light with a red light background (dual beam) is higher than the sum of levels under each type of light (red or blue) alone [13]. This provides a valuable criterion to evaluate our model. Simulation results presented in Figure 4B clearly indicate that the maximal malate level under dual beam illumination is higher than the sum of maximal levels accumulated under individual light qualities. The result that malate has no observable accumulation under red light alone is also in accordance with experiments [35].

Also listed in Figure 4B is the maximum activation level of the H^+^-ATPase_complex_ obtained in our model under each light condition with ambient air. The proton pump, H^+^-ATPase, is responsible for the plasma membrane polarization status and for concurrent ion flows. Our model indicates that the H^+^-ATPase_complex_ is activated to the highest degree under a dual beam, to a significant degree under blue light alone, and is inactive under red light alone. Experimental evidence on the activation of the H^+^-ATPase under red light alone is mixed (see Discussion). Our model supports the conclusion that in ambient air the H^+^-ATPase is not significantly activated under red light. Our result that blue light alone can activate the proton pump is consistent with multiple experiments [4], [36], [63]–[65]. Our model predicts synergy between red and blue light in the activation of the proton pump, and it suggests that this synergy is one of the mechanisms that underlies the synergy between red and blue light in stomatal opening and malate accumulation.

Figure 4B also presents our model's prediction of the relative contribution of the two major types of osmotica, ions (K^+^ and its counterions) and sucrose, to the osmotic potential under different light qualities in ambient air. These relative contributions are normalized such that their sum is 100%; see Text S1 for the detailed definition of the contribution of each osmoticum to osmotic potential in our model. The model indicates that ions are the predominant osmoticum being accumulated in response to dual beam or blue light (82.5% and 75.9%, respectively), whereas sucrose is the sole osmoticum responsible for red light-induced stomatal opening. These results agree with experimental findings: ion accumulation was observed to take place predominantly under white light or blue light, and is nearly non-observable under red light under ambient CO_2_ conditions [6], [35], [39]; sucrose accumulation takes place under either blue or red light [35].

### The model explains the effect of external CO_2_ levels

**Figure 5:**
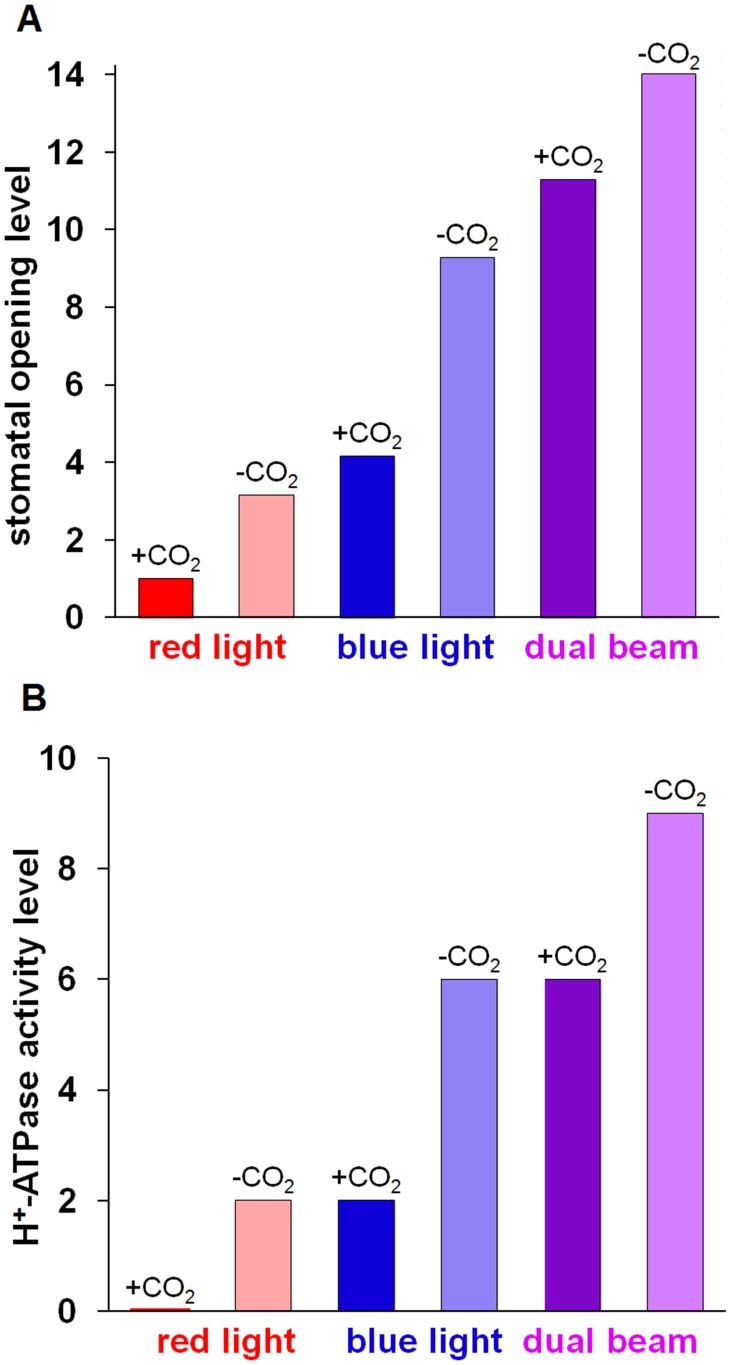
The effect of CO_2_-free air on light-induced stomatal opening and H^+^-ATPase activity. Simulations of (A) maximum stomatal opening level, and (B) maximum H^+^-ATPase activity level in air with moderate CO_2_ concentration (+CO_2_) compared to CO_2_-free air (-CO_2_) under different light conditions. Red colour indicates red light, blue colour indicates blue light, purple colour indicates dual beam. Darker colours represent air with moderate CO_2_, and lighter colours represent CO_2_-free air. (A) Stomatal opening is significantly enhanced by CO_2_-free air under all light conditions. (B) The H^+^-ATPase activity pattern parallels that of stomatal opening levels in having higher levels in the absence of CO_2_.

We investigated the effect that different levels of CO_2_, another input signal to our model, has on stomatal opening induced by different qualities of light. The CO_2_ content in the ambient atmosphere affects light-induced stomatal opening. Air with lower CO_2_ concentration or CO_2_-free air was shown to promote white light-induced stomatal opening [1], blue light-induced stomatal opening [5], [58], and red light-induced stomatal opening [40]. Our model captures the enhancement of stomatal opening levels by low CO_2_ under all light conditions (Figure 5A). Our simulations also indicate that the pattern of the maximal H^+^-ATPase activity in response to different light and CO_2_ conditions parallels that of stomatal opening (Figure 5B). Our model thus predicts that the H^+^-ATPase activity level is promoted by CO_2_-free air compared to ambient air under all light conditions, and suggests that the promotion of H^+^-ATPase activity level may contribute to the enhancement of stomatal opening levels by CO_2_-free air.

### The model recapitulates perturbation scenarios

In order to further test the validity of the model, we next investigated a number of perturbation scenarios.

DCMU, a photosynthetic inhibitor, completely inhibits red light-induced stomatal opening, but only partially inhibits blue light-induced stomatal opening [3]. Our simulation of the DCMU effect (via maintaining the node photophosphorylation at level 0) is consistent with these experimental observations: the stomatal opening level drops from 1 to 0 under red light, indicating a total inhibition, while the same disruption has a partial effect on stomatal opening induced by a dual beam or by blue light (see Table 2).

**Table 2:** Simulated effects of DCMU and fusicoccin.

A			
Treatment	Maximum Stomatal Opening	t_50%_	t_95%_
Dual Beam	11.28	6	10
Dual Beam+DCMU	3.15	6	9
Blue Light	4.15	7	10
Blue Light+DCMU	1.58	7	10
Red Light	1	7	9
Red Light+DCMU	0	0	0

B				
Treatment	Maximum Levels		t_50%_	t_95%_
Dark	Stomatal Opening	0	0	0
	K^+^ Uptake	0	0	0
Fusicoccin+Dark	Stomatal Opening	9	3	4
	K^+^ Uptake	9	3	4

(A) The effect of DCMU on stomatal opening under different light conditions. (B) Stomatal opening and K^+^ uptake induced by fusicoccin in darkness.

Fusicoccin is a fungal toxin that stimulates K^+^ uptake in guard cells and causes stomatal opening in darkness [66], [67]. Fusicoccin has been widely used as a physiological tool to investigate guard cell signaling [68]. Fusicoccin activates the plasma membrane H^+^-ATPase via a mechanism that involves inactivation of an autoinhibitory domain [69], [70]. We simulated the effect of fusicoccin on the H^+^-ATPase by fixing the state of the H^+^-ATPase at its maximum activation level, nine. Our simulation indicated that without fusicoccin, stomatal opening and K^+^ levels remain 0 in darkness (see Table 2). This is due to the absence of H^+^-ATPase activation in the dark. When fusicoccin is present, stomata open despite the absence of light, and K^+^ increases in the darkness as well. Our simulation suggests that the H^+^-ATPase, when activated by fusicoccin in the dark, leads to the hyperpolarization of the plasma membrane and the subsequent activation of K^+^ uptake channels. The accumulation of K^+^ and its counterions in the guard cell is the cause for stomatal opening in the dark in the presence of fusicoccin. Both behaviours are consistent with experimental findings [66], [67].

### The model reveals the relative contributions of different osmotica

K^+^ and its counterions, and sugars, mostly sucrose, are the two types of primary osmotica contributing to stomatal opening. As presented earlier, light quality is one of the determining factors of osmotic composition (Figure 4B). In addition, varying the environmental CO_2_ concentration was shown to have an effect on osmotic composition as well: CO_2_-free air or air with low CO_2_ concentration was observed to induce stomatal opening accompanied by K^+^ uptake in response to red light [40]. We systematically investigated the effect of different combinations of light qualities and CO_2_ concentrations on the contribution of each type of osmoticum during stomatal opening (Table 3). Our results indicated that in ambient air, ion accumulation is the predominant mechanism leading to stomatal opening under dual beam or blue light, while sucrose is the major osmoticum during red light-induced stomatal opening. In the case of CO_2_-free air or air with reduced CO_2_ concentration, our model corroborates Olsen et al. (2002) on the importance of K^+^ uptake during red light-induced stomatal opening [40], and predicts the absence of sucrose and the predominance of ion accumulation as an osmoticum under all light qualities. Our model predicts that ion accumulation is severely suppressed in air with elevated CO_2_ concentration, and that under high CO_2_ concentration, sucrose is the primary osmoticum responsible for stomatal opening under all light conditions.

**Table 3:** Simulated stomatal opening levels and osmotic compositions under various conditions of light, CO_2_, and node disruptions.

CO_2_ Content	Light	Phenotype	Maximum Stomatal Opening	t_50%_	t_95%_	Composition of Osmotica	
						Ions (K^+^, Cl^-^, NO_3_^-^, malate^2-^)	Sucrose
Ambient CO_2_	Dual Beam	Wild Type	11.28	6	10	82.5%	17.5%
		H^+^-ATPase KO	2	7	10	0.0%	100.0%
		Sucrose Depletion	9.28	6	9	100.0%	0.0%
	Blue Light	Wild Type	4.15	7	10	75.9%	24.1%
		H^+^-ATPase KO	1	7	10	0.0%	100.0%
		Sucrose Depletion	3.15	7	10	100.0%	0.0%
	Red Light	Wild Type	1	7	9	0.0%	100.0%
		H^+^-ATPase KO	1	7	9	0.0%	100.0%
		Sucrose Depletion	0	0	0	—	—
CO_2_-Free Air	Dual Beam	Wild Type	14.01	6	8	100.0%	0.0%
		H^+^-ATPase KO	0	0	0	—	—
		Sucrose Depletion	14.01	6	8	100.0%	0.0%
	Blue Light	Wild Type	9.28	6	8	100.0%	0.0%
		H^+^-ATPase KO	0	0	0	—	—
		Sucrose Depletion	9.28	6	8	100.0%	0.0%
	Red Light	Wild Type	2	6	7	100.0%	0.0%
		H^+^-ATPase KO	0	0	0	—	—
		Sucrose Depletion	2	6	7	100.0%	0.0%
Elevated CO_2_	Dual Beam	Wild Type	2	7	9	0.0%	100.0%
		H^+^-ATPase KO	2	7	9	0.0%	100.0%
		Sucrose Depletion	0	0	0	—	—
	Blue Light	Wild Type	1	7	9	0.0%	100.0%
		H^+^-ATPase KO	1	7	9	0.0%	100.0%
		Sucrose Depletion	0	0	0	—	—
	Red Light	Wild Type	1	7	9	0.0%	100.0%
		H^+^-ATPase KO	1	7	9	0.0%	100.0%
		Sucrose Depletion	0	0	0	—	—

The CO_2_ conditions studied are: ambient CO_2_ concentration (CO_2_ = 1, top of the table), CO_2_-free air (CO_2_ = 0, middle), and elevated CO_2_ concentration (CO_2_ = 2, bottom). Simulated H^+^-ATPase knockout (H^+^-ATPase_complex_ = 0) severely impairs stomatal opening in the cases where ions are the predominant osmotica, e.g. under CO_2_-free air. Computationally imposed sucrose depletion (sucrose = 0), on the other hand, inhibits stomatal opening in cases where sucrose is the major osmoticum, e.g. under elevated CO_2_ concentration.

We further probed the importance of different types of osmotica by computationally imposing an inhibition of sucrose accumulation (sucrose = 0) or by virtually knocking out the H^+^-ATPase (H^+^-ATPase_complex_ = 0). Our model indicated that in ambient air (Table 3, top), the H^+^-ATPase plays a more important role in dual beam- or blue light-induced stomatal opening than in red light-induced stomatal opening. Conversely, we found that sucrose is more important for red light-induced stomatal opening than for stomatal opening under dual beam or blue light, since its knockout completely inhibits red light-induced stomatal opening while the inhibition is partial for dual beam and blue light. Under CO_2_-free air (Table 3, middle), H^+^-ATPase activity is more critical than sucrose accumulation for stomatal opening under all light conditions. In fact, keeping sucrose at value 0 computationally has no effect on stomatal opening in CO_2_-free air. In air with elevated CO_2_ (Table 3, bottom), the proton pump and henceforth ion accumulation are suppressed, making sucrose the predominant osmoticum for stomatal opening under all light conditions. These results also confirm that the activity of the proton pump is the primary driving force for ion accumulation during stomatal opening [2], [71], [72].

Consistent with the difference in the types of osmotica mediating blue light or red light-induced stomatal opening [6], [35], a K_in_ knockout displayed a more severe reduction of opening level in white light and blue light than in red light [73]. This phenomenon is also captured by our model (Table 4): the simulated white or blue light-induced stomatal opening level decreases dramatically when K_in_ is forced to be 0, but this disruption does not affect the red light-induced stomatal opening level.

**Table 4:** The effect of inward K^+^ channel knockout on stomatal opening under different light conditions predicted by the model.

Light	Phenotype	Maximum Stomatal Opening	t_50%_	t_95%_
Dual Beam	Wild Type	11.28	6	10
	K_in_ KO	2	7	9
Blue Light	Wild Type	4.15	7	10
	K_in_ KO	1	7	9
Red Light	Wild Type	1	7	9
	K_in_ KO	1	7	9

K_in_ knockout has a larger effect in dual beam- and blue light-induced stomatal opening, while it has no observable effect on red light-induced stomatal opening.

### The model predicts the effects of single knockouts

We performed a systematic compilation and comparison of available experimental observations with results generated by our model in a simulation of the experimental conditions. These conditions included different light and/or CO_2_ and/or ABA stimuli and the manipulation of node states by genetic modifications or pharmacological interventions. Sixty-six comparisons were made in total (see Table S4), out of which 64 instances exhibited qualitative consistence between experimental observations and simulation results—a successful validation rate of 97%.

Our model's consistency with known experimental evidence enables confident prediction of new phenotypes. It takes a significant amount of time and effort for experimentalists to investigate the effect of the genetic knockout of even a single element *in vivo*. In contrast, a compilation of the phenotypes of all the single-node knockout phenotypes can be readily obtained *in silico*, and can then be used to inform and prioritize experiments.

*In vivo*, a null phenotype is realized by creating knockout mutants, or by introducing a pharmacological suppressor of a certain element. *In silico*, this is achieved by keeping the level of the ‘knocked-out’ node at 0. We systematically investigated the effect of the knockout of a single node from the system in the following three light conditions: dual beam (blue light = red light = 1), blue light alone (blue light = 1, red light = 0), and red light alone (blue light = 0, red light = 1), and three atmospheric conditions: normal air with moderate CO_2_ concentration (CO_2_ = 1), CO_2_-free air (CO_2_ = 0), and high CO_2_ air (CO_2_ = 2). ABA was set as absent (ABA = 0) in these simulated knockouts, in which each of the 64 internal nodes was individually eliminated *in silico*. Table 5 lists the distribution of stomatal opening levels of the knockout phenotypes as a percentage of the wild type opening, which was equated to 100%. In all three ambient air cases, the majority of the knockouts (67.2% for dual beam, 68.8% for blue light, and 95.3% for red light) maintained an opening level within 5% deviation from wild type opening, demonstrating the robustness of the system against single node loss.

**Table 5:** The distribution of predicted light-induced stomatal opening levels for single node knockouts.

Opening Level As a Percentage of WT Opening		0–5%5%–15%15%–25%25%–35%35%–45%45%–55%55%-65%65%–75%75%–85%85%–95%95%–100%100%–105%											
Light Quality	Air Condition	Percentage of Single Knockouts in Each Bin											
Dual Beam	Moderate CO_2_			17.2%	1.6%	1.6%			1.6%	9.4%	1.6%	64.1%	3.1%
	CO_2_-free	17.2%			3.1%				7.8%	1.6%	1.6%	65.6%	3.1%
	High CO_2_	4.7%										95.3%	
Blue Light	Moderate CO_2_			18.8%		1.6%		1.6%		6.3%	3.1%	68.8%	
	CO_2_-free	17.2%			1.6%		3.1%		6.3%	1.6%	1.6%	65.6%	3.1%
	High CO_2_	4.7%										95.3%	
Red Light	Moderate CO_2_	4.7%										95.3%	
	CO_2_-free	17.2%					3.1%		6.3%	1.6%	1.6%	70.3%	
	High CO_2_	4.7%										95.3%	

Each simulated knockout mutant's opening level is expressed as a percentage of the wild type opening level for the corresponding light quality and CO_2_ condition. ABA is absent in all simulations. The opening levels are binned into 12 ranges, indicated in the header of the table. Each entry indicates the percentage of the 64 knockouts in each opening category. The entry is left blank if no knockout mutant opening level falls in the corresponding range. In the moderate CO_2_ cases, an average of 77.1% of all single knockouts maintains an opening level that is less than 5% different from wild type opening, with less than 2% displaying major inhibition (≥95%) of opening, demonstrating the robustness of the system against single node losses. Single node-knockouts have a larger impact on stomatal opening under CO_2_-free air: an average of 69.3% of all single node-knockouts maintains an opening level less than 5% different from wild type opening, while 17.2% of all knockouts result in major inhibition of stomatal opening in all light conditions. Under high CO_2_ condition, interestingly, all light conditions exhibit identical knockout opening pattern: 95.3% of all single node knockouts display close to wild type opening, and 4.7% display major inhibition.

The knockout of single nodes has a larger impact on stomatal opening under CO_2_-free air, predominantly due to the inactivity of photosynthetic carbon fixation pathways in this condition, making H^+^-ATPase activation and the accumulation of ions crucial to stomatal opening. A smaller fraction of cases maintained an opening within 5% of wild type opening (68.7% for dual beam, 68.7% for blue light, and 70.3% for red light), and 17.2% of all phenotypes resulted in an inhibition of 95% or more of wild type stomatal opening level in all light conditions under CO_2_-free air. Under the high CO_2_ condition, since the proton pump H^+^-ATPase activity is greatly suppressed, stomatal opening under all three light conditions is solely dependent on photosynthesis and sucrose accumulation. Therefore, stomatal opening of knockout phenotypes under the three light conditions have an identical pattern: 95.3% stay close to wild type opening level, and 4.7% display total inhibition of opening.

Interestingly, knocking out the small G protein ROP2 or RIC7 induced a stomatal opening level higher than wild type opening under three different light and CO_2_ conditions (see Table 5, 100%–105%). This model result recapitulates the experimental observation that ROP2 and recruited RIC7 inhibit stomatal opening in wild-type plants, thus providing a protection mechanism against excessive opening [74]. Table S3 provides a full list of stomatal opening levels for each simulated node knockout in each of the nine conditions.

### The model captures the known effects of ABA on stomatal opening and identifies a knowledge gap

It is known that under simultaneous presence of white light and ABA, the latter functions through several secondary messengers, e.g. [Ca^2+^]_c_ [75], [76] and pH_c_ [77], to inhibit light-induced stomatal opening. Our model reproduced this effect as shown in Table 6. ABA decreased stomatal opening under combined blue and red light from 11.28 to 2, and stomatal opening under blue light decreased from 4.15 to 1. Unexpectedly, however, our model predicted that ABA had no inhibitory effect on red light-induced opening, which remained at level 1 regardless of ABA.

**Table 6:** Predicted stomatal opening level under different qualities of light in the absence or presence of ABA.

	ABA Absent			ABA Present		
Light Treatment	Maximum Stomatal Opening	t_50%_	t_95%_	Maximum Stomatal Opening	t_50%_	t_95%_
Dual Beam	11.28	6	10	2	7	9
Blue Light	4.15	7	10	1	7	9
Red Light	1	7	9	1	7	9

The presence of ABA leads to a dramatic decrease in the maximal stomatal opening level under dual beam or blue light but ABA has no effect on stomatal opening under red light.

In the course of the construction of our network of guard cell secondary messengers of light and ABA signaling, we found 30 components (nodes) wherein regulation of the node by both blue light and ABA had been reported or could be inferred, consistent with experimental evidence that ABA inhibits blue light-stimulated stomatal opening [17], and our model clearly indicated ABA-inhibition of blue light stimulated stomatal opening (Table 6, blue light). By contrast, we found no nodes for which regulation of the node by both red light and ABA had been reported. Accordingly, in our model, ABA is predicted to have no effect on red light-induced stomatal opening (Table 6, red light). This prediction led us to extensively peruse the literature for experiments in which ABA inhibition of red light-induced stomatal opening in isolated epidermes had been explicitly assessed, but no such reports were found. This absence of studies perhaps reflects the general (but untested) belief that ABA is able to inhibit light-induced stomatal opening regardless of the wavelength of light. Our identification of this unaddressed question exemplifies how codification of extant knowledge into network models can suggest key new experiments.

### Experimental test of the effect of ABA on red light-induced stomatal opening

**Figure 6:**
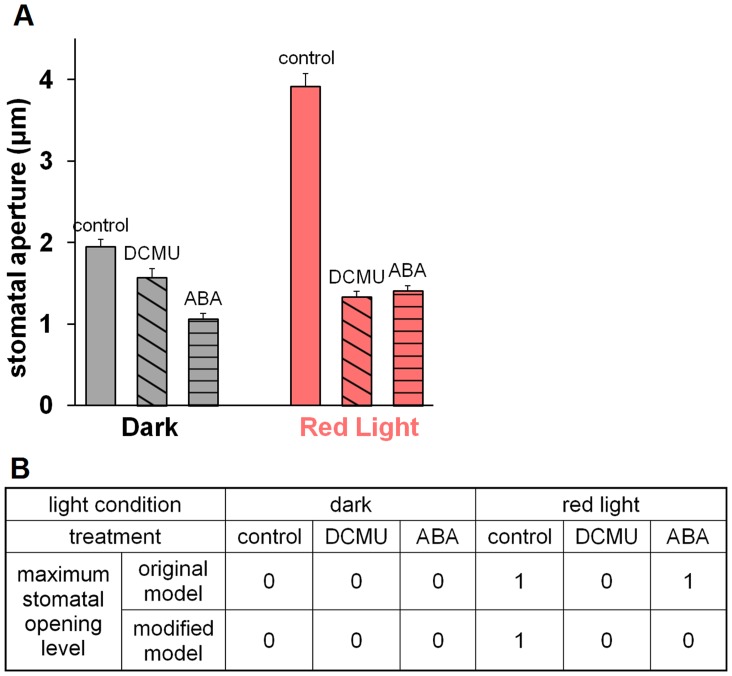
The effect of ABA and DCMU on red light-induced stomatal opening. (A) Experimental measurement of stomatal apertures in isolated epidermal peels of *Vicia faba* under different conditions. Qualitatively, the apertures can be categorized into two opening levels: red light yields a high opening level, and opening levels under all other conditions can be considered as low. (B) Simulated stomatal opening levels. In the original model, stomatal opening levels under red light and red light + ABA treatments are high (level 1); all other treatments yield low (level 0) opening levels. If the model is modified by adding an inhibition of sucrose accumulation by ABA, red light is the only condition that yields a high opening level; all other conditions have low opening levels. This is in qualitative agreement with the experimental results shown in (A).

Accordingly, we experimentally assessed the effect of ABA on red light-induced stomatal opening in *Vicia faba* epidermal peels (see Materials and Methods). Significant inhibition by ABA of red light-induced stomatal opening was found (Figure 6A). We also observed inhibition of stomatal opening by the photosynthetic inhibitor, DCMU, consistent with a previous report [3].

Qualitatively, the average stomatal aperture values from the wet bench experiments can be divided into two groups: those that have a high value, of which red light treatment is the only instance, and those that have a low value, which contains all the other experimental conditions. Figure 6B shows the stomatal opening levels obtained from our model for the same conditions as those studied experimentally. As our model, constructed based on available knowledge, lacks a mechanism through which ABA can inhibit red light-induced opening, combined action of red light and ABA results in a high opening level equal to that of red light alone, while all other cases have low opening levels. The model predictions are qualitatively consistent with the experimental findings with the exception of the case of combined red light and ABA input. The discrepancy between the model and experimental results in the latter case points out a missing piece of the current knowledge base of the model: a mechanism through which ABA can inhibit red light-induced stomatal opening.

### A hypothesis on ABA inhibition of red light-induced stomatal opening

The question ‘how does ABA inhibit red light-induced stomatal opening?’ remains open. Since sucrose is the major osmoticum accumulated under red light in ambient CO_2_, a natural first hypothesis is that ABA inhibits sucrose accumulation. This hypothesis is supported by our result that there are only three nodes whose individual knockout abolishes red light-induced stomatal opening: light reaction, carbon fixation and sucrose (see Table S3). These nodes form a linear path from ABA to sucrose (see Figure 2). ABA could disrupt the reaction cascade through which sucrose is generated, or cause the conversion of sucrose into starch, or promote sucrose catabolism within the guard cell or its efflux from the cell.

To explore the explanatory power of a putative ABA inhibition of sucrose accumulation, we modified our model by adding an inhibitory edge from ABA to sucrose and adding the Boolean clause “*And Not* ABA” to the existing rule for sucrose. The simulation result of this modified model is shown in Figure 6B. Notably, after this modification, ABA is able to inhibit red light-induced stomatal opening. The qualitative response pattern across all treatments matches the experimental results. Further, we assessed the impact of the putative inhibitory edge from ABA to sucrose on the overall performance of our model by comparing all the simulation results obtained from the modified model with those obtained from the original model. We were able to confirm that all results stay either identical (e.g., for conditions where the ABA signal is absent) or qualitatively consistent (for conditions where the ABA signal is present, see Table S5). Importantly, this exemplifies how discrete models, even in the absence of complete knowledge of all interactions and of temporal signaling dynamics, can be readily employed to test new hypotheses and putative pairwise relationships between components of the system.

## Discussion

Our model offers a comprehensive and systematic description of the process of light signal transduction in guard cells and its crosstalk with CO_2_ and ABA. The network representation we employ reveals the regulatory connections between seemingly remote components. For example, a recent publication, which was not included during our construction of the model, studied the indirect relationship between the SLAC1 anion channel and the regulation of K^+^ uptake [22]. It reported that anion accumulation in the *slac1* anion channel knockout mutant induced the hyperpolarization of the plasma membrane which in turn promoted Ca^2+^ influx. Ca^2+^ influx led to an increase in the free cytosolic Ca^2+^ concentration, which then downregulated the inward K^+^ channels. This relationship is supported by a path in our network, namely AnionCh→PMV–•CaIC→[Ca^2+^]_c_–•K_in_. Structural analysis of the network provided significant biological insight. The node degree offers a measure of node importance (Table 1) and path analysis reveals the robustness of the crosstalk between ABA and blue light in regulating stomatal opening. Our strongly connected component analysis identified key components that mediate the cross-talk between blue light and ABA, such as [Ca^2+^]_c_ (Figure 2C), and highlighted the absence of cross-talk between red light and ABA (Figure 2A). The latter observation, recapitulated by our dynamic model, revealed the absence of experimental investigations of regulatory effects of ABA on red light-induced stomatal opening. Experiments performed in this study fill this knowledge gap and reveal that ABA does in fact inhibit red light-induced stomatal opening. We formulate the novel hypothesis that ABA inhibits sucrose accumulation, and demonstrate that integration of this hypothesis into the model restores the agreement between model and experiments.

Our model reveals additional questions where further experimental investigation would be especially fruitful. We discuss a few such examples below. The model can offer a prediction for outcomes based on integration of our current knowledge, but it is up to future experiments to answer these questions definitively.

There has been a long debate on whether the plasma membrane H^+^-ATPase is active under red light alone. The evidence regarding the status of the H^+^-ATPase during red light-induced stomatal opening is mixed: activation of the proton pump by red light has been observed [78] or inferred [40], but the results in [78] were not reproduced [64], [79] and the experiment in [40] was done under reduced CO_2_ concentration. Little or no activation of the proton pump by red light has been observed in other experiments [18], [80]. Our model predicts the inactivity of the H^+^-ATPase under red light in ambient air and predicts a moderate H^+^-ATPase activity under red light in a reduced CO_2_ condition (see Figure 5B). Experiments dedicated to measuring the H^+^-ATPase activity under red light with varying CO_2_ concentrations will greatly improve our understanding of this matter.

A remaining question about sucrose as an osmoticum is the relative contribution of different sources of guard cell sucrose accumulation under different light and CO_2_ conditions. Sugar accumulation, predominantly sucrose [35], [81], can in theory result from photosynthetic carbon fixation, degradation of stored starch [6], [81], [82], or import from the apoplast [34], [83], [84]. These three processes exhibit different responsiveness to light and ABA. Photosynthesis is activated by either blue or red light, requires CO_2_, and was not observed to be inhibited by ABA [47] (see Figure 2). Starch content was shown to be constant under red light but decreasing in time under blue light [6]. The H^+^/sucrose symporter function requires apoplast acidification by the H^+^-ATPase [34], [83], which is not effective under red light in ambient air. Since ABA inhibits the plasma membrane H^+^-ATPase [51], [63], [85] and apoplast acidification by the H^+^-ATPase is required by the H^+^/sucrose symporter, it can be inferred that the symporter activity can be inhibited by ABA. In our model we assumed that carbon fixation is the primary source of sucrose accumulation (see Text S1 for a detailed justification). Experiments that dissect the contribution of each source of guard cell sucrose accumulation will not only help improve the model, but also provide insight into the interaction between blue and red light and between light and ABA in the regulation of stomatal movement.

Our simulations showed that the inactivation of photophosphorylation, e.g. by DCMU, induces a significant reduction in stomatal opening under all three light quality conditions (Table 2A). Our model predicts that the inactivation of photophosphorylation i) reduces carbon fixation and hence the amount of sucrose accumulated via photosynthesis, and ii) reduces the amount of ATP available for H^+^-ATPase activity. Since the H^+^-ATPase is not activated by red light in ambient air in our model, our simulations suggest that DCMU inhibits red light-induced stomatal opening through mechanism i) only. Since the H^+^-ATPase is activated by blue light in ambient air, our simulations suggest that DCMU inhibits blue light-induced stomatal opening through both mechanism i) and ii). Experiments showed that DCMU partially inhibited blue light-induced stomatal opening and it completely inhibited red light-induced stomatal opening [3], [80]; it would be informative to investigate the effect of DCMU on dual beam- or white light-induced stomatal opening as well. Further, it would also be interesting to explore the effect of DCMU and the respiratory inhibitor potassium cyanide (KCN) on light-induced stomatal opening in CO_2_-free air, a condition under which photosynthetic carbon fixation is absent, as CO_2_, the substrate for carbon fixation, is unavailable.

Our model implements a brake-like effect of high C_i_ on H^+^-ATPase activity based on the observation that an enhanced level of CO_2_ depolarizes the plasma membrane [86] and the consequent hypothesis that elevated CO_2_ inhibits the proton pump at the plasma membrane. This inhibitory effect of C_i_ on the H^+^-ATPase activity helps to explain the activation of the H^+^-ATPase under low CO_2_ conditions [40]. Understanding the mechanism underlying this effect of C_i_ on the H^+^-ATPase and in particular, whether it is a direct or an indirect effect, would provide valuable information. Such data would not only clarify the relationship between light (especially red light) and the H^+^-ATPase activity level, but also offer insight into the synergy between blue and red light. According to our model, red light as a background to blue light can not only provide additional ATP through photophosphorylation for the H^+^-ATPase activity, but also lower C_i_ via stimulation of mesophyll photosynthesis and thus raise the activity of the H^+^-ATPase (Figure 5B). These two mechanisms could be critical in explaining the synergy between blue and red light in the intact leaf. Our model also predicts that ion uptake/accumulation, which hinges upon H^+^-ATPase activity, is the primary mechanism for stomatal opening in response to red light under CO_2_-free air (Table 3). Therefore, stomatal opening of a K_in_ knockout phenotype in response to red light with CO_2_-free air should be severely impaired, in contrast to the minimal effect of K_in_ knockout on red light-induced stomatal opening under normal air (Table 4 and [73]). Experimental verification of this prediction would also support the model's predictions regarding the osmotic composition during stomatal opening in CO_2_-free air (Table 3), and provide further evidence for the activation of the H^+^-ATPase by red light in CO_2_-free air as proposed in [40].

The current model offers a qualitatively accurate and quantitatively close depiction of short term stomatal movement in response to a light signal. There have been investigations, however, which demonstrate that under natural conditions (white light) sucrose accumulates in guard cells in the afternoon and replaces K^+^ as the dominant osmoticum to maintain stomatal apertures [6]. An interesting potential future direction for our model is the incorporation of emerging knowledge concerning the cross-talk of guard cell circadian rhythms, light and ABA responses (e.g. [87]). The recent successful Boolean model of circadian clocks [88] makes the construction of such an integrated gene regulatory and signal transduction network model feasible.

Our modelling framework characterizes each component with two or more levels and expresses the relationships between components as a mixture of logical rules and algebraic operations. Thus our model offers a parsimonious, computationally efficient yet quantitative description of the system's dynamics, making it a step forward from traditional Boolean models and an enhanced modelling tool for systems biology. Our choices of the update functions of the nodes are a simplified and abstracted representation of the best available knowledge. Assuming discrete node levels is an approximation of reality (e.g. the concentration of a substance or the potential across the plasma membrane is continuous in reality); there is, however, ample evidence of nonlinear regulation wherein not the concentration but rather its relationship with certain thresholds matters. Network-based discrete dynamic modelling has been successfully applied in a great variety of biological systems (reviewed in [89]–[92]). These models enabled the understanding of the systems and generated insightful predictions that were subsequently validated experimentally; recent examples include [24], [93]–[97].

In the absence of detailed knowledge on relative reaction speeds we deliberately sampled different timescales by implementing random order asynchronous update. While this is not a fully accurate representation of reality, the averaged results of a large number of replicate simulations are representative of behaviors that are not sensitive to small changes in kinetic rates. Future observations of relative temporal patterns of multiple components or measurements of time delays among components can be incorporated by imposing restrictions on the update sequence (e.g. updating one group of nodes before another group [91]).

Having established the biological validity of our model, follow-up work in several directions is now possible, linking to recent advances in discrete and continuous dynamic modelling. Translating the model into a polynomial discrete dynamic system [98] or logical discrete model [99] would allow the use of software tools such as ADAM [100] or GINsim [99], and may yield further insights into the dynamic repertoire of the system. Our model could also be translated into a Boolean model of an expanded network, where multi-level components are represented by multiple nodes in such a way that the group of binary nodes representing the same component allows the recapitulation of the same number of relative outcomes as the original multi-level node (see e.g. [97]). This transformation would allow the application of Boolean network analyses such as elementary signalling mode analysis and attractor analysis [91]. Through network simplification methods [30], [101], a core network with fewer nodes and edges could be distilled, which may be amenable for continuous modelling wherein differential equations replace the update functions. For instance, a model that integrates a simplified version of our model with OnGuard [20] would include the various signal transduction pathways, their cross-talk, and the quantitative description of ion flows in guard cells.

Our model is generically adaptable, allowing one to incorporate emerging new pieces of information with ease. This modeling methodology can be readily applied to other systems where interaction and relative state information is available. The number of multi-level nodes can be minimized by identifying the node(s) for which more than three relative outcomes have been observed (indicative of a need for more than two levels), tracing upstream in the network, and inferring a minimal set of nodes whose multi-level nature could cause all the other nodes' multiple levels. The states of this set of nodes can then be defined as fundamental states (see Text S1), and the states of other nodes subsequently derived through their updating rules. As demonstrated here, network-based dynamic models of biological systems can serve as a virtual control to test the coherence between experimental results generated in separate experiments, to generate predictions that inform and help prioritize future experiments, and to reveal new questions that deserve attention.

## Materials and Methods

### Plant growth and stomatal aperture measurements

Plants of *Vicia faba* L., cv. ‘Long pod’ were grown from seeds on Metro Mix 300 soil media (Griffin Greenhouse Supplies, Inc., Morgantown, PA) in a reach-in growth chamber with 120 µmol m^−2^ s^−1^ light, with 8 h day/16 h night cycles and 22°C/20°C day/night temperatures. The plants were watered with deionized water once a week and fertilized once a week (on a different day) with half-strength Hoagland's solution. Approximately 20 healthy and young fully-expanded leaflets from 3 week old plants were selected for stomatal aperture measurements.

Epidermal peels from the abaxial side were stripped from interveinal regions using forceps and blended in a buffer (10 mM MES, 5 mM CaCl_2_, 0.1% polyvinylpyrrolidone PVP40, adjusted to pH 6 using 1 M KOH) for 5 seconds. Blended peels of uniform size were distributed among the wells of each of two multi-well Petri dishes, each well containing 0.5 mM CaCl_2_, pH 6.15, and placed in the dark for 1 hour to close the stomata initially. Then, 0.1% ethanol (solvent control), 50 µM ABA in ethanol, or 20 µM DCMU in ethanol (final concentrations) was added to individual wells. One Petri dish containing peels with these treatments was transferred to red light (125 µmol m^−2^ s^−1^) for 3 hours. The other Petri dish was wrapped in foil and kept in darkness for 3 hours as a dark control. The red light source was an InFocus LP500 projector (InFocus Corp., Portland, OR) combined with a red filter transmitting light above 600 nm (Ridout Plastics Co. Inc.).

Seventy to two hundred stomatal apertures were measured per replicate. Table S6 contains all the experimentally measured stomatal apertures; the values in Figure 6A are means ± standard errors from three independent replicates. All images were taken at 400x total magnification using a Nikon DIPHOT 300 microscope (Nikon, Japan) connected to a Nikon E990 camera (Nikon, Japan). All stomatal apertures were measured using the free access software ImageJ (http://rsb.info.nih.gov/ij/), version 1.39u. Treatments were blinded during image acquisition and analysis.

### Constructing update functions for binary nodes

When there is only one upstream regulator of the target, the “*Equal*” rule is used for positive regulation and the “*Not*” rule is used for negative regulation. The “*AND*” operator is used when multiple regulators are required to activate the target. If each regulator is able to independently activate the target, they are connected with the “*OR*” operator. For inhibition, the “*AND NOT*” operator is used, thereby requiring a low level or inactivity of the inhibitor in order for the target node to activate. One example of a Boolean update function is shown below:

phot1_complex_^*^  =  phot1 *And* 14-3-3 protein_phot1_

Phototropin 1 binds reversibly to a 14-3-3 protein (14-3-3 protein_phot1_) upon the auto-phosphorylation of phototropin 1 in guard cells [102], [103]. The 14-3-3 protein-phototropin complex (phot1_complex_) is thought to confer an active state to phototropin 1, which then transmits the light signal to downstream elements. The *And* function connects phot1 and the 14-3-3 protein, indicating that the formation of the complex requires both of them.

### Constructing update functions for nodes with more than two levels

The update functions combine Boolean clauses with addition, subtraction, and multiplication. One example of a complex update function is given below:

PMV^*^  =  PMV – H^+^-ATPase_complex_ + (AnionCh *And* (PMV = –2)) + (([Ca^2+^]_c_ = 2) *Or* KEV)

PMV is the difference of electric potential across the plasma membrane, i.e. the “membrane potential”. Computationally, we use the value 0 to represent the resting potential of the plasma membrane. Negative values denote the further hyperpolarization of the plasma membrane, and positive values denote depolarization. We assume that five levels (−2, −1, 0, 1, 2) are sufficient for a qualitative description, and require that the PMV value stays bounded, i.e. the value will not further decrease (or increase) when it reaches −2 (or 2). The future PMV value (PMV^*^) can be shifted away from or stay the same as its current value (PMV), depending on the hyperpolarizing and depolarizing forces. Factors that cause hyperpolarization will decrease the PMV value (e.g. from 0 to −1), and factors that cause depolarization will increase the PMV value (e.g. from 0 to 1). An active 14-3-3 protein-bound H^+^-ATPase causes the extrusion of H^+^ from guard cell cytosol to the apoplast, hyperpolarizing the plasma membrane. Anion efflux at the plasma membrane causes the plasma membrane to depolarize. A steady anion efflux requires AnionCh to be active and it also requires plasma membrane depolarization. To avoid an oscillation in anion efflux and PMV induced by the discrete nature of the model, we require that PMV be -2 (most hyperpolarized) for effective anion efflux. A high [Ca^2+^]_c_ concentration (value 2) or K^+^ release into the cytosol from vacuole (KEV) are modelled as independent factors causing the plasma membrane to depolarize. Text S5 indicates the pseudo-code for the implementation of two representative update functions.

## Computational implementation and tools

The network in Figure 1 was drawn with the software yED (http://www.yworks.com/en/products_yed_about.html). The network analyses (strongly connected component identification, calculation of the number of simple paths between two nodes) were implemented by custom MATLAB code. Similar analyses can also be done with one of the following tools: NetworkX, a Python graph software library [104], Cytoscape, a network integration, visualization and analysis tool [105], or MATLAB's graph theory toolbox, grTheory.

The dynamic model was implemented by custom MATLAB code. The pseudo-code of the simulations is indicated in Text S5.

## Supporting Information

Table S1 **Compilation of the pairwise interactions and regulations which are represented as edges in the network.**
(DOCX)

Table S2 **The effect of single edge removal on strongly connected component 1 (SCC1).**
(XLSX)

Table S3 **Stomatal opening levels for simulated single node knockouts.**
(XLSX)

Table S4 **Compilation of comparisons between published experimental observations and the model's results for simulations of the identical conditions.**
(DOCX)

Table S5 **Compilation of comparisons between published experimental observations and the modified model's results for simulations of the identical conditions.**
(DOCX)

Table S6 **Experimentally measured stomatal apertures in isolated epidermal peels of** ***Vicia faba*** **under different conditions.**
(XLS)

Text S1 **Description of node levels and updating rules.**
(DOCX)

Text S2 **Example of network construction.**
(DOCX)

Text S3 **Description of the network.**
(DOCX)

Text S4 **Evaluating the model's robustness.**
(DOCX)

Text S5 **Pseudo-code for stomatal opening simulations.**
(DOCX)

Text S6 **List of references that appear in the supporting information files.**
(DOCX)
