# Correction: Multi-level Modeling of Light-Induced Stomatal Opening Offers New Insights into Its Regulation by Drought

**DOI:** 10.1371/journal.pcbi.1005181

**Published:** 2016-11-30

**Authors:** The PLOS Computational Biology Staff

S4 Table contains an error in the simulated stomatal opening value for PIP2 knockout mutants under white light. The correct value, Stomatal opening = 2, makes the simulated result inconsistent with the experimental observation. The correct model validation rate, and corrected last sentence of the first paragraph of the Results subsection “The model predicts the effects of single knockouts” are as follows:

“Sixty-six comparisons were made in total (see S4 Table), out of which 63 instances exhibited qualitative consistence between experimental observations and simulation results—a successful validation rate of 95%.”

S4 Table and S1 Text also include some minor typographical errors, which do not affect the validity of the results.

Please view the correct versions of S4 Table and S1 Text here:

## Supporting Information

S1 Text Description of node levels and updating rules. (DOCX)

S4 Table Compilation of comparisons between published experimental observations and the model's results for simulations of the identical conditions. (DOCX)
