# Supplementary material for: Multi-level Modeling of Light-Induced Stomatal Opening Offers New Insights into Its Regulation by Drought
Source: PLoS Comput Biol. 2014 Nov 13;10(11):e1003930. doi: 10.1371/journal.pcbi.1003930 (PMC4230748; doi:10.1371/journal.pcbi.1003930)
Supplement: Table S1 — Compilation of the pairwise interactions and regulations which are represented as edges in the network. (DOCX) [file pcbi.1003930.s001.docx]

**Table S1. Compilation of the pairwise interactions and regulations which are represented as edges in the network**

Abbreviations: *As, Avena sativa* L.*; At, Arabidopsis thaliana; Cb, Commelina benghalensis* L.*; Cc, Commelina communis* L.*; So, Spinacia oleracea; Tv, Tradescantia virginiana* L.*; Vf, Vicia faba* L.

| **Regulator** | **Target Node** | **Direct Interaction?** | **Regulation** | **Species** | **References** |
| --- | --- | --- | --- | --- | --- |
| blue light | phot1 | yes | activation | *At* | [14-16] |
| blue light | phot2 | yes | activation | *At* | [14-16] |
| phot1 | phot1_complex_ | yes | activation | *At, Vf* | [102, 103] |
| 14-3-3 protein_phot1_ | phot1_complex_ | yes | activation | *Vf* | [102] |
| ROS | CaIC | no | activation | *Cc* | [43] |
| PMV | CaIC | no | activation | *Cc* | [43] |
| NO | CaR | no | activation | *At* | [S1] |
| PLC | CaR | no | activation | *At* | [S1] |
| phot2 | PLC | no | activation | *At, Cc* | [43, S1, S63] |
| ABA | PLC | no | activation | *At, Cc* | [43, S1, S63] |
| [Ca^2+^]_c_ | PLC | no | activation | *At, Cc* | [43, S1, S63] |
| PLA_2_β | LPL | yes | activation | *As* | [S37] |
| PLA_2_β | FFA | yes | activation | *As* | [S37] |
| phot1_complex_ | PLA_2_β | no | activation | *At* | [S2] |
| phot2 | PLA_2_β | no | activation | *At* | [S2] |
| red light | PLA_2_β | no | activation | *At* | [S2] |
| nitrate | nitrite | yes | activation | *At* | [S3] |
| NIA1 | nitrite | yes | activation | *At* | [S3] |
| Atnoa1 | NO | no | activation | *At* | [S5, S71] |
| nitrite | NO | yes | activation | *At* | [S3] |
| NADPH | NO | yes | activation | *At* | [S4] |
| NIA1 | NO | yes | activation | *At* | [S3] |
| ROS | NIA1 | no | activation | *At, Vf* | [S4, S7] |
| CaIC | [Ca^2+^]_c_ | yes | activation | *At* | [S8] |
| CaR | [Ca^2+^]_c_ | no | activation | *At* | [S8] |
| Ca^2+^-ATPase | [Ca^2+^]_c_ | yes | inhibition |  | [33] |
| ABA | [Ca^2+^]_c_ | no | activation | *Cc* | [43, S9] |
| [Ca^2+^]_c_ | Ca^2+^-ATPase | yes | activation |  | [33] |
| [K^+^]_v_ | KEV | yes | activation |  | [33] |
| [Ca^2+^]_c_ | KEV | no | activation | *At* | [S10-S12] |
| phot1_complex_ | PRSL1 | no | activation | *At* | [S15] |
| phot2 | PRSL1 | no | activation | *At* | [S15] |
| PP1_cn_ | PP1_cc_ | no | activation | *At* | [S15] |
| PRSL1 | PP1_cc_ | yes | activation | *At, Vf* | [S13-S15] |
| PA | PP1_cc_ | no | inhibition | *Vf* | [17] |
| PIP2_c_ | PIP2_PM_ | no | activation | *At, Cc, Vf* | [S16] |
| phot1_complex_ | PIP2_PM_ | no | activation | *At, Cc, Vf* | [S16, S57] |
| phot2 | PIP2_PM_ | no | activation | *At, Cc, Vf* | [S16, S57] |
| PLD | PA | yes | activation | *At* | [S78] |
| ABA | PLD | no | activation | *At, Vf* | [42, S17, S18] |
| NO | PLD | no | activation | *Vf* | [S17] |
| ABA | ABA receptor | yes | activation | *At* | [S19-S25, S79] |
| ABA receptor | ABI1 | yes | inhibition | *At* | [S19, S21, S22] |
| ABI1 | OST1 | yes | inhibition | *At* | [S27, S28] |
| phot1_complex_ | ROP2 | no | activation | *At* | [74] |
| phot2 | ROP2 | no | activation | *At* | [74] |
| red light | ROP2 | no | activation | *At* | [74] |
| PA | ROP2 | no | activation | *At* | [S29] |
| OST1 | AtrbohD/F | yes | activation | *At* | [S30] |
| PA | AtrbohD/F | no | activation | *At* | [S31] |
| ROP2 | AtrbohD/F | no | activation | *At* | [S29] |
| NADPH | ROS | yes | activation |  | [33] |
| AtrbohD/F | ROS | yes | activation |  | [33] |
| CDPK | ROS | no | activation | *At* | [S32] |
| Atnoa1 | ROS | no | inhibition | *At* | [S33, S34] |
| photophosphorylation | ATP | no | activation |  | [33] |
| mitochondria | ATP | no | activation |  | [33] |
| C_i_ | protein kinase | no | inhibition | *Tv* | [86] |
| PP1_cc_ | protein kinase | no | activation | *Vf* | [S13, S14] |
| protein kinase | H^+^-ATPase | yes | activation | *Vf, So* | [36, S35] |
| H^+^-ATPase | 14-3-3 protein_H+-ATPase_ | yes | activation | *At, Vf* | [65, S36] |
| FFA | H^+^-ATPase_complex_ | no | activation | *At, As* | [S2, S37] |
| LPL | H^+^-ATPase_complex_ | no | activation | *At, As* | [S2, S37] |
| 14-3-3 protein_H+-ATPase_ | H^+^-ATPase_complex_ | yes | activation | *At, Vf* | [65, S36] |
| H^+^-ATPase | H^+^-ATPase_complex_ | yes | activation | *At, Vf* | [65, S36] |
| ATP | H^+^-ATPase_complex_ | yes | activation | *Vf* | [4, 80] |
| [Ca^2+^]_c_ | H^+^-ATPase_complex_ | no | inhibition | *Vf* | [56] |
| [Ca^2+^]_c_ | CDPK | yes | activation | *Vf* | [54] |
| ATP | CDPK | yes | activation | *Vf* | [54] |
| H^+^-ATPase_complex_ | PMV | no | inhibition |  | [33] |
| AnionCh | PMV | no | activation |  | [33] |
| [Ca^2+^]_c_ | PMV | no | activation |  | [33] |
| KEV | PMV | no | activation |  | [33] |
| blue light | photophosphorylation | no | activation |  | [33] |
| red light | photophosphorylation | no | activation |  | [33] |
| photophosphorylation | NADPH | no | activation |  | [33] |
| CO_2_ | carbon fixation | no | activation |  | [33] |
| C_i_ | carbon fixation | no | activation |  | [33] |
| photophosphorylation | carbon fixation | no | activation |  | [33] |
| H^+^-ATPase_complex_ | acid. apo | no | activation | *At, Vf* | [37, 51, 63] |
| acid. apo | AtSTP1 | no | activation | *At* | [84] |
| carbon fixation | sucrose | no | activation | *Vf* | [6, 33, 81] |
| FFA | K_in_ | no | activation | *At* | [S2, S39] |
| [Ca^2+^]_c_ | K_in_ | no | inhibition | *Vf* | [55, S40] |
| ABA | K_in_ | no | inhibition | *Vf* | [S40] |
| C_i_ | K_in_ | no | inhibition | *Vf* | [S42] |
| PMV | K_in_ | no | inhibition | *Vf* | [S43] |
| ABA | K_out_ | no | activation | *Vf* | [S44, S45] |
| C_i_ | K_out_ | no | activation | *Vf* | [S42] |
| ROS | K_out_ | no | inhibition | *Vf* | [S46] |
| NO | K_out_ | no | inhibition | *Vf* | [S47] |
| FFA | K_out_ | no | inhibition | *At* | [S2, S39] |
| PMV | K_out_ | no | activation | *Vf* | [S43] |
| K_in_ | [K^+^]_c_ | yes | activation |  | [33] |
| KEV | [K^+^]_v_→[K^+^]_c_ | no | activation |  | [33] |
| [K^+^]_v_ | [K^+^]_c_ | no | activation |  | [33] |
| H^+^-ATPase_complex_ | [K^+^]_c_ | no | activation | *Vf* | [37, 38] |
| K_out_ | [K^+^]_c_ | yes | inhibition |  | [33] |
| [K^+^]_c_ | [K^+^]_v_ | no | activation |  | [33] |
| [NO_3_^-^]_c_ | [NO_3_^-^]_a_ | no | activation |  | [33] |
| AnionCh | [NO_3_^-^]_c_→[NO_3_^-^]_a_ | no | activation |  | [33] |
| PMV | [NO_3_^-^]_c_→[NO_3_^-^]_a_ | no | activation |  | [33] |
| [NO_3_^-^]_a_ | [NO_3_^-^]_c_ | no | activation | *At* | [S48] |
| CHL1 | [NO_3_^-^]_a_→[NO_3_^-^]_c_ | yes | activation | *At* | [S48] |
| [K^+^]_c_ | [NO_3_^-^]_c_ | no | activation |  | [33] |
| AnionCh | [NO_3_^-^]_c_ | yes | inhibition |  | [33] |
| PMV | [NO_3_^-^]_c_ | no | inhibition |  | [33] |
| [NO_3_^-^]_c_ | [NO_3_^-^]_v_ | no | activation |  | [33] |
| [K^+^]_c_ | [Cl^-^]_c_ | no | activation |  | [33] |
| AnionCh | [Cl^-^]_c_ | yes | inhibition |  | [33] |
| PMV | [Cl^-^]_c_ | no | inhibition |  | [33] |
| CDPK | [Cl^-^]_v_ | no | activation | *Vf* | [54] |
| [Cl^-^]_c_ | [Cl^-^]_v_ | no | activation | *Vf* | [54] |
| ABA | PEPC | no | inhibition | *Vf* | [S50] |
| H^+^-ATPase_complex_ | PEPC | no | activation | *Vf* | [S50, S51] |
| blue light | mesophyll cell photosynthesis | no | activation |  | [33] |
| red light | mesophyll cell photosynthesis | no | activation |  | [33] |
| C_i_ | mesophyll cell photosynthesis | no | activation |  | [33] |
| mesophyll cell photosynthesis | [malate^2-^]_a_ | no | activation |  | [33] |
| [malate^2-^]_c_ | [malate^2-^]_a_ | no | activation |  | [33] |
| AnionCh | [malate^2-^]_c_→[malate^2-^]_a_ | no | activation |  | [33] |
| PMV | [malate^2-^]_c_→[malate^2-^]_a_ | no | activation |  | [33] |
| CO_2_ | C_i_ | yes | activation |  | [33] |
| carbon fixation | C_i_ | no | inhibition |  | [33] |
| mesophyll cell photosynthesis | C_i_ | no | inhibition |  | [33] |
| [Ca^2+^]_c_ | AnionCh | no | activation | *At, Vf* | [55, S53] |
| ABA | AnionCh | no | activation | *At, Vf* | [55, S53] |
| ABI1 | AnionCh | no | inhibition | *At* | [S54] |
| PIP2_PM_ | AnionCh | no | inhibition | *At, Cc, Vf* | [S16] |
| C_i_ | AnionCh | no | activation | *Vf* | [S42, S55, S56] |
| [malate^2-^]_a_ | AnionCh | no | activation |  | [S52] |
| starch | [malate^2-^]_c_ | no | activation |  | [33] |
| carbon fixation | [malate^2-^]_c_ | no | activation |  | [33] |
| PEPC | [malate^2-^]_c_ | no | activation |  | [33] |
| [malate^2-^]_a_ | [malate^2-^]_c_ | no | activation | *At* | [S58] |
| AtABCB14 | [malate^2-^]_c_ | no | activation | *At* | [S58] |
| [K^+^]_c_ | [malate^2-^]_c_ | no | activation |  | [33] |
| mitochondria | [malate^2-^]_c_ | no | inhibition | *Cc* | [S49] |
| ABA | mitochondria─●[malate^2-^]_c_ | no | activation | *Cc* | [S49] |
| AnionCh | [malate^2-^]_c_ | yes | inhibition |  | [33] |
| PMV | [malate^2-^]_c_ | no | inhibition |  | [33] |
| [malate^2-^]_a_ | AtABCB14 | no | activation | *At* | [S58] |
| CDPK | [malate^2-^]_v_ | no | activation | *Vf* | [54] |
| [malate^2-^]_c_ | [malate^2-^]_v_ | no | activation | *Vf* | [54] |
| [malate^2-^]_c_ | starch | no | activation | *Cc* | [S49] |
| ABA | [malate^2-^]_c_→starch | no | activation | *Cc* | [S49] |
| ROP2 | RIC7 | yes | activation | *At* | [74] |
| [K^+^]_v_ | stomatal opening | no | activation |  | [33] |
| [Cl^-^]_v_ | stomatal opening | no | activation |  | [33] |
| [NO_3_^-^]_v_ | stomatal opening | no | activation |  | [33] |
| [malate^2-^]_v_ | stomatal opening | no | activation |  | [33] |
| sucrose | stomatal opening | no | activation | *Cb* | [34] |
| RIC7 | stomatal opening | no | inhibition | *At* | [74] |
