# Supplementary material for: Multi-level Modeling of Light-Induced Stomatal Opening Offers New Insights into Its Regulation by Drought
Source: PLoS Comput Biol. 2014 Nov 13;10(11):e1003930. doi: 10.1371/journal.pcbi.1003930 (PMC4230748; doi:10.1371/journal.pcbi.1003930)
Supplement: Table S5 — Compilation of comparisons between published experimental observations and the modified model's results for simulations of the identical conditions. (DOCX) [file pcbi.1003930.s005.docx]

**Table S5. Compilation of comparisons between published experimental observations and the simulation results by the modified model of the identical conditions.** The modified model contains one additional edge (ABA -| sucrose) compared to the original model to explore the explanatory power of a putative ABA inhibition of sucrose accumulation. The simulation results generated by the modified model in the cases where ABA is not involved in the experimental condition stay the same as the results of the original model shown on Table S4. These cases are not included in this table to avoid redundancy. In the column entitled Simulation Settings “BL” stands for blue light, “RL” stands for red light, “ABA” means abscisic acid, “CO_2_” indicates atmospheric CO_2_, and “C_i_” indicates intercellular CO_2_. The column entitled Results indicates the mean level (based on 2000 simulations) of the corresponding variable at steady state (after 18 time steps), unless indicated otherwise. The last column is a qualitative conclusion whether the simulations yield consistent results with experimental observations (the column title, C stands for "Consistency"). The qualifier "C" in individual entries (meaning "consistent") indicates that the model qualitatively recapitulates the experimental observation. PC stands for partially consistent.

| **Experimental Observation** | **References** | **Simulation Settings** | **Results** | **C** |
| --- | --- | --- | --- | --- |
| ABA inhibits white light-induced stomatal opening. | [2, 18, 41, 43, S78] | White light without ABA: BL=1, RL=1, ABA=0, CO_2_=C_i_=1 | Stomatal opening=11.28 | C |
|  |  | White light with ABA: BL=1, RL=1, ABA=1, CO_2_=C_i_=1 | Stomatal opening=0 |  |
| ABA inhibits blue light-induced stomatal opening. | [17] | Blue light without ABA: BL=1, RL=0, ABA=0, CO_2_=C_i_=1 | Stomatal opening=4.15 | C |
|  |  | Blue light with ABA: BL=1, RL=0, ABA=1, CO_2_=C_i_=1 | Stomatal opening=0 |  |
| ABA induces cytosolic Ca^2+^ oscillation. | [43] | BL=1, RL=1, ABA=1, CO_2_=C_i_=1 | In the model cytosolic Ca^2+^ increases, peaks, then it decreases in response to ABA; there is no consecutive increase. | PC |
| ROS inhibits white light-induced stomatal opening. | [S3] | White light without ROS: BL=1, RL=1, ABA=0, CO_2_=C_i_=1 | Stomatal opening=11.28 | C |
|  |  | White light with ROS: BL=1, RL=1, ABA=0, CO_2_=C_i_=1, ROS is kept 1 | Stomatal opening=8.92 |  |
| ROS inhibits blue light-induced stomatal opening. | [17] | Blue light without ROS: BL=1, RL=0, ABA=0, CO_2_=C_i_=1 | Stomatal opening=4.15 | C |
|  |  | Blue light with ROS: BL=1, RL=0, ABA=0, CO_2_=C_i_=1, ROS is kept 1 | Stomatal opening=3.84 |  |
| NO donor SNP inhibits white light-induced stomatal opening. | [S3] | White light without NO donor SNP: BL=1, RL=1, ABA=0, CO_2_=C_i_=1 | Stomatal opening=11.28 | C |
|  |  | White light with NO donor SNP: BL=1, RL=1, ABA=0, CO_2_=C_i_=1, NO is kept 1 | Stomatal opening=8.92 |  |
| NO donor SNP inhibits blue light-induced stomatal opening. | [17, S74] | Blue light without NO donor SNP: BL=1, RL=0, ABA=0, CO_2_=C_i_=1 | Stomatal opening=4.15 | C |
|  |  | Blue light with NO donor SNP: BL=1, RL=0, ABA=0, CO_2_=C_i_=1, NO is kept 1 | Stomatal opening=3.84 |  |
| NO donor SNP does not inhibit red light-induced stomatal opening. | [S74] | Red light without NO donor SNP: BL=0, RL=1, ABA=0, CO_2_=C_i_=1 | Stomatal opening=1 | C |
|  |  | Red light with NO donor SNP: BL=0, RL=1, ABA=0, CO_2_=C_i_=1, NO is kept 1 | Stomatal opening=1 |  |
| NO scavenger PTIO partially restores stomatal opening inhibited by ABA. | [S6] | White light without ABA: BL=1, RL=1, ABA=0, CO_2_=C_i_=1 | Stomatal opening=11.28 | C |
|  |  | White light with ABA: BL=1, RL=1, ABA=1, CO_2_=C_i_=1 | Stomatal opening=0 |  |
|  |  | White light with ABA and NO scavenger PTIO: BL=1, RL=1, ABA=1, CO_2_=C_i_=1, NO is kept 0 | Stomatal opening=5.18 |  |
| Anion channel blocker 9-AC reverses inhibition of white light-induced stomatal opening by ABA. | [41] | White light without ABA: BL=1, RL=1, ABA=0, CO_2_=C_i_=1 | Stomatal opening=11.28 | C |
|  |  | White light with ABA: BL=1, RL=1, ABA=1, CO_2_=C_i_=1 | Stomatal opening=0 |  |
|  |  | White light with ABA and anion channel blocked by 9-AC: BL=1, RL=1, ABA=1, CO_2_=C_i_=1, AnionCh is kept 0 | Stomatal opening=1.73 |  |
| ABA can activate anion efflux channels without the mediation of Ca^2+^. | [S73] | With the mediation of Ca^2+^: BL=1, RL=1, ABA=1, CO_2_=C_i_=1 | AnionCh=1.6 | C |
|  |  | Without the mediation of Ca^2+^: BL=1, RL=1, ABA=1, CO_2_=C_i_=1, [Ca^2+^]_c_ is kept 0 | AnionCh=1.6 |  |
| ABA inhibits blue light-induced H^+^-ATPase activity. | [44] | Without ABA: BL=1, RL=0, ABA=0, CO_2_=C_i_=1 | H^+^-ATPase_complex_=2 | C |
|  |  | With ABA: BL=1, RL=0, ABA=1, CO_2_=C_i_=1 | H^+^-ATPase_complex_=1 |  |
| ROS inhibits blue light-induced H^+^-ATPase activity. | [44] | Without ROS: BL=1, RL=0, ABA=0, CO_2_=C_i_=1 | H^+^-ATPase_complex_=2 | C |
|  |  | With ROS: BL=1, RL=0, ABA=0, CO_2_=C_i_=1, ROS is kept 1 | H^+^-ATPase_complex_=1.8 |  |
| ROS scavenger partially restores blue light-dependent H^+^-ATPase activity inhibited by ABA. | [44] | Blue light without ABA: BL=1, RL=0, ABA=0, CO_2_=C_i_=1 | H^+^-ATPase_complex_=2 | C |
|  |  | Blue light with ABA: BL=1, RL=0, ABA=1, CO_2_=C_i_=1 | H^+^-ATPase_complex_=1 |  |
|  |  | Blue light with ABA and ROS scavenger: BL=1, RL=0, ABA=1, CO_2_=C_i_=1, ROS is kept 0 | H^+^-ATPase_complex_=1.8 |  |
| PA inhibits white light-induced stomatal opening. | [42, S78] | White light: BL=1, RL=1, ABA=0, CO_2_=C_i_=1 | Stomatal opening=11.28 | C |
|  |  | White light with sustained PA: BL=1, RL=1, ABA=0, CO_2_=C_i_=1, PA is kept 1 | Stomatal opening=8.92 |  |
| PA inhibits blue light-induced stomatal opening. | [17] | Blue light: BL=1, RL=0, ABA=0, CO_2_=C_i_=1 | Stomatal opening=4.15 | C |
|  |  | Blue light with sustained PA: BL=1, RL=0, ABA=0, CO_2_=C_i_=1, PA is kept 1 | Stomatal opening=3.84 |  |
| PA does not inhibit red light-induced stomatal opening. | [17] | Red light: BL=0, RL=1, ABA=0, CO_2_=C_i_=1 | Stomatal opening=1 | C |
|  |  | Red light with sustained PA: BL=0, RL=1, ABA=0, CO_2_=C_i_=1, PA is kept 1 | Stomatal opening=1 |  |
| The inhibition (with 1-buOH) of PA production elicited by ABA partially prevents ABA's inhibition of white light-induced stomatal opening. | [42] | White light without ABA: BL=1, RL=1, ABA=0, CO_2_=C_i_=1 | Stomatal opening=11.28 | C |
|  |  | White light with ABA: BL=1, RL=1, ABA=1, CO_2_=C_i_=1 | Stomatal opening=0 |  |
|  |  | White light with ABA and PA inhibitor 1-buOH: BL=1, RL=1, ABA=1, CO_2_=C_i_=1, PA is kept 0 | Stomatal opening=6.9 |  |
| OST1 knockout mutation does not affect light-induced stomatal opening. | [18, S30] | Wild type under light: BL=1, RL=1, ABA=0, CO_2_=C_i_=1 | Stomatal opening=11.28 | C |
|  |  | OST1 knockout mutant under light: BL=1, RL=1, ABA=0, CO_2_=C_i_=1, OST1 is kept 0 | Stomatal opening=11.28 |  |
| OST1 knockout mutation disrupts ABA's inhibition of white light-induced stomatal opening. | [18, S30] | Wild type under white light without ABA: BL=1, RL=1, ABA=0, CO_2_=C_i_=1 | Stomatal opening=11.28 | C |
|  |  | Wild type under white light with ABA: BL=1, RL=1, ABA=1, CO_2_=C_i_=1 | Stomatal opening=0 |  |
|  |  | OST1 knockout mutant under white light with ABA: BL=1, RL=1, ABA=1, CO_2_=C_i_=1, OST1 is kept 0 | Stomatal opening=5.18 |  |
| ABA upregulates NADPH oxidases AtrbohD/F. | [S75] | Without ABA: BL=1, RL=1, ABA=0, CO_2_=C_i_=1 | AtrbohD/F=0 | C |
|  |  | With ABA: BL=1, RL=1, ABA=1, CO_2_=C_i_=1 | AtrbohD/F=1 |  |
| AtrbohD/F double knockout mutation impairs ROS production in response to ABA compared to wild type. | [S75] | Wild type with ABA: BL=1, RL=1, ABA=1, CO_2_=C_i_=1 | ROS=1 | C |
|  |  | AtrbohD/F double knockout mutant with ABA: BL=1, RL=1, ABA=1, CO_2_=C_i_=1, AtrbohD/F is kept 0 | ROS=0 |  |
| Inhibiting NADPH oxidase with DPI partially restores stomatal opening inhibited by ABA. | [76] | White light without ABA: BL=1, RL=1, ABA=0, CO_2_=C_i_=1 | Stomatal opening=11.28 | C |
|  |  | White light with ABA: BL=1, RL=1, ABA=1, CO_2_=C_i_=1 | Stomatal opening=0 |  |
|  |  | White light with ABA and NADPH oxidase inhibitor DPI: BL=1, RL=1, ABA=1, CO_2_=C_i_=1, AtrbohD/F is kept 0 | Stomatal opening=5.18 |  |
