# Supplementary material for: Multi-level Modeling of Light-Induced Stomatal Opening Offers New Insights into Its Regulation by Drought
Source: PLoS Comput Biol. 2014 Nov 13;10(11):e1003930. doi: 10.1371/journal.pcbi.1003930 (PMC4230748; doi:10.1371/journal.pcbi.1003930)
Supplement: Text S2 — Example of network construction. (DOCX) [file pcbi.1003930.s008.docx]

**Text S2: Example of network construction**

Here we use the pathway that starts from blue light and ends at the proton pump (H^+^-ATPase) located on the plasma membrane to illustrate the process of network construction based on the literature. This process can be done manually (as we did) or it can be done in a more formal and algorithmic manner using the software NET-SYNTHESIS [27]. We also illustrate how the use of NET-SYNTHESIS would yield the same conclusion as human inference (see Figure S2.1).

1. Blue light is perceived in guard cells by phototropins (phot1 and phot2) [14-16, S61]. This is represented by two separate edges that start from blue light and end at each phototropin. Blue light also promotes the reversible binding of a 14-3-3 protein (14-3-3 protein_phot1_) to phot1 but not to phot2 [102, 103]. The 14-3-3 protein-phot1 complex (phot1_complex_) mediates the propagation of the blue light signal to downstream components. Therefore, we include the phot1_complex_ node and two edges to represent the fact that the phot1_complex_ is formed from free phot1 and the 14-3-3 protein_phot1_. We infer that the phot1_complex_ is the starting node of any blue light effect mediated by phot1.

A similar inference can be made using NET-SYNTHESIS. First, we represent the statement “phot1_complex_ mediates the propagation of the blue light signal to downstream components”, choosing for concreteness the proton pump (H^+^-ATPase) as a downstream component, as the three-node indirect causal inference “phot1_complex_ 🡪 (Blue Light 🡪 H^+^-ATPase)”. Using the command “*Add pseudonodes*”, the software will interpret this inference by adding an unknown mediator (pseudo-node) between Blue Light and H^+^-ATPase, and adding an edge from phot1_complex_ to this pseudonode (Figure S2.1A). Then we add the other phot1-related edges in this paragraph: BL🡪 phot1; phot1🡪 phot1_complex_; 14-3-3 protein_phot1_ 🡪 phot1_complex_. Next we use the command “*Reduction*” to identify and delete edges that express causal effects that can also be explained by a path. The edge from Blue Light to the pseudonode is explained by the path from Blue Light to phot1, then phot1_complex,_ then the pseudonode. Thus this edge will be removed (Figure S2.1B). Next we can merge the pseudonode with its sole input (or its sole output) by using the command *“Collapse degree-2 pseudonodes”,* leading to an edge between phot1_complex_ and H^+^-ATPase (Figure S2.1C).


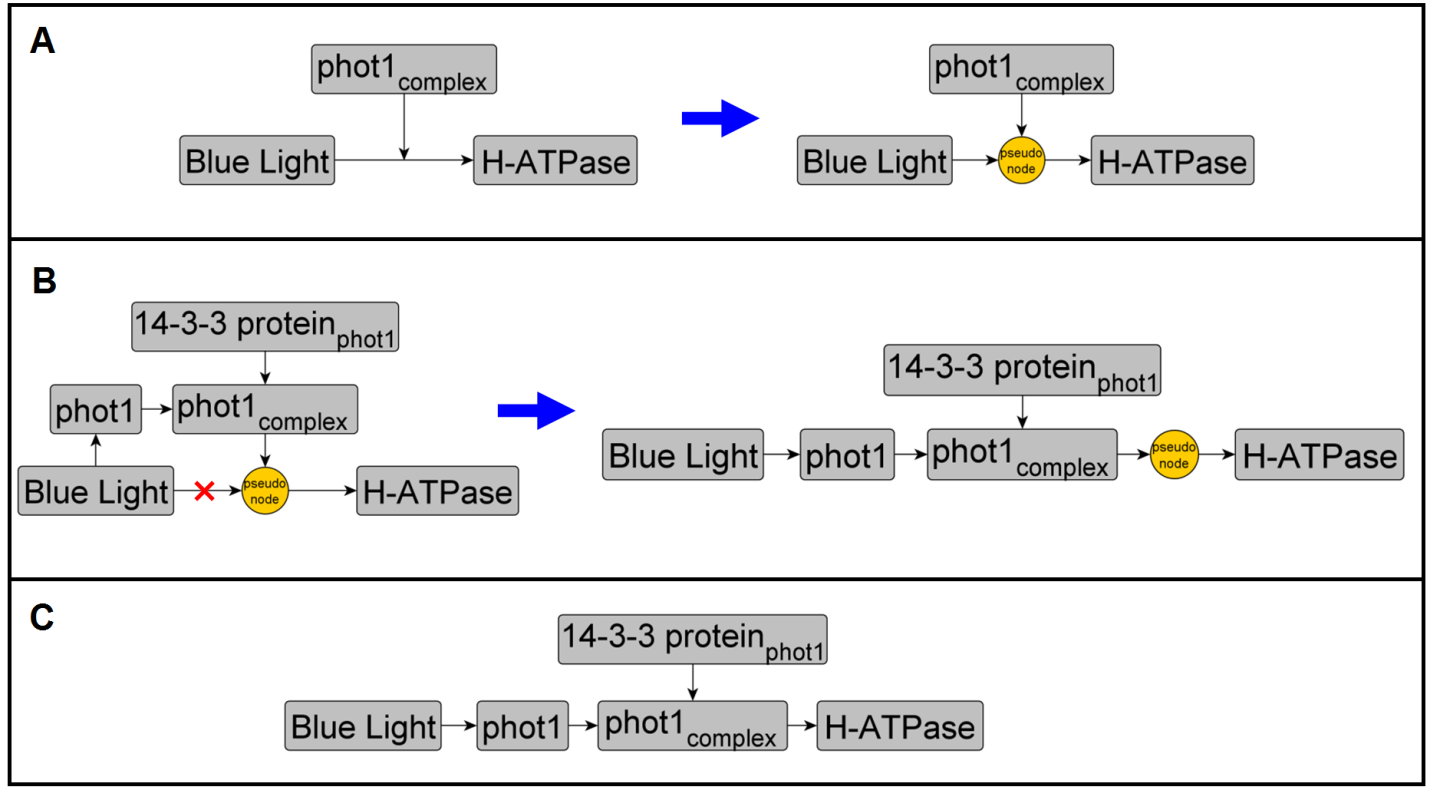


Figure S2.1 Illustration of the network inference and reduction process possible with the software NET-SYNTHESIS. (A) Representation of the mediation of the process "Blue Light 🡪 H^+^-ATPase" by the phot1_complex_ and the addition of the pseudonode. (B) The removal of a functionally redundant edge. (C) The network after the merging of the pseudonode with its sole input or sole output.

1. A type 1 protein phosphatase, PP1, has been implicated in the signaling between phototropins and the proton pump (H^+^-ATPase) [S13, S14]. Recent work identified the PP1 regulatory subunit 2-like protein1 (PRSL1) [S15], which stimulates the translocation of the PP1 catalytic subunit (PP1_c_) from the nucleus (PP1_cn_) to the cytosol (PP1_cc_), where it can participate in signal transduction. Impaired phosphorylation of the H^+^-ATPase in *Arabidopsis* *prsl1* mutants [S15] indicates that PP1_cc_ functions between blue light photoreception and H^+^-ATPase activation. This is represented by assuming that phot2 and the phot1_complex_ individually activate PRSL1, and PRSL1 activates the cytosol-localized PP1 catalytic subunit, PP1_cc_. Both PRSL1 and PP1_cn_ are connected by activating edges to PP1_cc_.
2. Direct phosphorylation of the plasma membrane H^+^-ATPase by an unspecified serine/threonine protein kinase (protein kinase) occurs in guard cells [36]. We add an edge from the protein kinase to the node H^+^-ATPase. Phosphorylation is a prerequisite for the binding of a 14-3-3 protein (14-3-3 protein_H-ATPase_) to the H^+^-ATPase [70]. This 14-3-3 protein_H-ATPase_, which utilizes a 14-3-3 protein distinct from the one in the phot1_complex_ [65, 102, S36], binds to the auto-inhibitory domain of the H^+^-ATPase, thus promoting H^+^-ATPase activity [65, 103, S36]. We define two nodes (H^+^-ATPase and H^+^-ATPase_complex_, respectively) to represent the states of the proton pump before and after the binding of this 14-3-3 protein_H-ATPase_. Both the unbound H^+^-ATPase and the 14-3-3 protein_H-ATPase_ are necessary to form the H^+^-ATPase_complex_. In addition, we include an edge between the node H^+^-ATPase and the 14-3-3 protein_H-ATPase_ to incorporate the requirement for phosphorylation of the proton pump prior to recruitment of the 14-3-3 protein_H-ATPase_. Since PP1 is responsible for transmitting the signal from the phototropins to the H^+^-ATPase, but it is not known what precedes the action of the protein kinase that phosphorylates the H^+^-ATPase, we hypothesize that PP1cc is upstream of the protein kinase.

The final reconstructed pathway is shown on Figure S2.2.


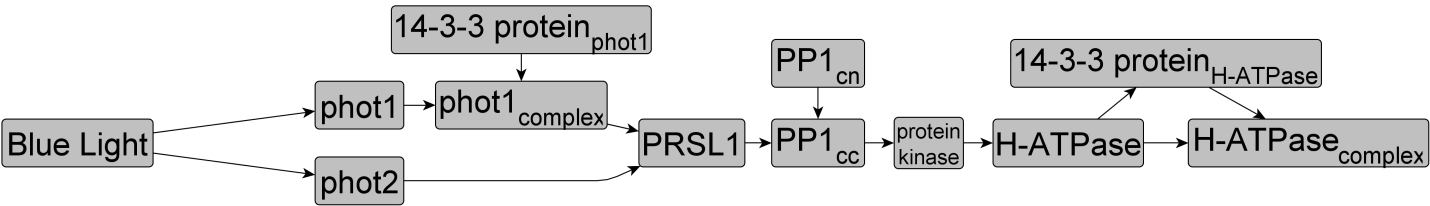


Figure S2.2 Example of organizing information extracted from the literature. The signaling pathway from blue light to the H^+^-ATPase is reconstructed.
