# Supplementary material for: Multi-level Modeling of Light-Induced Stomatal Opening Offers New Insights into Its Regulation by Drought
Source: PLoS Comput Biol. 2014 Nov 13;10(11):e1003930. doi: 10.1371/journal.pcbi.1003930 (PMC4230748; doi:10.1371/journal.pcbi.1003930)
Supplement: Text S3 — Description of the network. (DOCX) [file pcbi.1003930.s009.docx]

**Text S3: Description of the network**

A detailed description of the biology represented by the network in Figure 1 of the main text is as follows. phot1 and phot2 are accepted blue light-specific receptors [14, 15, S62] that function in guard cells [16, S61]. There are a number of experimentally confirmed functions for which both types of phototropins are upstream effectors, particularly increase in cytosolic Ca^2+^ concentration and H^+^ ATPase activation [4, 36, 50, 53, 63, 64].

Phospholipase C (PLC) is a downstream element specific to phot2 that can activate calcium release from intracellular stores (CaR). PLC can be activated by phot2 alone [S63], or by ABA together with [Ca^2+^]_c_ [43, S1, S64]_._ The latter renders a positive feedback mechanism for the self-sustainment of [Ca^2+^]_c_ elevation when ABA is present. In the absence of detailed information, we incorporate the Ca^2+^ decrease that follows the transient Ca^2+^ increase in response to blue light [53] by representing Ca^2+^ efflux from the cytosol by Ca^2+^-ATPases and Ca^2+^/H^+^ antiporters, incorporated as the node Ca^2+^-ATPase engaged in a negative feedback loop with [Ca^2+^]_c_. [Ca^2+^]_c_ is one of the most important secondary messengers in the guard cell system and it has numerous interactions with other elements. [Ca^2+^]_c_ activates calcium-dependent protein kinases (CDPKs), which activate channels mediating chloride and malate flux from the cytosol to the vacuole [54]. CDPKs, specifically CPK6 and CPK3, participate in a feedback mechanism that facilitates Ca^2+^ flux through inward calcium permeable channels (CaIC) under ABA signaling via production of reactive oxygen species (ROS) [S32]. The target of CDPKs is suggested to be upstream of ROS. Therefore, this is represented with a positive edge from CDPK to ROS. Elevation in [Ca^2+^]_c_ can activate slow vacuolar currents corresponding to cation release from vacuoles via the vacuolar two-pore channels [S10, S12], as well as vacuolar K^+^ selective currents corresponding to the tandem-pore K^+^ channel family [S11]. A high concentration of [Ca^2+^]_c_ also promotes inhibition of stomatal opening through the activation of anion efflux channels (AnionCh) and the inactivation of the H^+^-ATPase and K^+^ inward channels (K_in_) at the plasma membrane [55, 56, S40, S65].

The proton pump H^+^-ATPase plays a pivotal role in blue light signaling. Multiple experiments confirm that PP1 mediates the signaling between phototropins and the plasma membrane H^+^-ATPase [S13, S14]. The regulatory subunit of PP1, PRSL1, stimulates the translocation of PP1_c_ from the nucleus to the cytosol [S15]. This is represented by a cascade from 14-3-3 protein-bound phot1 and phot2 to PRSL1 and PRSL1 to PP1_cc_. A serine/threonine protein kinase directly phosphorylates the plasma membrane H^+^-ATPase [36, S35], followed by the binding of a 14-3-3 protein to the proton pump [65]. The proton pump is highly regulated: ATP powers proton pump activity, lysophosphatidylcholine (LPL) and free fatty acids (FFA) promote proton pump activity [S37], and high levels of [Ca^2+^]_c_ [56, S66] or high C_i_ concentrations decrease proton pump activity [86]. The H^+^-ATPase in turn regulates a number of downstream nodes. H^+^ exported from the cytosol by the H^+^-ATPase leads to apoplastic acidification (acid. of apoplast), and provides H^+^ for the activity of sucrose/H^+^ symporters [34, 83] such as AtSTP1 [84]. H^+^-ATPase activity is also able to enhance the catalysis of malate production by phospho*enol*pyruvate carboxylase (PEPC), likely by affecting cytosolic pH and PEPC phosphorylation status [S51]. Active H^+^-ATPase is the primary cause of a more negative plasma membrane voltage (PMV), i.e. driving plasma membrane hyperpolarization. The release of anions from guard cell cytosol to the apoplast through active anion efflux channels (AnionCh), or increased cytosolic concentration of positive ions (i.e. [K^+^]_c_, [Ca^2+^]_c_), on the contrary, increase the PMV, i.e. cause plasma membrane depolarization. A hyperpolarized plasma membrane is a condition for both the activation of K_in_ [S43] and the passive uptake of K^+^ from the apoplast to the cytosol via these channels, with a resultant increase in cytosolic K^+^ concentration ([K^+^]_c_) [37, 38]. A depolarized plasma membrane activates K^+^ efflux through voltage-regulated outwardly-rectifying K^+^ channels at the plasma membrane (K_out_) [7, S44, S67]. An increase in [K^+^]_c_ is electrically compensated for by an increase in the concentration of cytosolic anions: Cl^-^, NO_3_^-^, and malate^2-^, all of which are lost through efflux to the apoplast when the anion efflux channels are open. This is incorporated by making [K^+^]_c_ a requirement for [Cl^-^]_c_, [NO_3_^-^]_c_ and [malate^2-^]_c_, represented by three separate activation edges, and by having negative edges from AnionCh to [Cl^-^]_c_, [NO_3_^-^]_c_ and [malate^2-^]_c_, respectively. The uptake of NO_3_^-^ from the apoplast into the guard cell cytosol is mediated by the nitrate transporter AtNRT1.1 (CHL1) [S48]. (An)ions will be translocated from the cytosol into guard cell vacuoles, where they contribute to the vacuolar osmotic potential that eventually leads to stomatal opening.

Phospholipase A_2_β (PLA_2_β) catalyzes the hydrolysis of phospholipids into LPL and free fatty acids and has been shown to mediate white light-induced stomatal opening in *Arabidopsis* [S2]. This is represented by activation edges from blue and red light to PLA_2_β, and PLA_2_β to LPL and free fatty acids, respectively. We hypothesize that blue light activation of PLA_2_β is mediated by phototropins. Both LPL and free fatty acids promote H^+^-ATPase activity [S37], represented by two activation edges. Free fatty acids also enhance K_in_ and suppress K_out_ [S39]. Under white light, phosphatidylinositol 4,5-bisphosphate (PIP2) is translocated from the cytosol (PIP2_C_) to the plasma membrane (PIP2_PM_), where it inhibits the anion efflux channels [S16]. We assume that PIP2 translocation is activated by the blue component of white light; initial simulations using our dynamic model indicated that if red light would activate the translocation, the plasma membrane would stay hyperpolarized under red light after a pulse of blue light is given. This contradicts the observations that red light is ineffective in activating the H^+^-ATPase, the primary engine driving the plasma membrane hyperpolarization [18, 64, 79, 80]. In addition, blue light inhibits the anion efflux channels in a phototropin dependent manner [S57]. Synthesizing these two pieces of evidence, we hypothesize that blue light induces the translocation of PIP2 via phototropins, which then inhibits the anion efflux channels. This is represented in our graph by a cascade from blue light to phot1_complex_ and phot2, phot1_complex_ and phot2 to PIP2_PM_, and PIP2_PM_ inhibition of AnionCh.

Both blue and red light can be absorbed by photosynthetic pigments located in guard cell chloroplasts, thereby activating photophosphorylation [3, 40, S68, S69]. Atmospheric CO_2_ dissolved in the intercellular space is the source of CO_2_ for carbon fixation. Photosynthesis provides ATP and reducing power in the form of NADPH [S70], both of which are critical for subsequent metabolic processes. NADPH participates in the nitrate reductase (NIA1) - catalyzed reduction of nitrite to form nitric oxide (NO) [S3, S71]. ATP generated by photophosphorylation and by oxidative phosphorylation in mitochondria powers crucial activities such as the H^+^-ATPase [4, 80]. Sucrose, an important osmoticum under light, can be formed from intermediate carbon fixation products. Sucrose imported from the apoplast by sucrose/H^+^ symporters such as AtSTP1 [84] can also contribute to cytosolic sucrose accumulation. Malate is another highly regulated osmoticum. As a counterion for K^+^, malate can be formed from starch degradation as well as from intermediate carbon fixation products as a result of PEPC activity. In addition, malate can also be imported from the apoplast via the ABC transporter AtABCB14 [S58]. Cytosolic malate ([malate^2-^]_c_) can be transported into the vacuole ([malate^2-^]_v_) as needed for stomatal opening [54].

White light activates the translocation of the small G protein ROP2 to the plasma membrane, resulting in ROP2 activation [74], and an effector downstream of ROP2, ROP-interactive CRIB motif-containing protein 7 (RIC7), has been suggested to act as a slow attenuator of stomatal opening (acting on a timescale of an hour, compared to light-induced stomatal opening which occurs in seconds to minutes) that provides negative feedback which prevents excessive opening [74]. This is represented by activation edges from blue light and red light to ROP2, respectively, an activation edge from ROP2 to RIC7, and an inhibitory edge from RIC7 to stomatal opening. We hypothesize that phototropins mediate blue light activation of ROP2.

The drought hormone ABA activates K_out_ channels [S44, S45] and AnionCh [41, 45, S72, S73], represented by two direct activation edges. ABA also inhibits the H^+^-ATPase [44], and NO participates in this function [S74] possibly by promoting PA inhibition of PP1 [17]. Both ABA and NO have been indicated to induce PA production through phospholipase D (PLD) [42, S17, S18]. We incorporate this information as a cascade in which ABA and NO activate PLD, PLD activates PA, and PA inhibits PP1. PA also activates the small G protein ROP2 [S29]. Several ABA receptors have been identified, including the Pyrabactin Resistance/Pyrabactin Resistance-Like/Regulatory Components of ABA Receptor protein family [S19-S22], as well as G-protein coupled receptor–type G proteins [S24, S25]. The first family of ABA receptors, when bound by ABA, down-regulates protein phosphatase 2C (represented by ABI1) [S21, S22, S26]. We include a general node for all ABA receptors (including as-of-yet unidentified ones), as well as an activating edge from ABA to ABA receptors, and an inhibiting edge from ABA receptors to ABI1. PA induces generation of ROS through the activation of ROP2 [S29]. ABI1 and related PP2C phosphatases [S22, S26] inactivate OST1 [S27, S28]. Both ROP2 and OST1 upregulate NADPH oxidases (AtrbohD/F), which catalyze ROS production [S75]. ABI1 and related PP2C phosphatases suppress the anion efflux channels AnionCh [S54, S76]. ROS activate CaIC [75, 76] and inhibit K_out_ [S46], which makes ROS a major crosstalk agent between the ABA signaling cascade and the blue light response pathways. Reactive oxygen species promote NO generation through activating the enzyme NIA1 [S4], which is represented by activation edges from ROS to NIA1 and from NIA1 to NO. The protein nitric oxide-associated 1 (Atnoa1) is involved in NO accumulation [S5, S6] and ROS production [S33, S34], represented by two activation edges. ABA induces calcium release from intracellular stores (CaR) through PLC-catalyzed inositol-1,4,5-trisphosphate (InsP3) synthesis [43, S77], and this catalysis requires Ca^2+^ [S1]. Therefore, ABA and [Ca^2+^]_c_ are both activators of PLC. It has been documented that ABA inhibits malate synthesis activities in *Vicia faba* [S50], and that ABA induces malate breakdown by the TCA cycle and malate conversion back to starch [S49]; an inhibitory edge from mitochondria to [malate^2-^]_c_ and an activation edge from [malate^2-^]_c_ to starch, both activated by ABA, are implemented to represent this information. ABA also inhibits PEPC [S50].

Mesophyll cells can influence stomatal apertures by affecting the composition of the apoplastic fluid and the intercellular CO_2_ concentration, C_i_. Mesophyll cell photosynthesis lowers C_i_ and produces malate, which can be released to the apoplast. A high level of C_i_ activates the anion efflux channels, AnionCh [S42, S55, S56] and K_out_ channels and inhibits K_in_ channels [S42]. Malate exported to the apoplast can be imported by guard cells, resulting in an elevation of [malate^2-^]_c_ [S58]. A high level of apoplastic malate ([malate^2-^]_a_) promotes the activation of the anion efflux channels [S52].
