# Supplementary material for: Multi-level Modeling of Light-Induced Stomatal Opening Offers New Insights into Its Regulation by Drought
Source: PLoS Comput Biol. 2014 Nov 13;10(11):e1003930. doi: 10.1371/journal.pcbi.1003930 (PMC4230748; doi:10.1371/journal.pcbi.1003930)
Supplement: Text S4 — Evaluating the model's robustness. (DOCX) [file pcbi.1003930.s010.docx]

**Text S4: Evaluating the model's robustness**

**Determination of the number of replicate simulations**

Due to the absence of detailed knowledge on the relative reaction speeds in the system, we sample different timescales by implementing random order asynchronous update. The number of replicate simulations needs to be chosen so that the data generated are a statistically representative sample, and the process is computationally efficient (achievable in reasonable time) as well.

We investigated stomatal opening in response to a dual beam stimulus (blue and red light) as the basis of our selection (see Figure S4.1). We focus on time step 6, when the stomatal opening levels are around half-maximum and show a large variability among the simulations.

The standard error decreases with an increasing number of replicates, and becomes less than 0.1 (and less than 2% of the mean value) for more than 2000 runs. Pairwise comparison of the stomatal opening level distributions for two consecutive values of the number of simulations in Table S4.1 (e.g. 100 versus 200) indicates that the means of the distributions are statistically indistinguishable for 100 or more replicates. We concluded that using 2,000 replicate simulations represents a statistically reliable sampling and used this value for all analyses.

**
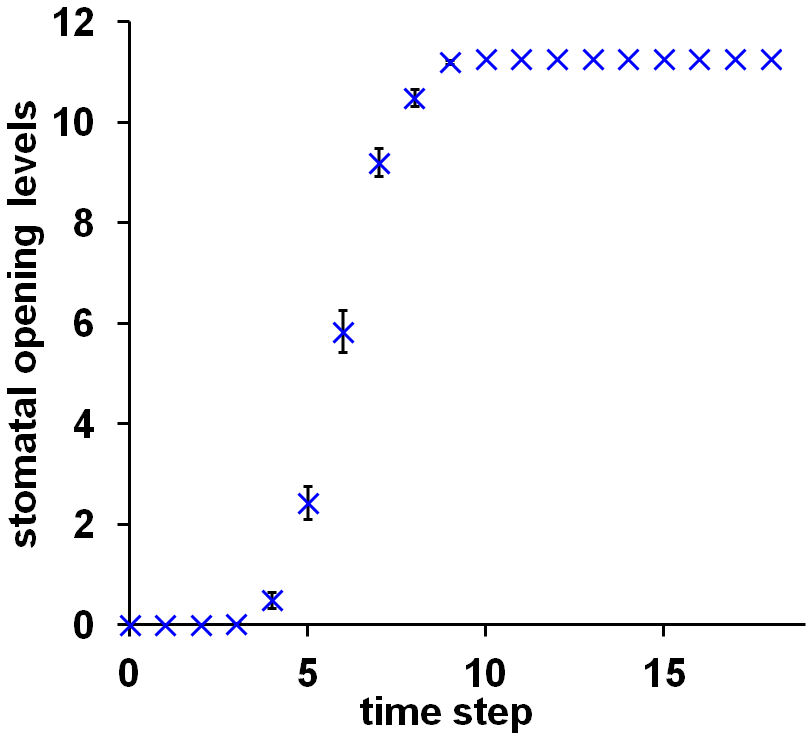
**

Figure S4.1 Stomatal opening in response to a dual beam stimulus for 100 replicate simulations. The error bars indicate standard errors.

| number of runs | 100 | 200 | 500 | 1000 | 2000 | 5000 | 10000 | 20000 | 30000 | 40000 | 50000 |
| --- | --- | --- | --- | --- | --- | --- | --- | --- | --- | --- | --- |
| mean stomatal opening | 5.85 | 5.72 | 5.74 | 5.69 | 5.77 | 5.80 | 5.84 | 5.77 | 5.74 | 5.78 | 5.75 |
| standard error | 0.42 | 0.30 | 0.19 | 0.13 | 0.09 | 0.06 | 0.04 | 0.03 | 0.02 | 0.02 | 0.02 |
| standard error as % of mean | 7.1 | 5.3 | 3.2 | 2.3 | 1.6 | 1.0 | 0.7 | 0.5 | 0.4 | 0.4 | 0.3 |

Table S4.1 Mean stomatal opening levels and the corresponding standard error of the mean at time step 6 for different numbers of replicate simulations. Standard error is shown both as absolute value and as a percentage of the mean stomatal opening level.

**Model sensitivity and uncertainty test**

Despite our efforts to incorporate the most up-to-date information, the knowledge that is represented by our model could potentially be incomplete, leading to uncertainty in the update rules for certain nodes. In addition, new biological evidence is constantly emerging, which could result in the modification of node update rules in our model as well.

In order to test our model’s robustness against uncertainty and stochasticity in the update rules, we carried out a comprehensive 'fault test' of our model. In this test we allow the state of either a single node (sensitivity test) or of all internal nodes in our model (uncertainty test) to update randomly with probability p. In the sensitivity test we represent the effects of regulatory stochasticity or of potential unknown regulators of a chosen node by doing the following: with probability *p*, the state of the node is randomly chosen to be any state that is available to that node and with probability 1-*p* the state follows the original transition rule. The probability *p* ranges from 0 to 1, with *p*=0 representing the original model with no perturbation to the node and *p*=1 representing a completely random update of the node.

We showcase the results of the sensitivity analysis for two representative nodes: the proton pump H^+^-ATPase (Figure 4.1A, 4.1B) and the photoreceptor phot2 (Figure 4.1C, 4.1D). In both cases the chosen node is set to update to a randomly chosen value with probability *p*=0.5 and follows its original rule otherwise. Since the perturbation is limited to a single node, a relatively high perturbation rate (*p*=0.5) was used. Panels A and C show the stomatal opening level for *p*=0 (the original model), while panels B and D show the opening level for *p*=0.5. The continuous lines reflect the mean of 2,000 simulations using dual beam without ABA under moderate CO_2_ condition as inputs (BL=1, RL=1, ABA=0, CO_2_=C_i_=1), and the error bars represent standard deviation.


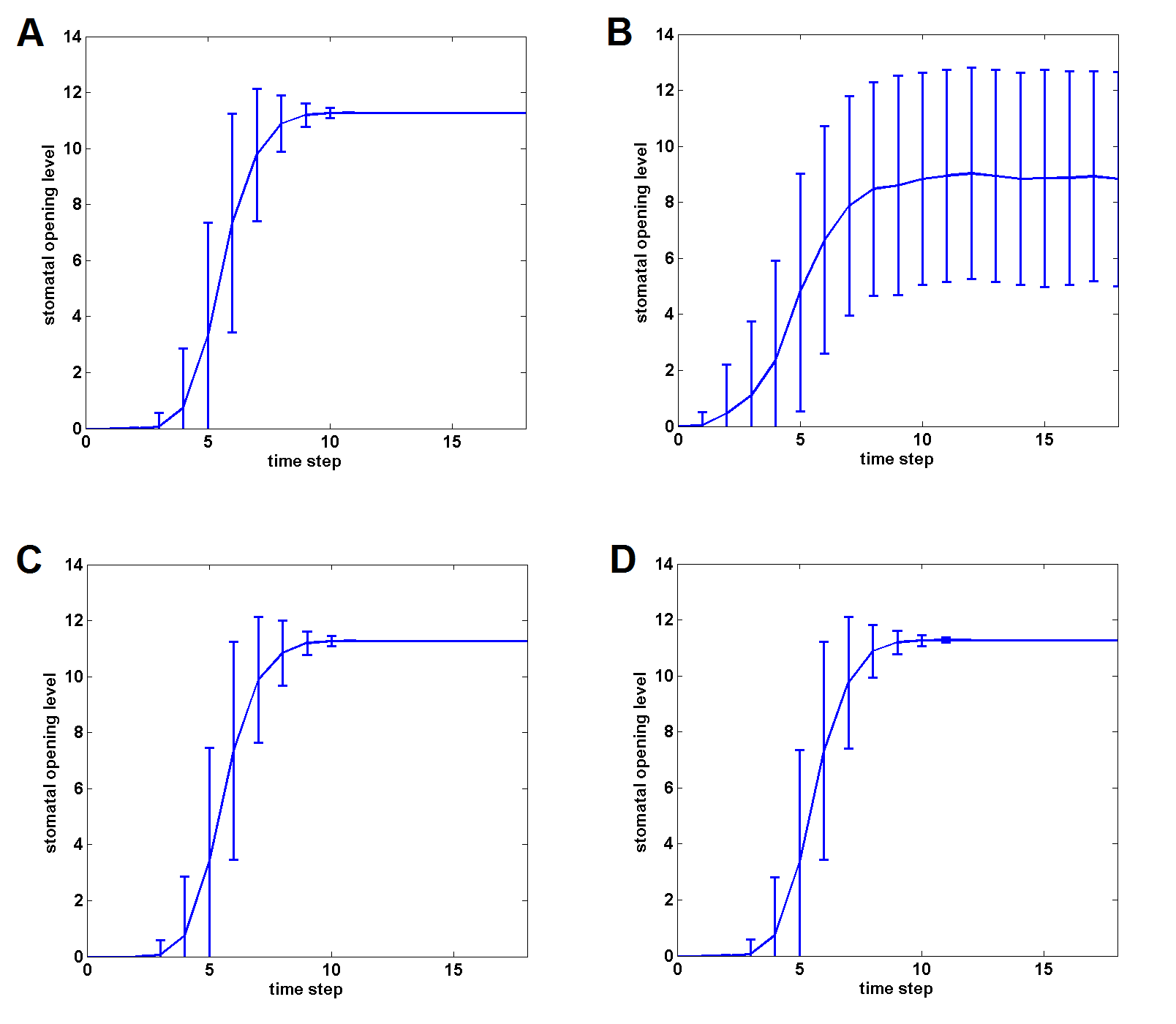


Figure 4.1. Sensitivity test of the system. (A) Time course of the stomatal opening level for *p*=0 (no perturbation to the system, used as control). (B) Time course of the stomatal opening level when the state of the node H^+^-ATPase_complex_ is randomized with probability *p*=0.5. (C) Time course of the stomatal opening level for *p*=0 (no perturbation to the system, used as control). (D) Time course of the stomatal opening level when the state of node phot2 is randomized with probability *p*=0.5.

We found that while the system is sensitive to uncertainty in the update rule of nodes like the H^+^-ATPase, it is not sensitive to uncertainly in the update rule of other nodes like phot2. The single node knockout simulations described in Table 5 and Table S3 offer a good estimation of the upper bound of the sensitivity of the system’s behavior to uncertainty in single nodes’ regulation, since randomly choosing an available state is still better than being completely knocked out. As an example, blue light-induced stomatal opening in moderate CO_2_ containing air is not completely inhibited by any single knockout mutant. Thus randomizing the rule of any node should not induce complete inhibition either. Under the same condition, 68.8% of all single knockout cases show wild type behavior. Randomizing the rule of a node, which does less damage to the system than knocking out the same node, will thus lead to wild type opening levels in 68.8% or more of cases.

In a general effort to take into account potentially false positive or false negative edges in our model, we also carried out the same rule randomization as described above, but this time the rules for all internal nodes (those that are not signals or the output) are randomized at every time step. Since the whole system is under perturbation, we can expect that even a low probability of randomization leads to significant change in the model’s behavior. As an example, a probability of *p*=0.1 is used for this test. This analysis offers an upper bound to cases of missing knowledge, since the rules for all internal nodes are repeatedly randomized at every time step. In this case as well we used the condition of dual beam without ABA under moderate CO_2_ (BL=1, RL=1, ABA=0, CO_2_=C_i_=1), and show the mean stomatal opening level on Figure 4.2; standard deviations from 2000 runs provide error bars.


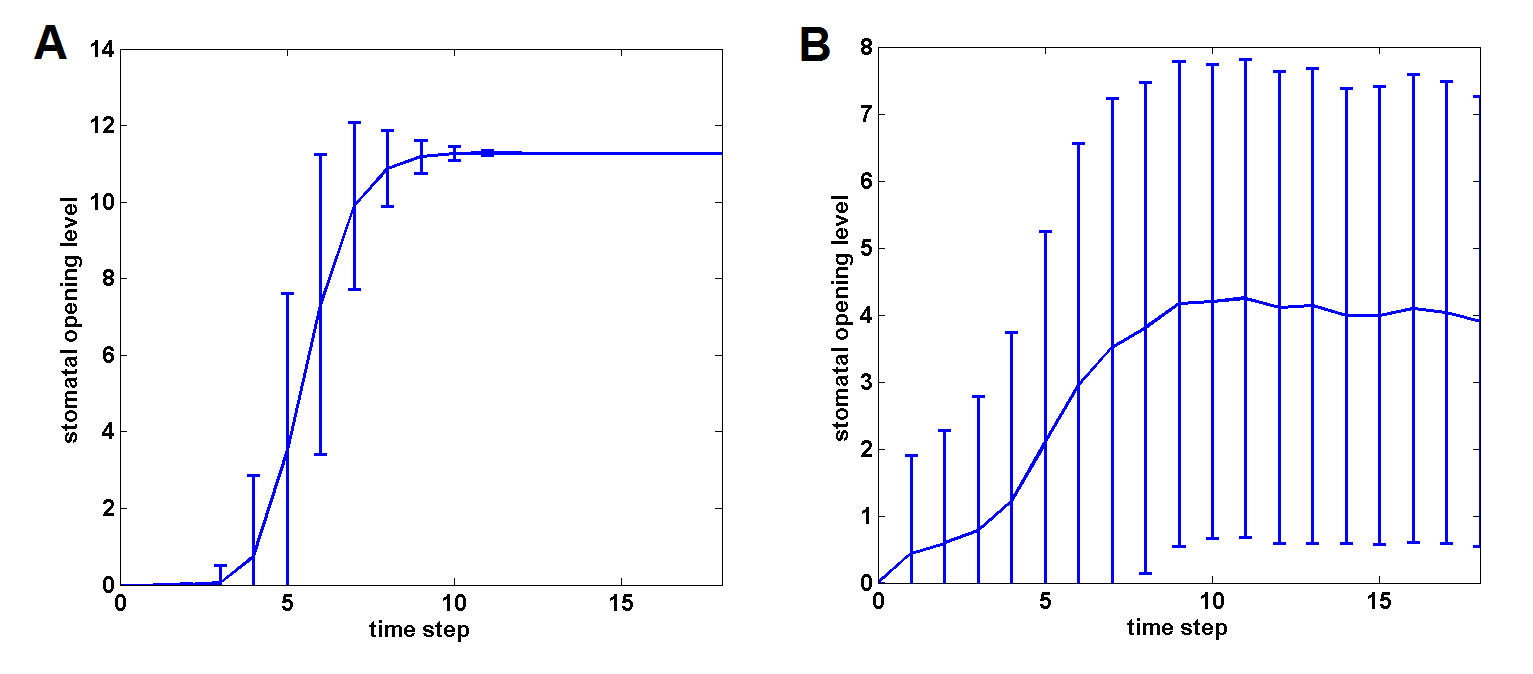


Figure 4.2. Uncertainty test of the system. (A) Time course of the stomatal opening level for *p*=0 (no perturbation to the system, used as control). (B) Time course of the stomatal opening level when the states of all internal nodes are randomized with probability *p*=0.1 at each time step.

We note that randomizing the node states with *p*=0.1 for *all* nodes is a severe perturbation to the system. The fact that we still get stomatal opening establishes the robustness of the model. Combined with the sensitivity analysis, we conclude that the model strikes a good balance between being sensitive to stochasticity and uncertainty in the regulation of a few select nodes and being robust to stochasticity and uncertainly in the regulation of most nodes.
