# Supplementary material for: Multi-level Modeling of Light-Induced Stomatal Opening Offers New Insights into Its Regulation by Drought
Source: PLoS Comput Biol. 2014 Nov 13;10(11):e1003930. doi: 10.1371/journal.pcbi.1003930 (PMC4230748; doi:10.1371/journal.pcbi.1003930)
Supplement: Text S5 — Pseudo-code for stomatal opening simulations. (DOCX) [file pcbi.1003930.s011.docx]

**Text S5: Pseudo code for stomatal opening simulations**

Here we present the algorithm to simulate the temporal trajectory of our model. The actual code, written in MATLAB, is available upon request. All nodes have two or more states (levels), and the update rules of the nodes contain Boolean and/or algebraic operations. These rules and the initial states of the nodes are described in Text S1.

**Input:** *Nodes* = {Node1; Node2; …; Node(N-1); Stomatal_Opening}

*Rules* = {Rule_of_Node1; Rule_of_Node2; …; Rule_of_Node_(N-1); Rule_of_Stomatal_Opening}

*N* = Number_of_Nodes

*NoT* = Number_of_Timesteps

*NoS* = Number_of_Simulations

**Output:** An *N* by *(NoT+1)* matrix that indicates the state of each node at every time step for a single simulation. For *NoS* number of runs, the result is an *N* by *(NoT+1)* matrix that shows the average state of each node at every time step; the average is over the *NoS* simulations. The first column of the matrix is the initial state of the system, or the state of every node at the 0^th^ time step. We require the last node to be *Stomatal_Opening*, and that *Stomatal_Opening* is updated last at every time step.

**Implementation of two representative update rules:**

Example 1:

**H^+^-ATPase_complex_^*^ = [(FFA *Or* LPL) *And Not* ([Ca^2+^]_c_ = 2) *And* 14-3-3 protein_H+-ATPase_] × H^+^-ATPase × ATP**

The states (levels) of the input nodes, taken from the most up-to-date state vector of the system:

FFA = 1; LPL = 1; [Ca^2+^]_c_ = 1; 14-3-3 protein_H+-ATPase_ = 1; H^+^-ATPase = 2; ATP = 3.

Since [Ca^2+^]_c_ = 1, the Boolean determinant ([Ca^2+^]_c_ = 2 ) in the rule yields FALSE, or value 0.

The Boolean clause of the rule then yields:

[(FFA *Or* LPL) *And Not* ([Ca^2+^]_c_ = 2) *And* 14-3-3 protein_H+-ATPase_] = [(1 *Or* 1) *And Not* 0 *And* 1] = 1

The level of H^+^-ATPase_complex_ is then 1×2×3 = 6.

Example 2:

| **PP1_cn_** | **PRSL1** | **PA** | **PP1_cc_^*^** |
| --- | --- | --- | --- |
| **0** | ***a*** | ***b*** | **0** |
| **1** | **0** | **0** | **2** |
|  |  | **1** | **1.5** |
|  |  | **2** | **1** |
|  | **1** | **0** | **4** |
|  |  | **1** | **3.5** |
|  |  | **2** | **3** |

Our implementation of the truth table:

1 if PP1_cn_ equals 0

2 PP1_cc_ = 0, regardless of the value of PRSL1, PA

3 if PP1_cn_ equals 1

4 if PRSL1 equals 0

5 PP1_cc_ = 2 – PA/2

6 if PRSL1 equals 1

7 PP1_cc_ = 4 – PA/2

The states of the input nodes, taken from the most up-to-date state vector of the system:

PP1_cn_ = 1; PRSL1 = 1; PA = 1.

Since PP1_cn_ = 1, PRSL1 = 1, the equation PP1_cc_ = 4 – PA/2 is used. The resulting PP1_cc_ value is 3.5. The corresponding input entries in the truth table are shaded blue, and the output entry is shaded red.

**Simulation procedure:**

**Initialization**

*Initialstate_Vector* = [Initialstate_of_Node1; Initialstate_of_Node2; …; Initialstate_of_Node(N-1); Initialstate_of_Stomatal_Opening]

*Simulation_Result* is initialized as an *N* by *(NoT+1)* matrix, with its first column being *Initialstate_Vector*.

**State updates**

**For a single simulation**

1 *State_Vector* = *Initialstate_Vector*

2 **for** j = 1 to *NoT*

3 generate a permutation of (N-1), stored in the vector *random_sequence*

4 **for** k = 1 to *N*-1 in *random_sequence*

5 calculate the updated state of the *k*^th^ node using its rule and the states of

its input nodes extracted from the vector *State_Vector*

6 store the updated state of the *k*^th^ node back into *State_Vector*

7 **end**

8 calculate the state of *Stomatal_Opening* using its rule and the states of its input

nodes extracted from *State_Vector*

9 store the updated state of *Stomatal_Opening* into *State_Vector*

10 store *State_Vector* to the (i+1)^th^ column of *Simulation_Result*

11 **end**

**For multiple simulations (*NoS* simulations)**

1 **for** i = 1 to *NoS*

2 **Run a single simulation.** For line 10, instead of simply storing, we now **add**

*State_Vector* to the (i+1)^th^ column of *Simulation_Result*

3 **end**

4 *Simulation_Result* = *Simulation_Result*/*NoS*
