# Supplementary material for: Multi-level Modeling of Light-Induced Stomatal Opening Offers New Insights into Its Regulation by Drought
Source: PLoS Comput Biol. 2014 Nov 13;10(11):e1003930. doi: 10.1371/journal.pcbi.1003930 (PMC4230748; doi:10.1371/journal.pcbi.1003930)
Supplement: Text S6 — List of references that appear in the supporting information files. (DOCX) [file pcbi.1003930.s012.docx]

**Text S6: List of references that appear in the supporting information files**

S1. Otterhag L, Sommarin M, Pical C (2001) N-terminal EF-hand-like domain is required for phosphoinositide-specific phospholipase C activity in *Arabidopsis thaliana*. FEBS Letters 497: 165-170.

S2. Seo J, Lee HY, Choi H, Choi Y, Lee Y, et al. (2008) Phospholipase A_2_beta mediates light-induced stomatal opening in *Arabidopsis*. Journal of Experimental Botany 59: 3587-3594.

S3. Desikan R, Griffiths R, Hancock J, Neill S (2002) A new role for an old enzyme: nitrate reductase-mediated nitric oxide generation is required for abscisic acid-induced stomatal closure in *Arabidopsis thaliana*. Proceedings of the National Academy of Sciences of the United States of America 99: 16314-16318.

S4. Bright J, Desikan R, Hancock JT, Weir IS, Neill SJ (2006) ABA-induced NO generation and stomatal closure in *Arabidopsis* are dependent on H_2_O_2_ synthesis. The Plant Journal 45: 113-122.

S5. Guo FQ, Okamoto M, Crawford NM (2003) Identification of a plant nitric oxide synthase gene involved in hormonal signaling. Science 302: 100-103.

S6. Neill S, Barros R, Bright J, Desikan R, Hancock J, et al. (2008) Nitric oxide, stomatal closure, and abiotic stress. Journal of Experimental Botany 59: 165-176.

S7. Lu D, Zhang X, Jiang J, An GY, Zhang LR, et al. (2005) NO may function in the downstream of H_2_O_2_ in ABA-induced stomatal closure in *Vicia faba* L. Journal of Plant Physiology and Molecular Biology 31: 62-70.

S8. Sanders D, Pelloux J, Brownlee C, Harper JF (2002) Calcium at the crossroads of signaling. The Plant Cell 14 Suppl: S401-417.

S9. Grabov A, Blatt MR (1998) Membrane voltage initiates Ca^2+^ waves and potentiates Ca^2+^ increases with abscisic acid in stomatal guard cells. Proceedings of the National Academy of Sciences of the United States of America 95: 4778-4783.

S10. Peiter E, Maathuis FJ, Mills LN, Knight H, Pelloux J, et al. (2005) The vacuolar Ca^2+^-activated channel TPC1 regulates germination and stomatal movement. Nature 434: 404-408.

S11. Latz A, Becker D, Hekman M, Muller T, Beyhl D, et al. (2007) TPK1, a Ca^2+^-regulated *Arabidopsis* vacuole two-pore K^+^ channel is activated by 14-3-3 proteins. The Plant Journal 52: 449-459.

S12. Schulze C, Sticht H, Meyerhoff P, Dietrich P (2011) Differential contribution of EF-hands to the Ca^2+^-dependent activation in the plant two-pore channel TPC1. The Plant Journal 68: 424-432.

S13. Kinoshita T, Shimazaki K (1997) Involvement of calyculin A- and okadaic acid-sensitive protein phosphatase in the blue light response of stomatal guard cells. Plant and Cell Physiology 38: 1281-1286.

S14. Takemiya A, Kinoshita T, Asanuma M, Shimazaki K (2006) Protein phosphatase 1 positively regulates stomatal opening in response to blue light in *Vicia faba*. Proceedings of the National Academy of Sciences of the United States of America 103: 13549-13554.

S15. Takemiya A, Yamauchi S, Yano T, Ariyoshi C, Shimazaki K (2013) Identification of a regulatory subunit of protein phosphatase 1 which mediates blue light signaling for stomatal opening. Plant and Cell Physiology 54: 24-35.

S16. Lee Y, Kim YW, Jeon BW, Park KY, Suh SJ, et al. (2007) Phosphatidylinositol 4,5-bisphosphate is important for stomatal opening. The Plant Journal 52: 803-816.

S17. Distefano AM, Garcia-Mata C, Lamattina L, Laxalt AM (2008) Nitric oxide-induced phosphatidic acid accumulation: a role for phospholipases C and D in stomatal closure. Plant, Cell & Environment 31: 187-194.

S18. Wang X (2005) Regulatory functions of phospholipase D and phosphatidic acid in plant growth, development, and stress responses. Plant Physiology 139: 566-573.

S19. Ma Y, Szostkiewicz I, Korte A, Moes D, Yang Y, et al. (2009) Regulators of PP2C phosphatase activity function as abscisic acid sensors. Science 324: 1064-1068.

S20. Nishimura N, Sarkeshik A, Nito K, Park SY, Wang A, et al. (2010) PYR/PYL/RCAR family members are major in-vivo ABI1 protein phosphatase 2C-interacting proteins in *Arabidopsis*. The Plant Journal 61: 290-299.

S21. Park SY, Fung P, Nishimura N, Jensen DR, Fujii H, et al. (2009) Abscisic acid inhibits type 2C protein phosphatases via the PYR/PYL family of START proteins. Science 324: 1068-1071.

S22. Santiago J, Rodrigues A, Saez A, Rubio S, Antoni R, et al. (2009) Modulation of drought resistance by the abscisic acid receptor PYL5 through inhibition of clade A PP2Cs. The Plant Journal 60: 575-588.

S23. Yin Y, Adachi Y, Ye W, Hayashi M, Nakamura Y, et al. (2013) Difference in abscisic acid perception mechanisms between closure induction and opening inhibition of stomata. Plant Physiology 163: 600-610.

S24. Pandey S, Nelson DC, Assmann SM (2009) Two novel GPCR-type G proteins are abscisic acid receptors in *Arabidopsis*. Cell 136: 136-148.

S25. Kharenko OA, Choudhary P, Loewen MC (2013) Abscisic acid binds to recombinant *Arabidopsis* *thaliana* G-protein coupled receptor-type G-protein 1 in *Sacaromycese cerevisiae* and *in vitro*. Plant Physiology and Biochemistry: 68: 32-36.

S26. Rubio S, Rodrigues A, Saez A, Dizon MB, Galle A, et al. (2009) Triple loss of function of protein phosphatases type 2C leads to partial constitutive response to endogenous abscisic acid. Plant Physiology 150: 1345-1355.

S27. Umezawa T, Sugiyama N, Mizoguchi M, Hayashi S, Myouga F, et al. (2009) Type 2C protein phosphatases directly regulate abscisic acid-activated protein kinases in *Arabidopsis*. Proceedings of the National Academy of Sciences of the United States of America 106: 17588-17593.

S28. Vlad F, Rubio S, Rodrigues A, Sirichandra C, Belin C, et al. (2009) Protein phosphatases 2C regulate the activation of the Snf1-related kinase OST1 by abscisic acid in *Arabidopsis*. The Plant Cell 21: 3170-3184.

S29. Park J, Gu Y, Lee Y, Yang Z, Lee Y (2004) Phosphatidic acid induces leaf cell death in *Arabidopsis* by activating the Rho-related small G protein GTPase-mediated pathway of reactive oxygen species generation. Plant Physiology 134: 129-136.

S30. Mustilli AC, Merlot S, Vavasseur A, Fenzi F, Giraudat J (2002) *Arabidopsis* OST1 protein kinase mediates the regulation of stomatal aperture by abscisic acid and acts upstream of reactive oxygen species production. The Plant Cell 14: 3089-3099.

S31. Zhang Y, Zhu H, Zhang Q, Li M, Yan M, et al. (2009) Phospholipase D alpha1 and phosphatidic acid regulate NADPH oxidase activity and production of reactive oxygen species in ABA-mediated stomatal closure in *Arabidopsis*. The Plant Cell 21: 2357-2377.

S32. Mori IC, Murata Y, Yang Y, Munemasa S, Wang YF, et al. (2006) CDPKs CPK6 and CPK3 function in ABA regulation of guard cell S-type anion- and Ca^2+^-permeable channels and stomatal closure. PLoS Biology 4: e327.

S33. Moreau M, Lee GI, Wang Y, Crane BR, Klessig DF (2008) AtNOS/AtNOA1 is a functional *Arabidopsis* *thaliana* cGTPase and not a nitric-oxide synthase. The Journal of Biological Chemistry 283: 32957-32967.

S34. Guo FQ, Crawford NM (2005) *Arabidopsis* nitric oxide synthase1 is targeted to mitochondria and protects against oxidative damage and dark-induced senescence. The Plant Cell 17: 3436-3450.

S35. Svennelid F, Olsson A, Piotrowski M, Rosenquist M, Ottman C, et al. (1999) Phosphorylation of Thr-948 at the C terminus of the plasma membrane H^+^-ATPase creates a binding site for the regulatory 14-3-3 protein. The Plant Cell 11: 2379-2391.

S36. Emi T, Kinoshita T, Shimazaki K (2001) Specific binding of vf14-3-3a isoform to the plasma membrane H^+^-ATPase in response to blue light and fusicoccin in guard cells of broad bean. Plant Physiology 125: 1115-1125.

S37. Palmgren MG, Sommarin M, Ulvskov P, Jorgensen PL (1988) Modulation of plasma membrane H^+^-ATPase from oat roots by lysophosphatidylcholine, free fatty acids and phospholipase A_2_. Physiologia Plantarum 74: 11-20.

S38. Shimazaki K, Tominaga M, Shigenaga A (1997) Inhibition of the stomatal blue light response by verapamil at high concentration. Plant and Cell Physiology 38: 747-750.

S39. Lee Y, Lee HJ, Crain RC, Lee A, Korn SJ (1994) Polyunsaturated fatty acids modulate stomatal aperture and two distinct K^+^ channel currents in guard cells. Cellular Signalling 6: 181-186.

S40. Romano LA, Jacob T, Gilroy S, Assmann SM (2000) Increases in cytosolic Ca^2+^ are not required for abscisic acid-inhibition of inward K^+^ currents in guard cells of *Vicia faba* L. Planta 211: 209-217.

S41. Fairley-Grenot KA, Assmann SM (1992) Whole-cell K^+^ current across the plasma membrane of guard cells from a grass: *Zea mays*. Planta 186: 282-293.

S42. Brearley J, Venis MA, Blatt MR (1997) The effect of elevated CO_2_ concentrations on K^+^ and anion channels of *Vicia faba* L. guard cells. Planta 203: 145-155.

S43. Schroeder JI, Raschke K, Neher E (1987) Voltage dependence of K channels in guard-cell protoplasts. Proceedings of the National Academy of Sciences of the United States of America 84: 4108-4112.

S44. Miedema H, Assmann SM (1996) A membrane-delimited effect of internal pH on the K^+^ outward rectifier of *Vicia faba* guard cells. The Journal of Membrane Biology 154: 227-237.

S45. Blatt MR, Armstrong F (1993) Potassium channels of stomatal guard cells: Abscisic acid-evoked control of the outward rectifier mediated by cytoplasmic pH. Planta 191: 330-342.

S46. Kohler B, Hills A, Blatt MR (2003) Control of guard cell ion channels by hydrogen peroxide and abscisic acid indicates their action through alternate signaling pathways. Plant Physiology 131: 385-388.

S47. Sokolovski S, Blatt MR (2004) Nitric oxide block of outward-rectifying K^+^ channels indicates direct control by protein nitrosylation in guard cells. Plant Physiology 136: 4275-4284.

S48. Guo FQ, Young J, Crawford NM (2003) The nitrate transporter AtNRT1.1 (CHL1) functions in stomatal opening and contributes to drought susceptibility in *Arabidopsis*. The Plant Cell 15: 107-117.

S49. Dittrich P, Raschke K (1977) Malate metabolism in isolated epidermis of *Commelina communis* L. in relation to stomatal functioning. Planta 134: 77-81.

S50. Du Z, Aghoram K, Outlaw WH, Jr. (1997) *In vivo* phosphorylation of phosphoenolpyruvate carboxylase in guard cells of *Vicia faba* L. is enhanced by fusicoccin and suppressed by abscisic acid. Archives of Biochemistry and Biophysics 337: 345-350.

S51. Meinhard M, Schnabl H (2001) Fusicoccin- and light-induced activation and *in vivo* phosphorylation of phosphoenolpyruvate carboxylase in *Vicia* guard cell protoplasts. Plant Science 160: 635-646.

S52. Hedrich R, Marten I (1993) Malate-induced feedback regulation of plasma membrane anion channels could provide a CO_2_ sensor to guard cells. The EMBO Journal 12: 897-901.

S53. Wang XQ, Ullah H, Jones AM, Assmann SM (2001) G protein regulation of ion channels and abscisic acid signaling in *Arabidopsis* guard cells. Science 292: 2070-2072.

S54. Pei ZM, Kuchitsu K, Ward JM, Schwarz M, Schroeder JI (1997) Differential abscisic acid regulation of guard cell slow anion channels in *Arabidopsis* wild-type and *abi1* and *abi2* mutants. The Plant Cell 9: 409-423.

S55. Roelfsema MR, Hanstein S, Felle HH, Hedrich R (2002) CO_2_ provides an intermediate link in the red light response of guard cells. The Plant Journal 32: 65-75.

S56. Hanstein SM, Felle HH (2002) CO_2_-triggered chloride release from guard cells in intact fava bean leaves. Kinetics of the onset of stomatal closure. Plant Physiology 130: 940-950.

S57. Marten H, Hedrich R, Roelfsema MR (2007) Blue light inhibits guard cell plasma membrane anion channels in a phototropin-dependent manner. The Plant Journal 50: 29-39.

S58. Lee M, Choi Y, Burla B, Kim YY, Jeon B, et al. (2008) The ABC transporter AtABCB14 is a malate importer and modulates stomatal response to CO_2_. Nature Cell Biology 10: 1217-1223.

S59. Raschke K, Schnabl H (1978) Availability of chloride affects the balance between potassium chloride and potassium malate in guard cells of *Vicia faba* L. Plant Physiology 62: 84-87.

S60. Mohr H, Schopfer P (1995) Plant Physiology. Berlin: Springer-Verlag, 629 p.

S61. Boccalandro HE, Giordano CV, Ploschuk EL, Piccoli PN, Bottini R, et al. (2012) Phototropins but not cryptochromes mediate the blue light-specific promotion of stomatal conductance, while both enhance photosynthesis and transpiration under full sunlight. Plant Physiology 158: 1475-1484.

S62. Jarillo JA, Ahmad M, Cashmore AR (1998) NPL1: A second member of the NPH1 serine/threonine protein kinase family of *Arabidopsis*. Plant Physiology 117: 719.

S63. Harada A, Sakai T, Okada K (2003) Phot1 and phot2 mediate blue light-induced transient increases in cytosolic Ca^2+^ differently in *Arabidopsis* leaves. Proceedings of the National Academy of Sciences of the United States of America 100: 8583-8588.

S64. Hunt L, Mills LN, Pical C, Leckie CP, Aitken FL, et al. (2003) Phospholipase C is required for the control of stomatal aperture by ABA. The Plant Journal 34: 47-55.

S65. Kim TH, Bohmer M, Hu H, Nishimura N, Schroeder JI (2010) Guard cell signal transduction network: advances in understanding abscisic acid, CO_2_, and Ca^2+^ signaling. Annual Review of Plant Biology 61: 561-591.

S66. Lino B, Baizabal-Aguirre VM, Gonzalez de la Vara LE (1998) The plasma-membrane H^+^-ATPase from beet root is inhibited by a calcium-dependent phosphorylation. Planta 204: 352-359.

S67. Blatt MR (1990) Potassium channel currents in intact stomatal guard cells: rapid enhancement by abscisic acid. Planta 180: 445-455.

S68. Shimazaki K, Gotow K, Kondo N (1982) Photosynthetic properties of guard cell protoplasts from *Vicia faba*. Plant and Cell Physiology 23: 871-881.

S69. Wu W, Assmann SM (1993) Photosynthesis by guard cell chloroplasts of *Vicia faba* L. Effects of factors associated with stomatal movement. Plant and Cell Physiology 34: 1015-1023.

S70. Shimazaki K, Zeiger E (1985) Cyclic and noncyclic photophosphorylation in isolated guard cell chloroplasts from *Vicia faba* L. Plant Physiology 78: 211-214.

S71. Neill S, Bright J, Desikan R, Hancock J, Harrison J, et al. (2008) Nitric oxide evolution and perception. Journal of Experimental Botany 59: 25-35.

S72. Blatt MR, Grabov A (1997) Signal redundancy, gates and integration in the control of ion channels for stomatal movement. Journal of Experimental Botany 48: Special Issue, 529-537.

S73. Levchenko V, Konrad KR, Dietrich P, Roelfsema MR, Hedrich R (2005) Cytosolic abscisic acid activates guard cell anion channels without preceding Ca^2+^ signals. Proceedings of the National Academy of Sciences of the United States of America 102: 4203-4208.

S74. Zhang X, Takemiya A, Kinoshita T, Shimazaki K (2007) Nitric oxide inhibits blue light-specific stomatal opening via abscisic acid signaling pathways in *Vicia* guard cells. Plant and Cell Physiology 48: 715-723.

S75. Kwak JM, Mori IC, Pei ZM, Leonhardt N, Torres MA, et al. (2003) NADPH oxidase AtrbohD and AtrbohF genes function in ROS-dependent ABA signaling in *Arabidopsis*. The EMBO Journal 22: 2623-2633.

S76. Grabov A, Leung J, Giraudat J, Blatt MR (1997) Alteration of anion channel kinetics in wild-type and *abi1-1* transgenic *Nicotiana benthamiana* guard cells by abscisic acid. The Plant Journal 12: 203-213.

S77. Lee Y, Choi YB, Suh S, Lee J, Assmann SM, et al. (1996) Abscisic acid-induced phosphoinositide turnover in guard cell protoplasts of *Vicia faba*. Plant Physiology 110: 987-996.

S78. Zhang W, Qin C, Zhao J, Wang X (2004) Phospholipase D alpha 1-derived phosphatidic acid interacts with ABI1 phosphatase 2C and regulates abscisic acid signaling. Proceedings of the National Academy of Sciences of the United States of America 101: 9508-9513.

S79. Assmann SM (1994) Ins and outs of guard cell ABA receptors. The Plant Cell 6: 1187-1191.

S80. Sharkey TD, Raschke K (1981) Effect of light quality on stomatal opening in leaves of *Xanthium strumarium* L. Plant Physiology 68: 1170-1174.
