# Supplementary material for: Correction: Multi-level Modeling of Light-Induced Stomatal Opening Offers New Insights into Its Regulation by Drought
Source: PLoS Comput Biol. 2016 Nov 30;12(11):e1005181. doi: 10.1371/journal.pcbi.1005181 (PMC5130166; doi:10.1371/journal.pcbi.1005181)
Supplement: S1 Text — (DOCX) [file pcbi.1005181.s001.docx]

**Text S1: Description of node levels and updating rules**

**Node states**

The network (see Figure 1) has four source (signal) nodes: blue light, red light, ABA, and CO_2_. The state variables (levels) of blue light, red light, and ABA are 1 (ON) and 0 (OFF). The CO_2_ level can be 0 (CO_2_-free air), 1 (air with ambient atmospheric CO_2_ concentration), or 2 (air with high CO_2_ concentration). A list of all the available levels of each node is given below.

| Possible node levels | List of nodes |
| --- | --- |
| {0, 1} | blue light, phot1, phot2, 14-3-3 protein_phot1_, phot1_complex_, PLC,  PLA_2_β, CaIC, CaR, PIP2_c_, PIP2_PM_, NIA12, nitrate, nitrite,  Atnoa1, NO, Ca-ATPase, PRSL1, PP1_cn_, AtABCB14,  14-3-3 protein_H+-ATPase_, LPL, FFA, CDPK, mitochondria,  acid. apo, K_in_, K_out_, KEV, [NO_3_^-^]_a_, CHL1, [NO_3_^-^]_c_, [Cl^-^]_c_,  [NO_3_^-^]_v_, [Cl^-^]_v_, AtSTP1, red light, NADPH, starch, ABA,  ABA receptors, ROP2, ABI1, OST1, Atrboh, ROS, RIC7 |
| {0, 1, 2} | [Ca^2+^]_c_, CO_2_, photophosphorylation, carbon fixation, PLD, PA, [malate^2-^]_a_, sucrose |
| {0, 1, 1.6} | AnionCh |
| {0, 1, 2, 3} | ATP |
| {0, 0.5, 1, 2} | C_i_ |
| {0, 0.5, 1, 2, 3} | H^+^-ATPase |
| {0, 0.5, 1, 2, 4} | mesophyll cell photosynthesis |
| {–2, –1, 0, 1, 2} | PMV |
| {0, 1, 1.5, 2, 3, 3.5, 4} | PP1_cc_ |
| {0, 0.5, 0.9, 1, 1.5, 2, 3} | protein kinase (participating in H^+^-ATPase activation) |
| {0, 0.5, 1, 1.5, 2, 3, 4, 6, 9} | H^+^-ATPase_complex_, [K^+^]_c_, [K^+^]_v_, PEPC |
| {0, 0.25, 0.5, 0.75, 1, 1.25, 1.5, 2, 2.5, 3, 3.5, 4, 4.5, 5, 6, 6.5, 9, 9.5} | [malate^2-^]_c_, [malate^2-^]_v_ |
| more than 20 levels, ranges between 0 and 14.18 | stomatal opening |

A fraction of the node states listed above are defined through truth tables. Specifically, there are four such nodes: PP1_cc_, protein kinase (participating in H^+^-ATPase activation), C_i_, and AnionCh. The states of these nodes for specific values of their regulators are modeler-chosen, using the available biological information as guidance. This is similar to estimating the values of kinetic parameters in a continuous model. We define the levels of these four nodes, together with the basic Boolean states of 0 and 1, to be the fundamental states of the model. There are 10 such fundamental states: {0, 0.5, 0.9, 1, 1.5, 1.6, 2, 3, 3.5, 4}. Based on the 10 states, and on the updating rules not given as truth tables, a spectrum of more than 30 states is obtained; these states are therefore defined as derived states. Note that a derived state of a node other than PP1_cc_, protein kinase, C_i_, and AnionCh can have the same level as a fundamental state, but only the appearance of this state in the truth tables defining the update rules of these 4 nodes is fundamental.

All node levels, whether binary or multi-valued, fundamental or derived, represent qualitative categories. Thus level 2 should not be interpreted as an abundance or strength that is twice as high as the abundance or strength associated with level 1. Rather, it is an abundance or strength that is higher than that of state 1. The relative comparisons between states (e.g. A is higher than B) are more informative than the absolute values of the states.

**State space of the system**

The total number of potential system states can be obtained from the list of node states (activity levels) in the table above as $\prod{a_{i}}^{b_{i}}$, in which $a_{i}$ is the number of states (levels) and $b_{i}$ is the number of nodes that have $a_{i}$ allowed states. For instance, there are 47 nodes that have 2 states available to them; their contribution to the total number of system states is 2^47^. The total number of system states is therefore obtained by:

2^47^×3^9^×4^2^×5^3^×7^2^×9^4^×18^2^×20 ≈ 10^31^

The size of the state space of a Boolean model with the same number of nodes would be 2^70^ ≈ 10^21^. The large difference in the size of the state space of our model and a Boolean model with the same number of nodes exemplifies the complexity of our model.

**Initial states of the nodes**

The initial states of the four signal nodes (blue light, red light, CO_2_, and ABA) are chosen to reproduce relevant experimental conditions. The signal nodes’ states are not regulated by any other node in the network, and will stay constant throughout the simulation unless they are externally changed at a certain time step to represent time-varying signal inputs to the system, e.g. during a blue light pulse experiment. The initial state of intercellular CO_2_ (C_i_) is initialized to have the same value as atmospheric CO_2_, indicating a steady state C_i_ in the dark prior to receiving the light signal. The initial state of stomatal opening is chosen to be zero (representing closed stomata).

Apart from the four signal nodes, there are a total of 7 nodes whose states are not regulated by other nodes in the network. They are: 14-3-3 protein that binds to phot1 (14-3-3 protein_phot1_), PIP2 in the cytosol (PIP2_c_), nitrate, the nitric oxide-associated 1 protein (Atnoa1), the catalytic unit of the type 1 protein phosphatase localized in the nucleus (PP1_cn_), mitochondria, and the nitrate transporter AtNRT1.1 (CHL1). We assume that their activity or concentration levels are sufficiently high prior to receiving the light signal, thus their states are fixed at 1 (ON). We also assume that the concentrations of starch and apoplastic nitrate ([NO_3_^-^]_a_) are not the limiting factors for related functions in our simulations; these two nodes are therefore initialized at level 1 (ON) as well.

The initial states of the other 57 intermediary nodes in the network are initialized as 0 (OFF) to represent their resting states before receiving a signal.

**Node update functions**

A Boolean clause, e.g. “*element C* > 0”, is used in some rules to convert the value of a multi-level node *C* into a Boolean variable for the evaluation of downstream Boolean rules. If the condition is satisfied (“*element C* > 0” is TRUE), the clause yields 1; if the condition is *not* satisfied (“*element C* > 0” is FALSE), the clause yields 0.

In the following we indicate the update functions of single nodes or groups of two or three related nodes and document the evidence supporting this functional form. The node names are identical to the node names in Figure 1.

**phot1^*^ = blue light**

**phot2^*^ = blue light**

**phot1_complex_^*^ = phot1 *And* 14-3-3 protein_phot1_**

Phototropin 1 (phot1) and phototropin 2 (phot2) are established blue light receptors in guard cells [9, 14-16].

Phototropin 1 binds reversibly to a 14-3-3 protein (14-3-3 protein_phot1_) upon the auto-phosphorylation of phototropin 1 in guard cells [102, 103]. The 14-3-3 protein-phototropin complex (phot1_complex_) is thought to confer an active state to phototropin 1, which then transmits the light signal to downstream elements. The *And* function connects phot1 and the 14-3-3 protein, indicating that the formation of the complex requires both of them.

**CaIC^*^ = ROS *And* (PMV ≤ 0)**

**CaR^*^ = NO *Or* PLC**

**PLC^*^ = phot2 *Or* (ABA *And* ([Ca^2+^]_c_ > 0))**

Calcium influx through channels is active in the presence of plasma membrane hyperpolarization (PMV ≤ 0) and ROS production [76].

Phospholipase C (PLC) catalyzes InsP_3_ (inositol-1,4,5-trisphosphate) synthesis in guard cells, which induces Ca^2+^ release into the cytosol from internal stores (CaR) [43]. Two intermediary signaling molecules, cGMP and cADPR, also elicit Ca^2+^ release into the cytosol from internal stores (CaR), and both are downstream of NO. We combine and compress the cGMP and cADPR mechanisms and include a single edge from NO to CaR. Since this mechanism is independent from the PLC mediated mechanism, we assume that either of them is sufficient to drive CaR.

phot2 stimulates PLC-mediated Ca^2+^ release from intracellular stores, as inferred from assays with pharmacological inhibitors of PLC [16]. We incorporate this evidence by assuming that phot2 activates PLC. ABA activates PLC [43]. Since PLC-induced Ca^2+^ release to the cytosol from internal stores requires Ca^2+^ [S1], we assume that the combined effect of ABA and of a positive Ca^2+^ level is necessary to activate PLC in the absence of a light signal.

**LPL^*^ = PLA_2_β**

**FFA^*^ = PLA_2_β**

**PLA_2_β^*^ = phot1_complex_ *Or* phot2 *Or* red light**

PLA_2_β catalyzes the hydrolysis of phospholipids into lysophospholipids (LPL) and free fatty acids (FFA).

White light induces the expression of PLA_2_β in *Arabidopsis* [S2]. We assume that either blue or red wavelengths of light can trigger PLA_2_β expression, and that the blue component of the trigger is mediated by blue light-specific photoreceptors, the phototropins. An *Or* rule has been placed between the phototropins and red light, reflecting the possibility that either wavelength of light could induce above-threshold PLA_2_β expression.

**nitrite^*^ = nitrate *And* NIA1**

**NO^*^ = Atnoa1 *And* nitrite *And* NADPH *And* NIA1**

**NIA1^*^ = ROS**

The three equations above describe nitric oxide generation in guard cells. NIA1 catalyzes the production of nitrite from nitrate [S3].

NADPH is an electron donor in the reduction of nitrite to NO [S4]. NIA1 catalyzes the production of NO from nitrite [S3]. The protein Atnoa1 is also involved in NO accumulation [S5, S6]. No evidence suggests that Atnoa1 alone is sufficient for guard cell NO production, thus it is combined with other factors through an *And* rule.

Genetic and pharmacological evidence demonstrates that reactive oxygen species (mainly H­_2_O_2_) are upstream of ABA-induced NO production [S4, S7]. We thus assume that NIA1 is positively regulated by ROS.

**[Ca^2+^]_c_^*^ = ((CaIC *Or* CaR) *And Not* Ca^2+^-ATPase) + ABA**

**Ca^2+^-ATPase^*^ = ([Ca^2+^]_c_ > 0)**

Cytosolic Ca^2+^ concentration increases transiently in response to blue light; this response is mediated by phototropins [52, 53]. The increase is due to calcium influx through channels (CaIC) or to Ca^2+^ release into the cytosol from internal stores (CaR) [S8]. We assume that these processes are independent and either of them is sufficient to lead to an increase in the cytosolic Ca^2+^ concentration. We implement Ca^2+^ decrease in the form of a Ca^2+^ efflux from the cytosol via the activity of Ca^2+^ ATPases and Ca^2+^/H^+^ antiporters, represented by the node Ca**^2+^**-ATPase in our model. ABA is able to induce Ca^2+^ oscillations [43, S9]. The average cytosolic Ca^2+^ concentration is higher during oscillations induced by ABA than during transient increase induced by blue light [43, 52, S9], thus we assume that the presence of ABA raises the cytosolic Ca^2+^ level by one level (from 0 to 1 or from 1 to 2). For compactness we represent this assumption by incorporating ABA as an additive enhancer of Ca^2+^.

The activity of the Ca**^2+^**-ATPase is activated by cytosolic Ca^2+^.

**KEV^*^ = [K^+^]_v_ *And* ([Ca^2+^]_c_ = 2)**

K^+^ release into the cytosol (KEV) through K^+^-permeable channels in the tonoplast requires the presence of vacuolar K^+^ ([K^+^]_v_), and is induced by cytosolic calcium [7, S10-S12]. We incorporate a requirement for high Ca^2+^ concentration, as reported by experiments [S12] by the clause “And ([Ca^2+^]_c_ = 2)”, meaning that K^+^ release into the cytosol does not happen if the cytosolic Ca^2+^ level is 0 or 1.

**PRSL1^*^ = phot1_complex_ *Or* phot2**

**PP1_cc_^*^ truth table.** *a* and *b* are wildcard values equivalent to listing all possible states of PRSL1 and PA. Their use indicates that the **PP1_cc_^*^ = 0** outcome is independent of the state of PRSL1 and PA, if **PP1_cn_ = 0**.

| **PP1_cn_** | **PRSL1** | **PA** | **PP1_cc_^*^** |
| --- | --- | --- | --- |
| **0** | ***a*** | ***b*** | **0** |
| **1** | **0** | **0** | **2** |
|  |  | **1** | **1.5** |
|  |  | **2** | **1** |
|  | **1** | **0** | **4** |
|  |  | **1** | **3.5** |
|  |  | **2** | **3** |

Multiple experiments confirm that type 1 protein phosphatase (PP1) mediates signaling between phototropins and the plasma membrane H^+^-ATPase [S13, S14]. The regulatory subunit of PP1, the PP1 regulatory subunit 2-like protein1 (PRSL1), stimulates the translocation of the catalytic subunit of PP1, PP1_c_, from the nucleus (PP1_cn_) to the cytosol (PP1_cc_) [S15]. We assume that PRSL1 is downstream of phototropins and upstream of PP1.

PP1_cn_ is required for the activation of PP1_cc_, therefore PP1_cc_ is 0 if PP1_cn_ is absent regardless of the values of PRSL1 and PA. We assume that PRSL1 promotes the translocation of PP1_c_, inducing higher values of PP1_cc_ when PRSL1 is 1.

Evidence shows that phosphatidic acid (PA) inhibits the activity of protein phosphatase 1 [17]. Based on experimental evidence [44, S6], we implemented a multi-value rule for PP1_cc_ to differentiate between the inhibitory strengths of the signal from PA, which itself has three levels.

**PIP_2PM_^*^ = (phot1_complex_ *Or* phot2) *And* PIP_2c_**

Experiments suggest that the level of PIP_2_ at the plasma membrane (PIP_2PM_) increases upon white light illumination due to the translocation of PIP_2_ from the cytosol (PIP_2c_) to the plasma membrane [S16]. We assume that PIP2 translocation is activated by the blue component of white light only, and that phototropins mediate this signaling. Our model indicates that if red light were to activate this translocation, the plasma membrane would stay hyperpolarized under red light after a pulse of blue light is given. This contradicts the observations that a pulse of blue light leads to transient, rather than sustained, stomatal opening [17] and that in ambient air, red light is ineffective in activating the H^+^-ATPase [18, 64, 80] (see Discussion in the manuscript), the primary engine driving the plasma membrane hyperpolarization [18, 64, 79, 80].

**PA^*^ = PLD**

**PLD^*^ = ABA + NO**

Phospholipase D (PLD) catalyzes the hydrolysis of [phosphatidylcholine](http://en.wikipedia.org/wiki/Phosphatidylcholine) to form [phosphatidic acid](http://en.wikipedia.org/wiki/Phosphatidate) (PA).

Both ABA and NO have been indicated to induce PLD-catalyzed PA production [42, S17, S18], thus we assume that both ABA and NO can activate PLD. ABA and NO are each capable of triggering stomatal closure via PLD [42, S6, S17]. In addition, experiment showed that NO scavenger partially restored ABA inhibition of light-induced stomatal opening [S6], indicating a non-independent relationship between ABA and NO. Therefore, we assume that the action of ABA and NO in the activation of PLD is additive.

**ABA receptors^*^ = ABA**

**ABI1^*^ = *Not* ABA receptors**

**OST1^*^ = *Not* ABI1**

**ROP2^*^ = phot1_complex_ *Or* phot2 *Or* red light *Or* PA**

**AtrbohD/F^*^ = (OST1 *And* PA) *Or* (OST1 *And* PA *And* ROP2)**

**ROS^*^ = NADPH *And* AtrbohD/F *Or* NADPH *And* AtrbohD/F *And* CDPK *Or Not* Atnoa1**

Several ABA receptors have been identified, including the Pyrabactin Resistance/Pyrabactin Resistance-Like/Regulatory Components of ABA Receptor protein family [S19-S23] and G-protein coupled receptor –type G proteins [S24, S25]. We include a general node for all ABA receptors (including as-of-yet unidentified ones), and the node is thus activated by ABA.

The Pyrabactin Resistance/Pyrabactin Resistance-Like/Regulatory Component family of ABA receptors, when bound by ABA, down-regulates ABI1 and related PP2C phosphatases (represented by ABI1) [S19-S22, S26].

OST1 is inactivated by ABI1 [S27, S28] and is active in the presence of ABA [S28]. Since ABA indirectly inhibits ABI1, “not ABI1” already incorporates the indirect positive regulation of OST1 by ABA and thus is not necessary to explicitly include.

White light has been observed to induce the translocation of the small G protein ROP2 to the plasma membrane of guard cells, thereby activating it [74]. We assume that either blue or red light can activate ROP2, and that the blue component of the activation is mediated by blue light-specific photoreceptors phot1 and phot2. There is evidence that PA induces ROS generation through the activation of ROP2, thus we include PA as a positive regulator of ROP2 [S29]. We combine the activators of ROP2 with *Or* rules, since no synergistic effect between the activators is known.

ABA fails to elicit reactive oxygen species (ROS) production in an *ost1* mutant [S30], implying that OST1 is a required positive regulator of the NADPH oxidase (AtrbohD/F). Evidence shows that PA induces ROS generation through the activation of ROP2 [S29], but ROP2 alone is not sufficient to induce ROS elevation [S29]. New evidence shows that PA also activates AtrbohD/F directly [S31]. We thus assume that ROP2 is effective only in conjunction with PA and OST1, and that PA in conjunction with OST1 can activate AtrbohD/F.

Reactive oxygen species are produced in a reaction catalyzed by AtrbohD/F. ABA and Ca^2+^ influx through plasma membrane channels (CaIC) were impaired in CDPK (6 and 3) mutants, revealing a feedback mechanism from CDPK to ABA signaling [S32]. The feedback is suggested to target ROS or its upstream nodes. We incorporate this information by making CDPK a contributing factor to ROS production. Additionally, ROS elevation has been observed in *Atnoa1* knockout mutants [S33, S34]. This is incorporated by making the absence of Atnoa1 a sufficient condition for ROS production.

**ATP^*^ = photophosphorylation + mitochondria**

ATP is provided by two primary mechanisms in guard cells: photophosphorylation and mitochondrial respiration. Since the two mechanisms are relatively independent, we model their contribution to the total amount of ATP as additive.

**protein kinase^*^ truth table.** *a* is a wildcard value equivalent to listing all possible states of C_i_. Its use indicates that the **protein kinase^*^ = 0** outcome is independent of the specific value of C_i_, if **PP1_cc_ = 0**. *b* is a wildcard value equivalent to listing all possible states of PP1_cc_. Its use indicates that the **protein kinase^*^ = 0** outcome is independent of the specific value of PP1_cc_, if **C_i_ = 2**.

| **C_i_** | **PP1_cc_** | **protein kinase^*^** |
| --- | --- | --- |
| ***a*** | **0** | **0** |
| **0** | **1, 1.5** | **0.5** |
|  | **2** | **1** |
|  | **3, 3.5** | **1.5** |
|  | **4** | **3** |
| **0.5** | **1, 1.5** | **0** |
|  | **2** | **0.5** |
|  | **3** | **0.5** |
|  | **3.5** | **1.5** |
|  | **4** | **2** |
| **1** | **1, 1.5, 2** | **0** |
|  | **3** | **0.5** |
|  | **3.5** | **0.9** |
|  | **4** | **1** |
| **2** | ***b*** | **0** |

**H^+^-ATPase^*^ = protein kinase**

**14-3-3 protein_H+-ATPase_^*^ = (H^+^-ATPase > 0)**

**H^+^-ATPase_complex_^*^ = [(FFA *Or* LPL) *And Not* ([Ca^2+^]_c_ = 2) *And* 14-3-3 protein_H+-ATPase_] × H^+^-ATPase × ATP**

A serine/threonine protein kinase that directly phosphorylates the plasma membrane H^+^-ATPase is required for effective pump activity; this kinase has yet to be explicitly identified at the molecular level and so it is designated generically as protein kinase [36, S35]. We assume that PP1 functioning, which mediates signaling between phototropins and the H^+^-ATPase [S13, S14], occurs upstream of the phosphorylation of the H^+^-ATPase by the protein kinase. It was suggested that high CO_2_ inhibits the proton pump activity [86]. We hypothesize that C_i_ applies a brake on the H^+^-ATPase activity through regulating the protein kinase in a concentration-dependent manner, and that different activity levels of PP1_cc_ also affect the activity of the protein kinase. The truth table summarizes the possible states of the protein kinase for all state combinations of its regulators. Lowering C_i_ lifts the brake, resulting in an equal or higher value for protein kinase, *ceteris paribus*. If PP1_cc_ = 0, the protein kinase will not be activated. If PP1_cc_ is at its baseline activity level (value 1), the protein kinase is active only when C_i_ is 0. If PP1_cc_ activity is promoted (value 3 or higher), the protein kinase activity level is increasingly higher with lower C_i_.

The protein kinase phosphorylates the plasma membrane H^+^-ATPase. Phosphorylation occurs exclusively at the C terminus of the H^+^-ATPase, an autoinhibitory domain of the H^+^-ATPase, thus activating the H^+^-ATPase.

A 14-3-3 protein binds to the phosphorylated C terminus of the H^+^-ATPase, [16, 65, S36]. The H^+^-ATPase’s phosphorylation by the protein kinase is a pre-condition of the 14-3-3 protein’s ability to bind to it.

The complex formed by the H^+^-ATPase and the 14-3-3 protein is denoted as H^+^-ATPase_complex_. We assume that this complex is necessary for H^+^ extrusion and membrane hyperpolarization. FFA and LPL have been shown to promote proton pump activity [S2, S37]. Experiments have indicated that a high level of [Ca^2+^]_c_ reversibly inhibits the plasma membrane H^+^-ATPase [56], but [Ca^2+^]_c_ was also suggested to play a positive role in blue-light signaling in guard cells [50, S38]. Here we incorporate the inhibitory effect from [Ca^2+^]_c_ with an *And Not* rule, but add a concentration requirement **([Ca^2+^]_c_ = 2)** for the inhibition to be effective. Further evidence has to be acquired to completely elucidate the role of [Ca^2+^]_c_ in blue light-induced stomatal opening. H^+^-ATPase_complex_ activity is powered by ATP [4, 80], hence its activity level depends on the amount of ATP that is available. When ATP is abundant, e.g. under the action of both types of light, H^+^-ATPase_complex_ activity is also at a high value. At an intermediate ATP level, the activity of the proton pump will be lower (but above threshold). When no ATP is available, the H^+^-ATPase_complex_ activity will be shut off. The activity level of the H^+^-ATPase_complex_ depends on the degree of phosphorylation and the abundance of ATP in a more quantitative way than for most other nodes, thus the phosphorylated state of the H^+^-ATPase and ATP are implemented as multipliers in the rule of the H^+^-ATPase_complex_.

**CDPK^*^ = ([Ca^2+^]_c_ > 0) *And* (ATP > 0)**

The activation of calcium dependent protein kinases (CDPK) requires ATP and Ca^2+^ [54].

**PMV^*^ = PMV – (H^+^-ATPase_complex_ > 0) + (AnionCh *And* (PMV < 0)) + (([Ca^2+^]_c_ = 2) *Or* KEV)**

PMV is the difference of electric potential across the plasma membrane. Computationally, we use the value 0 to represent the resting potential of the plasma membrane; this value should not be confused with actual values of the membrane potential. The decrease of PMV represents plasma membrane hyperpolarization, and the increase of PMV represents plasma membrane depolarization. We assume that five levels (two negative, 0, two positive) are sufficient for a qualitative description, and require that PMV value stays bounded, i.e. the value will not further decrease (or increase) when it reaches -2 (or 2). The future PMV value (PMV^*^) is calculated based on its current value (PMV). Factors that cause hyperpolarization will decrease the PMV value, shifting it more negative, and factors that cause depolarization will increase the PMV value, shifting it less negative. An active H^+^-ATPase complex causes the extrusion of H^+^ from the guard cell cytosol to the apoplast, hyperpolarizing the plasma membrane. Anion efflux at the plasma membrane causes the plasma membrane to depolarize. A steady anion efflux requires active anion efflux channels (AnionCh) and it also requires plasma membrane hyperpolarization. A significant increase in [Ca^2+^]_c_ (value 2) or K^+^ release into the cytosol from vacuole are modeled as independent factors causing the plasma membrane to depolarize.

**photophosphorylation^*^ = blue light + red light**

**NADPH^*^ = (photophosphorylation > 0)**

**carbon fixation^*^ = ((CO_2_ > 0) *Or* (C_i_ > 0)) × photophosphorylation**

Guard cell photosynthesis can be activated by blue or red light. The rate of photosynthetic photophosphorylation depends on the intensity of illumination. The qualitative variable representing photophosphorylation is assumed to be 0 in the absence of light, 1 in the presence of light of either quality, and 2 in the presence of both types of light.

The reduced form of NADP^+^, NADPH, is generated by photophosphorylation.

Carbon fixation requires atmospheric CO_2_ or intracellular CO_2_ (C_i_). Since the reducing power in the form of NADPH and ATP consumed by carbon fixation is provided by photophosphorylation, photophosphorylation is incorporated as a multiplier in the rule of carbon fixation. The rule for ATP, the other non-carbohydrate production of photosynthesis, was given earlier.

**acid. apo^*^ = (H^+^-ATPase_complex_ > 0)**

**AtSTP1^*^ = acid. apo**

**sucrose^*^ = carbon fixation**

Acidification of the apoplast (acid. apo) is caused by extrusion of protons from the cytosol into the apoplast by active H^+^-ATPase_complex_ [36, 37, 51, 63].

H^+^/sucrose uptake symporters like AtSTP1 are stimulated by light and require acidification of the apoplast [34, 83, 84]. The upregulation of AtSTP1 is observed to be transient [84]. Due to the lack of information on the mechanism underlying this transient behavior, we do not explicitly include negative regulation or feedback into this rule.

Sucrose can be accumulated in guard cells via photosynthetic carbon fixation, starch degradation or import from the apoplast [6, 81]. Due to the lack of experimental data providing information on the relative amount of sucrose accumulated by each pathway, to the fact that starch degradation appears to primarily provide malate as counterion for K^+^ [6], and since light-induced AtSTP1 increase is transient [84], we assume that guard cell carbon fixation is the primary source of sucrose accumulation. This is supported by the fact that DCMU, known to disrupt photosystem II and therefore inhibit photophosphorylation and carbon fixation, completely inhibits red light-induced stomatal opening [3], and the fact that sucrose is shown to be the major osmoticum during red light-induced stomatal opening [3, 6, 35, 73].

Since the photosynthetic pathway in our network model is represented compactly, without showing the numerous intermediate metabolites involved, we introduced a linear series of 10 virtual nodes between the node carbon fixation and the node sucrose. The state of each node in the series is determined by the state of its predecessor node. The predecessor of the first node in the series is carbon fixation; the last node in the series is the predecessor of sucrose. The time delay introduced by the series of virtual nodes aims to ensure that red light- and blue light-induced stomatal opening rates are in accordance with experimental observations [4, 57]. We verified that a delay anywhere in the range from 1 time step to 20 time steps leads to stomatal opening.

**K_in_^*^ = (FFA *Or Not* (([Ca^2+^]_c_ = 2) *Or* ABA)) *And Not* (C_i_ = 2) *And* (PMV < 0)**

**K_out_^*^ = (ABA *Or* (C_i_ = 2) *Or Not* ROS *Or Not* NO *Or Not* FFA) *And* (PMV > 0)**

**[K^+^]_v_^*^ = [K^+^]_c_**

Polyunsaturated fatty acids (FFA) have been found to enhance light-induced stomatal opening and to potentiate K^+^ currents through inward K^+^ channels (K_in_) in guard cells [S2, S39]. FFA is therefore assumed to be a promoter of K_in_. ABA and [Ca^2+^]_c_ can independently inhibit K_in_ [S40], therefore they are joined with *Or*. We impose a requirement for high [Ca^2+^]_c_ for its inhibitory effect to be effective [55, S41]. We assume that the presence of FFA is connected with the absence of ABA or of a high level of [Ca^2+^]_c_ by an *Or* rule. A high level of intracellular CO_2_ (C_i_) inhibits K_in_ [S42], and is connected with other factors with an *And Not* rule. Voltage-gated K_in_ currents are activated by plasma membrane hyperpolarization (PMV<0) [S43], therefore the electric potential requirement (PMV<0) is connected to the other clauses with an *And* rule.

Plasma membrane depolarization (PMV>0) drives K^+^ efflux from the cell through outwardly rectifying channels (K_out_). The activity of outwardly rectifying K^+^ channels is activated by ABA [S44, S45], or high levels of C_i_ [S42], and is inhibited by FFA [S2, S39], ROS [S46] or nitric oxide [S47]. Other than the status of the membrane potential, no direct evidence has thus far been obtained regarding the potential superiority of one upstream factor over another one that is counter-acting on the activity of K_out_, so for generality an *Or (Not)* rule is implemented among all inputs other than the membrane potential. The underlying idea is: i) no combinatorial/synergistic effect is assumed, and ii) the clause yields a zero only when all *Or*–separated factors fail.

Cytosolic K^+^ is able to flow into vacuoles and contribute to the production of turgor pressure.

**[K^+^]_c regulation_ = (K_in_ *Or* [K^+^]_v_ *And* KEV) *And* H^+^-ATPAse_complex_ *And Not* K_out_**

**[NO_3_^-^]_c regulation_ = [NO_3_^-^]_a_ *And* CHL1 *And* (H^+^-ATPAse_complex_ ≥ AnionCh)**

**[Cl^-^]_c regulation_ = (H^+^-ATPAse_complex_ ≥ AnionCh)**

**[malate^2-^]_c regulation_ = (*Not* (mitochondria *And* ABA)) *And* (H^+^-ATPAse_complex_ ≥ AnionCh)**

**Anion_regulation_ = [NO_3_^-^]_c regulation_ *Or* [Cl^-^]_c regulation_ *Or* [malate^2-^]_c regulation_**

**[K^+^]_c_^*^ = H^+^-ATPAse_complex_ × [K^+^]_c regulation_ × Anion_regulation_**

**[NO_3_^-^]_c_^*^ = [K^+^]_c regulation_ × [NO_3_^-^]_c regulation_**

**[Cl^-^]_c_^*^ = [K^+^]_c regulation_ × [Cl^-^]_c regulation_**

**internal = (starch *Or* carbfix)×PEPC**

**import = [malate^2-^]_a_ *And* AtABCB14**

**[malate^2-^]_c_^*^ = 0.5 × (internal + import) × [K^+^]_c regulation_ × [malate^2-^]_c regulation_**

Since charge balance must be maintained between positive ions (K^+^) and negative ions (Cl^-^, NO_3_^-^, malate^2-^), a linked set of rules describes the update of the states of the ion nodes. This connection between rules needs to guarantee that the uptake of K^+^ is required for anion accumulation, and the presence of anions is also required for K^+^ uptake. This is implemented by pre-calculating a regulatory (Boolean) variable for each ion node (denoted by the subscript “regulation”), which indicates the condition for the corresponding node state to be non-zero. The values of the ion node states are then found by multiplication of the regulatory variables with each other and with other regulators. The presence of any anion species would suffice to compensate for charge balance with [K^+^]_c_ which is why we define the variable Anion_regulation_ which is 1 if any of the three anion regulatory variables are 1; this variable is used in the rule of [K^+^]_c_. On the other hand, the presence of [K^+^]_c_ is required by any anion species, which is indicated by the appearance of [K^+^]_c regulation_ in the rules for all three anions.

Cytosolic K^+^ concentration ([K^+^]_c_) is one of the crucial factors that determine stomatal aperture. K^+^ is one of the major osmotica present in guard cells, the flow of which drives stomatal movement: K^+^ uptake leads to water uptake and swelling of the cell with subsequent stomatal opening, and K^+^ efflux leads to water loss and stomatal closure. Cytosolic K^+^ concentration ([K^+^]_c_) increase can result from K^+^ uptake via K_in_ or K^+^ release from the vacuole (KEV) which requires the presence of [K^+^]_v_ and active KEV. Thus the regulatory variable [K^+^]_c regulation_ includes the clause K_in_ *Or* [K^+^]_v_ *And* KEV. Maintenance of [K^+^]_c_ requires the absence of K_out_ and proper membrane potential provided via proton extrusion from the cytosol to the apoplast by active H^+^-ATPase [37, 38]. K_out_ thus enters the regulatory variable [K^+^]_c regulation_ using an *And Not* rule. If the regulatory requirements are met, [K^+^]_c_ is assumed to scale with the activity of H^+^-ATPase_complex_ through a multiplication rule. The requirement for charge balance is represented by including Anion_regulation_ in the rule for [K^+^]_c._

The cytosolic NO_3_^-^ level ([NO_3_^-^]_c_) increases by NO_3_^-^ uptake from the apoplast to the cytosol mediated by the NO_3_^-^ transporter AtNRT1.1 (CHL1) [S48]. Thus the regulatory variable [NO_3_^-^]_c regulation_ requires both [NO_3_^-^]_a_ *And* CHL1. The competition between the relative strengths of the forces driving ion accumulation and depletion is represented by a comparison between H^+^-ATPAse_complex_ and AnionCh; this comparison will be used for all anionic species. If the accumulation drive is higher than the leakage (H^+^-ATPAse_complex_ ≥ AnionCh), the regulatory variables of anions are nonzero and anion concentrations would be able to successfully rise. The requirement of charge balance is represented by including [K^+^]_c regulation_ in the quantitative part for [NO_3_^-^]_c_.

Similar to the increase of [NO_3_^-^]_c_, a net accumulation stimulus is required by [Cl^-^]_c_ (H^+^-ATPAse_complex_ ≥ AnionCh), and the requirement of charge balance is represented by having [K^+^]_c regulation_ in the quantitative part for [Cl^-^]_c_.

ABA induces malate breakdown by the TCA cycle [S49]. This is represented by combining ABA with mitochondria using *And* in the regulatory variable [malate^2-^]_c regulation_, and the combination is considered a strong suppressor of [malate^2-^]_c_ reflected by the operator *Not* required by [malate^2-^]_c_ accumulation. Like the cytosolic concentrations of other anions, [malate^2-^]_c_ accumulation requires a proper membrane potential—anion efflux relationship (H^+^-ATPAse_complex_ ≥ AnionCh).

Three mechanisms cause cytosolic malate ([malate^2-^]_c_) accumulation: starch degradation, carbon fixation, and malate import from the apoplast. Malate production from starch degradation and carbon fixation (described by the intermediate variable ‘internal’) is catalyzed by PEPC and henceforth we assume that it scales with the activity level of PEPC through a multiplication rule.

If the requirements for malate accumulation are met, the [malate^2-^]_c_ level is proportional to the sum of ‘internal’ and ‘import’; the factor of 0.5 is in place to normalize the sum.

**[NO_3_^-^]_a_^*^ = [NO_3_^-^]_a_ *Or* [NO_3_^-^]_c_ *And* AnionCh *And* (PMV < 0)**

**[NO_3_^-^]_v_^*^ = [NO_3_^-^]_c_**

**[Cl^-^]_v_^*^ = [Cl^-^]_c_ *And* CDPK *Or* [Cl^-^]_c_**

We assume that apoplastic nitrate concentration ([NO_3_^-^]_a_) is not the limiting factor for related functions, so that as long as it was present in the previous time step, its level remains above threshold if initiated as such. This is implemented by a positive connection between the current [NO_3_^-^]_a_ level and its future level. Efflux of cytosolic NO_3_^-^ ([NO_3_^-^]_c_) to the apoplast can contribute to the increase of [NO_3_^-^]_a_ as well. This process requires active anion efflux channels (AnionCh) and a hyperpolarized membrane (PMV<0).

The vacuolar NO_3_^-^ level ([NO_3_^-^]_v_) is determined by [NO_3_^-^]_c_.

Ca^2+^-dependent protein kinases (CDPK) regulate transport of Cl^-^ from the cytosol ([Cl^-^]_c_) to the vacuole ([Cl^-^]_v_) [54]. The joint presence of CDPK and [Cl^-^]_c_ is thus a positive regulator of [Cl^-^]_v_. There has been no evidence showing that the activity of Cl^-^ -permeable channels in the vacuole membrane follows the transient nature of Ca^2+^ behavior under light, while CDPK is expected to follow it. We therefore assume that other, Ca^2+^-independent, kinases that phosphorylate the same site or other potential regulators are also involved in regulating Cl^-^ flow from the cytosol to the vacuole. In the absence of specific evidence we represent this Ca^2+^-independent component as being dependent on [Cl^-^]_c_ alone; this is joined with CDPK-regulated flow with an *Or* rule.

**PEPC^*^ = (*Not* ABA) × Max{1, H^+^-ATPase_complex_}**

Phospho*enol*pyruvate carboxylase (PEPC) activity is known to be inhibited by ABA [S50], making the absence of ABA a requirement for its activation. Highly active H^+^-ATPase_complex_ facilitates the activation of PEPC and thus yields a higher activation level of PEPC, as shown experimentally [S50, S51]. The dependence of PEPC activity upon H^+^-ATPase activity is implemented as a multiplier in the rule. Since there has not been evidence showing that the absence of proton pump activity should inhibit PEPC activity, the multiplier is the larger value between 1 and H^+^-ATPase_complex_. The Max function is to ensure that the proton pump enhances PEPC activity if the proton pump’s activity is high, and that low proton pump activity does not obstruct PEPC activity.

**MCPS^*^= (blue light + red light) × (C_i_ > 0)**

**[malate^2-^]_a_^*^ = ((MCPS > 0) *Or* ([malate^2-^]_c_ > 0) *And* (AnionCh > 0) *And* (PMV < 0)) × Max{1, C_i_ }**

Atmospheric CO_2_ concentration is represented by three levels of the node CO_2_, 0 (low concentration), 1 (moderate concentration) and 2 (high concentration). Mesophyll cell photosynthesis (MCPS) can be activated by blue or red light; we symbolically represent the dependence of MCPS on light intensities with an addition. The C_i_ multiplier reflects that in the presence of light, the more C_i_ there is to be utilized by photosynthesis, the more photosynthetic carbon reduction products there will be.

Apoplastic malate, [malate^2-^]_a_, can result from mesophyll cell photosynthesis. Malate that exits the guard cell cytosol through anion efflux can also contribute to [malate^2-^]_a_. A high level of [malate^2-^]_a_ has been suggested to mediate stomatal closure in response to elevated CO_2_ [S52]. The Max function is used to ensure that a high level of [malate^2-^]_a_ results from an excessive supply of C_i_.

For the calculation of C_i_, we first introduce the intermediate variable consumption, defined as

consumption = max{carbon fixation, MCPS}.

**C_i_^*^ truth table.** *a* is a wildcard value equivalent to listing all possible states of the intermediate variable consumption. Its use indicates that the outcome of C_i_^*^ is independent of the specific value of consumption, if **CO_2_ = 0** or **CO_2_ = 2**.

| **CO_2_** | **consumption** | **C_i_^*^** |
| --- | --- | --- |
| **0 (CO_2_-free air)** | ***a*** | **0** |
| **1 (moderate atmospheric CO_2_)** | **0 or 1** | **1** |
|  | **2** | **0.5** |
| **2 (high atmospheric CO_2_)** | ***a*** | **2** |

Two factors determine the level of C_i_: supply from CO_2_ in the atmosphere and consumption by photosynthetic carbon fixation. The baseline value of C_i_ primarily depends on the atmospheric CO_2_ concentration. If the ambient air is CO_2_-free, C_i_ is 0. If the atmospheric CO_2_ content is moderate, the C_i_ level further depends on the rate of consumption by photosynthetic carbon fixation in guard cells and mesophyll cells: if the consumption rate is moderate or low, C_i_ is 1; if the consumption is high (represented by the value 2), C_i_ is 0.5. If the atmospheric CO_2_ concentration is high, C_i_ reaches a high value as well (value 2).

**Anion_highactivation_ = (([Ca^2+^]_c_ = 2) *Or* ABA) *And Not* ABI1 *Or* (C_i_ = 2) *Or* ([malate^2-^]_a_ = 2)**

**AnionCh^*^ truth table.**

| **Anion_highactivation_** | **PIP2_PM_** | **AnionCh^*^** |
| --- | --- | --- |
| **0** | **0** | **1** |
|  | **1** | **0** |
| **1** | **0 or 1** | **1.6** |

The node AnionCh represents the status of the anion efflux channels. Anion flow through these channels directly controls the concentration of various anions in guard cells. Since it is known that anion efflux is regulated by multiple upstream nodes, we assume that these nodes influence the status of the anion efflux channels, and thus are part of the update function of AnionCh. An intermediate variable Anion_highactivation_ is created to summarize situations under which anion efflux channels are highly activated. Cytosolic Ca^2+^ and ABA can each promote the activation of AnionCh, leading to anion efflux [55, S53]. We require high [Ca^2+^]_c_ to effectively activate AnionCh [55]. ABI1, which is experimentally demonstrated to be a negative regulator of anion efflux [S54], is combined with the ABA-related activators by an *And Not* rule in the condition for Anion_highactivation_. High C_i_ [S42, S55, S56] or a high level of [malate^2-^]_a_ [S52] are strong activators of anion efflux channels; either is sufficient to activate high AnionCh levels. They are therefore connected with the effect of ABA via *Or* rules in the intermediate variable Anion_highactivation_.

Phototropins [S57] as well as PIP_2PM_ in response to light [S16] were reported to inhibit AnionCh. Since there is no evidence regarding the downstream targets of phototropins relevant to anion channel regulation or the upstream regulators of PIP2_PM_, we hypothesize that PIP_2PM_ is the mediator of the phototropin effect and the closest upstream regulator of AnionCh. The specific dependence of the AnionCh value on its regulators is given by the truth table: when none of the potent activators of AnionCh are present (Anion_highactivation_ = 0), AnionCh equals *Not* PIP2_PM_. When AnionCh is highly active, its value is chosen as 1.6. The relevance of this value is in the comparison of the AnionCh level with that of the H^+^-ATPAse_complex_, which enters the rules for ion flow.

**AtABCB14^*^ = ([malate^2-^]_a_ > 0)**

**[malate^2-^]_v_^*^ = [malate^2-^]_c_ × max{CDPK, 1}**

**starch^*^ = starch *Or* ([malate^2-^]_c_ > 0) *And* ABA**

Import of apoplastic malate ([malate^2-^]_a_) into the cytosol is mediated by the transporter AtABCB14 [S58].

The mechanism of vacuolar malate import is similar to Cl^-^ flow from the cytosol to the vacuole [54]. It is regulated by Ca^2+^-dependent protein kinases (CDPK). We assume that other, Ca^2+^-independent, kinases that phosphorylate the same site, or other potential regulators are also involved in regulating malate flow from cytosol to vacuole, represented by value 1 in combination with CDPK by a max function. If [malate^2-^]_v_ can be successfully accumulated, we assume that its value scales with the [malate^2-^]_c_ value using a multiplication rule.

We assume that the starch content in guard cells is not the limiting factor for related functions, so that as long as starch was present in the previous time step, its value will stay above threshold despite possible degradation. ABA can induce [malate^2-^]_c_ conversion back to starch [S49].

**RIC7^*^ = ROP2 × (stomatal opening ≥ 7)**

ROP-interactive CRIB motif–containing protein 7 (RIC7) is recruited by active ROP2 [74]. Since RIC7 provides a mechanism that prevents excessive stomatal opening [74], here we assume that RIC7 is only active if the stomatal opening level is equal or greater than 7, which is approximately half of the maximum stomatal opening level that can be acquired by the model.

We define intermediate variables for the contribution (shortened to contr. in the rule) of individual osmotica to stomatal opening.

[malate^2-^]_v_ contr.= min{[malate^2-^]_v_, 0.425 × [K^+^]_v_}

[NO_3_^-^]_v_ contr.= min{[NO_3_^-^]_v_, 0.10 × [K^+^]_v_}

[Cl^-^]_v_ contr.= min{[Cl^-^]_v_, 0.05 × [K^+^]_v_}

sucrose contr. = sucrose

The coefficients are chosen such that they reflect the contribution of each osmoticum to the osmotic potential. Malate is the primary anion [7, S59, S60], and we assume that it accounts for ~85% of negative charges, serving as a charge balancer for [K^+^]_v_. Considering that malate has a valence of two, we require [malate^2-^]_v_ to not exceed 42.5% of [K^+^]_v_. The analogous percentages for [NO_3_^-^]_v_ and [Cl^-^]_v_ are assumed to be 10% and 5%, respectively.

**stomatal opening^*^ = [Cl^-^]_v_ contr. + [NO_3_^-^]_v_ contr. + [K^+^]_v_ contr. + [malate^2-^]_v_ contr.+ sucrose contr. – 1/6 RIC7**

Finally, to reflect the fact that every type of osmoticum contributes to the osmotic potential of the guard cell, and thus to the induction and maintenance of stomatal opening, we assume that the level of stomatal opening is a sum of the contributions of all osmotica. RIC7 has been shown to be an attenuator of light-induced stomatal opening, preventing excessive opening upon perception of light [74]. This attenuation is incorporated as a subtraction, modulating the total level of stomatal opening by 1/6^th^ of the RIC7 value.
