# Supplementary material for: Correction: Multi-level Modeling of Light-Induced Stomatal Opening Offers New Insights into Its Regulation by Drought
Source: PLoS Comput Biol. 2016 Nov 30;12(11):e1005181. doi: 10.1371/journal.pcbi.1005181 (PMC5130166; doi:10.1371/journal.pcbi.1005181)
Supplement: S4 Table — (DOCX) [file pcbi.1005181.s002.docx]

**Table S4. Compilation of comparisons between published experimental observations and the model's results for simulations of the identical conditions.** In the column entitled Simulation Settings “BL” stands for blue light, “RL” stands for red light, “ABA” means abscisic acid, “CO_2_” indicates atmospheric CO_2_, and “C_i_” indicates intercellular CO_2_. The column entitled Results indicates the mean level (based on 2000 simulations) of the corresponding variable at steady state (after 18 time steps), unless indicated otherwise. The last column is a qualitative conclusion whether the simulations yield consistent results with experimental observations (the column title, C, stands for "Consistency"). The qualifier "C" in individual entries (meaning "consistent") indicates that the model qualitatively recapitulates the experimental observation. PC stands for partially consistent, and IC stands for "inconsistent".

| **Experimental Observation** | **References** | **Simulation Settings** | **Results** | **C** |
| --- | --- | --- | --- | --- |
| Under equal quantum flux, blue light is more efficient than red light in inducing stomatal opening. | [6, 39, S80] | Blue light: BL=1, RL=0, ABA=0, CO_2_=C_i_=1 | Stomatal opening=4.15 | C |
|  |  | Red light: BL=0, RL=1, ABA=0, CO_2_=C_i_=1 | Stomatal opening=1 |  |
| Red background illumination synergistically increases the stomatal response to low intensity blue light. | [5, 9, 58] | Monochromatic red light: BL=0, RL=1, ABA=0, CO_2_=C_i_=1 | Stomatal opening=1 | C |
|  |  | Monochromatic blue light: BL=1, RL=0, ABA=0, CO_2_=C_i_=1 | Stomatal opening=4.15 |  |
|  |  | Blue light with red light background: BL=1, RL=1, ABA=0, CO_2_=C_i_=1 | Stomatal opening=11.28 |  |
| phot1 single knockout mutation does not inhibit blue light-induced stomatal opening. | [16] | Wild type under blue light: BL=1, RL=0, ABA=0, CO_2_=C_i_=1 | Stomatal opening=4.15 | C |
|  |  | phot1 knockout under blue light: BL=1, RL=0, ABA=0, CO_2_=C_i_=1, phot1 is kept 0 | Stomatal opening=4.15 |  |
| phot2 single knockout mutation does not inhibit blue light-induced stomatal opening. | [16] | Wild type under blue light: BL=1, RL=0, ABA=0, CO_2_=C_i_=1 | Stomatal opening=4.15 | C |
|  |  | phot2 knockout under blue light: BL=1, RL=0, ABA=0, CO_2_=C_i_=1, phot2 is kept 0 | Stomatal opening=4.15 |  |
| phot1 and phot2 double knockout mutation inhibits blue light-induced stomatal opening. | [16] | Wild type under blue light: BL=1, RL=0, ABA=0, CO_2_=Ci=1 | Stomatal opening=4.15 | C |
|  |  | phot1 and phot2 double knockout under blue light: BL=1, RL=0, ABA=0, CO_2_=C_i_=1, both phot1 and phot2 are kept 0 | Stomatal opening=1 |  |
| phot1 and phot2 double knockout mutation does not inhibit red light-induced stomatal opening. | [16, S61] | Wild type under red light: BL=0, RL=1, ABA=0, CO_2_=C_i_=1 | Stomatal opening=1 | C |
|  |  | phot1 and phot2 double knockout under red light: BL=0, RL=1, ABA=0, CO_2_=C_i_=1, both phot1 and phot2 are kept 0 | Stomatal opening=1 |  |
| phot1 and phot2 double knockout mutation inhibits white light-induced stomatal opening. | [16, S61] | Wild type under white light: BL=1, RL=1, ABA=0, CO_2_=C_i_=1 | Stomatal opening=11.28 | C |
|  |  | phot1 and phot2 double knockout under white light: BL=1, RL=1, ABA=0, CO_2_=C_i_=1, both phot1 and phot2 are kept 0 | Stomatal opening=4.36 |  |
| Cytosolic Ca^2+^ oscillates in response to blue light. | [52] | BL=1, RL=0, ABA=0, CO_2_=C_i_=1 | [Ca^2+^]_c_ oscillates between 0 and 1 | C |
| phot1 and phot2 double knockout reduces cytosolic Ca^2+^ response to blue light. | [52] | Wild type under blue light: BL=1, RL=0, ABA=0, CO_2_=C_i_=1 | [Ca^2+^]_c_ oscillates between 0 and 1 | C |
|  |  | phot1 and phot2 double knockout under blue light: BL=1, RL=0, ABA=0, CO_2_=C_i_=1, both phot1 and phot2 are kept 0 | [Ca^2+^]_c_=0 |  |
| Cytosolic Ca^2+^ does not respond to red light. | [52] | BL=0, RL=1, ABA=0, CO_2_=C_i_=1 | [Ca^2+^]_c_=0 | C |
| Protein phosphatase inhibitors inhibit blue light-induced stomatal opening. | [S13] | Without protein phosphatase inhibitor under blue light: BL=1, RL=0, ABA=0, CO_2_=C_i_=1 | Stomatal opening=4.15 | C |
|  |  | With protein phosphatase inhibitor under blue light: BL=1, RL=0, ABA=0, CO_2_=C_i_=1, PP1_cc_ is kept 0 | Stomatal opening=1 |  |
| The protein phosphatase 1 inhibitor tautomycin inhibits white light-induced opening. | [S14, S15] | Without protein phosphatase inhibitor under white light: BL=1, RL=1, ABA=0, CO_2_=C_i_=1 | Stomatal opening=11.28 | C |
|  |  | With protein phosphatase inhibitor under white light: BL=1, RL=1, ABA=0, CO_2_=C_i_=1, PP1_cc_ is kept 0 | Stomatal opening=2 |  |
| The protein phosphatase 1 inhibitor tautomycin does not inhibit red light-induced opening. | [S14, S15] | Without protein phosphatase inhibitor under red light: BL=0, RL=1, ABA=0, CO_2_=C_i_=1 | Stomatal opening=1 | C |
|  |  | With protein phosphatase inhibitor under red light: BL=0, RL=1, ABA=0, CO_2_=C_i_=1, PP1_cc_ is kept 0 | Stomatal opening=1 |  |
| PRSL1 knockout mutation inhibits dual beam-induced stomatal opening. | [S15] | Wild type under dual beam: BL=1, RL=1, ABA=0, CO_2_=C_i_=1 | Stomatal opening=11.28 | C |
|  |  | PRSL1 knockout under dual beam: BL=1, RL=1, ABA=0, CO_2_=C_i_=1, PRSL1 is kept 0 | Stomatal opening=4.36 |  |
| PRSL1 knockout does not inhibit red light-induced stomatal opening. | [S15] | Wild type under red light: BL=0, RL=1, ABA=0, CO_2_=C_i_=1 | Stomatal opening=1 | C |
|  |  | PRSL1 knockout under red light: BL=0, RL=1, ABA=0, CO_2_=C_i_=1, PRSL1 is kept 0 | Stomatal opening=1 |  |
| Blue light activates the H^+^-ATPase. | [18, 64] | BL=1, RL=0, ABA=0, CO_2_=C_i_=1 | H^+^-ATPase_complex_=2 | C |
| phot1 and phot2 double knockout mutation inhibits blue light-activated H^+^-ATPase activity. | [18] | Wild type under blue light: BL=1, RL=0, ABA=0, CO_2_=C_i_=1 | H^+^-ATPase_complex_=2 | C |
|  |  | phot1 and phot2 double knockout under blue light: BL=1, RL=0, ABA=0, CO_2_=C_i_=1, both phot1 and phot2 are kept 0 | H^+^-ATPase_complex_=0 |  |
| Red light does not activate the H^+^-ATPase. | [18, 64] | BL=0, RL=1, ABA=0, CO_2_=C_i_=1 | H^+^-ATPase_complex_=0 | C |
| Red light does not enhance blue light-dependent H^+^-ATPase activity under a condition of excess ATP and fixed C_i_. | [64] | Blue light with excess ATP and fixed C_i_: BL=1, RL=0, ABA=0, ATP=3, CO_2_ and C_i_ are fixed at a certain value, e.g. CO_2_=C_i_=1 | H^+^-ATPase_complex_=3 | C |
|  |  | Blue light and red light with excess ATP and fixed C_i_: BL=1, RL=1, ABA=0, ATP=3, CO_2_ and C_i_ are fixed at the same value as they are in the previous condition | H^+^-ATPase_complex_=3 |  |
| Inhibiting the H^+^-ATPase with vanadate inhibits white light-induced stomatal opening. | [71, 72] | Without vanadate under white light: BL=1, RL=1, ABA=0, CO_2_=C_i_=1 | Stomatal opening=11.28 | C |
|  |  | With vanadate under white light: BL=1, RL=1, ABA=0, CO_2_=C_i_=1, H^+^-ATPase_complex_ is kept 0 | Stomatal opening=2 |  |
| Fusicoccin stimulates stomatal opening. | [67] | Dark without fusicoccin: BL=0, RL=0, ABA=0, CO_2_=C_i_=1 | Stomatal opening=0 | C |
|  |  | Dark with fusicoccin: BL=0, RL=0, ABA=0, CO_2_=C_i_=1, H^+^-ATPase_complex_ is kept 9 | Stomatal opening=14.18 |  |
| Fusicoccin stimulates guard cell K^+^ uptake. | [67] | Dark without fusicoccin: BL=0, RL=0, ABA=0, CO_2_=C_i_=1 | [K^+^]_c_=0 | C |
|  |  | Dark with fusicoccin: BL=0, RL=0, ABA=0, CO_2_=C_i_=1, H^+^-ATPase_complex_ is kept 9 | [K^+^]_c_=9 |  |
| CO_2_-free air promotes white light-induced stomatal opening. | [1] | White light in ambient air: BL=1, RL=1, ABA=0, CO_2_=C_i_=1 | Stomatal opening=11.28 | C |
|  |  | White light in CO_2_-free air: BL=1, RL=1, ABA=0, CO_2_=C_i_=0 | Stomatal opening=14.01 |  |
| Reduced CO_2_ concentration enhances red light-induced stomatal opening. | [40] | Red light in ambient air: BL=0, RL=1, ABA=0, CO_2_=C_i_=1 | Stomatal opening=1 | C |
|  |  | Red light in reduced CO_2_ air: BL=0, RL=1, ABA=0, CO_2_=C_i_=0 | Stomatal opening=3.15 |  |
| Reduced CO_2_ concentration enhances blue light-induced stomatal opening. | [5] | Blue light in ambient air: BL=1, RL=0, ABA=0, CO_2_=C_i_=1 | Stomatal opening=4.15 | C |
|  |  | Blue light in reduced CO_2_ air: BL=1, RL=0, ABA=0, CO_2_=C_i_=0 | Stomatal opening=9.28 |  |
| The plasma membrane hyperpolarizes under red light in CO_2_-free air. | [S55] | BL=0, RL=1, ABA=0, CO_2_=C_i_=0 | PMV=-1 | C |
| High CO_2_ inhibits stomatal opening. | [S42] | Moderate CO_2_: BL=1, RL=1, ABA=0, CO_2_=C_i_=1 | Stomatal opening=11.28 | C |
|  |  | High CO_2_: BL=1, RL=1, ABA=0, CO_2_=C_i_=2 | Stomatal opening=2 |  |
| High C_i_ depolarizes the plasma membrane. | [86] | Moderate CO_2_: BL=1, RL=1, ABA=0, CO_2_=C_i_=1 | PMV=-2 | C |
|  |  | High CO_2_: BL=1, RL=1, ABA=0, CO_2_=C_i_=2 | PMV=0 |  |
| The guard cell plasma membrane depolarizes under red light in CO_2_-containing air. | [S55] | BL=0, RL=1, ABA=0, CO_2_=C_i_=1 | PMV=0 | C |
| The plasma membrane hyperpolarizes in response to light, and depolarizes in the dark. | [79] | Light: BL=1, RL=1, ABA=0, CO_2_=C_i_=1 | PMV=-2 | C |
|  |  | Dark: BL=0, RL=0, ABA=0, CO_2_=C_i_=1 | PMV=0 |  |
| Under equal quantum flux, blue light is more efficient than red light in inducing Rb^+^ (a K^+^ equivalent) uptake. | [6, 39] | Blue light: BL=1, RL=0, ABA=0, CO_2_=C_i_=1 | [K^+^]_c_=2 | C |
|  |  | Red light: BL=0, RL=1, ABA=0, CO_2_=C_i_=1 | [K^+^]_c_=0 |  |
| White light-induced stomatal opening is inhibited by K_in_ channel knockout mutation. | [73] | Wild type under white light: BL=1, RL=1, ABA=0, CO_2_=C_i_=1 | Stomatal opening=11.28 | C |
|  |  | K_in_ channel knockout under white light: BL=1, RL=1, ABA=0, CO_2_=C_i_=1, K_in_ is kept 0 | Stomatal opening=2 |  |
| Blue light-induced stomatal opening is inhibited by K_in_ channel knockout mutation. | [73] | Wild type under blue light: BL=1, RL=0, ABA=0, CO_2_=C_i_=1 | Stomatal opening=4.15 | C |
|  |  | K_in_ channel knockout under blue light: BL=1, RL=0, ABA=0, CO_2_=C_i_=1, K_in_ is kept 0 | Stomatal opening=1 |  |
| Red light-induced stomatal opening is not inhibited by K_in_ channel knockout mutation. | [73] | Wild type under red light: BL=0, RL=1, ABA=0, CO_2_=C_i_=1 | Stomatal opening=1 | C |
|  |  | K_in_ channel knockout under red light: BL=0, RL=1, ABA=0, CO_2_=C_i_=1, K_in_ is kept 0 | Stomatal opening=1 |  |
| Nitrate transporter CHL1 knockout inhibits white light-induced stomatal opening. | [S48] | Wild type under white light: BL=1, RL=1, ABA=0, CO_2_=C_i_=1 | Stomatal opening=11.28 | C |
|  |  | CHL1 knockout under white light: BL=1, RL=1, ABA=0, CO_2_=C_i_=1, CHL1 is kept 0 | Stomatal opening=10.68 |  |
| The rate of malate formation under blue light with a red light background is larger than the sum of rates under monochromatic blue or red light. | [13] | Monochromatic red light: BL=0, RL=1, ABA=0, CO_2_=C_i_=1 | [malate^2-^]_c_=0 | C |
|  |  | Monochromatic blue light: BL=1, RL=0, ABA=0, CO_2_=C_i_=1 | [malate^2-^]_c_=1.5 |  |
|  |  | Blue light with a red light background: BL=1, RL=1, ABA=0, CO_2_=C_i_=1 | [malate^2-^]_c_=6.5 |  |
| Malate transporter AtABCB14 knockout mutant displays reduced white light-induced stomatal opening. | [S58] | Wild type under white light: BL=1, RL=1, ABA=0, CO_2_=C_i_=1 | Stomatal opening=11.28 | IC |
|  |  | AtABCB14 knockout under white light: BL=1, RL=1, ABA=0, CO_2_=C_i_=1, AtABCB14 is kept 0 | Stomatal opening=11.28 |  |
| Sucrose concentration increases during white light-induced stomatal opening. | [34] | Dark: BL=0, RL=0, ABA=0, CO_2_=C_i_=1 | Sucrose=0 | C |
|  |  | White light: BL=1, RL=1, ABA=0, CO_2_=C_i_=1 | Sucrose=2 |  |
| Sucrose concentration increases during blue light-induced stomatal opening. | [35] | Dark: BL=0, RL=0, ABA=0, CO_2_=C_i_=1 | Sucrose=0 | C |
|  |  | Blue light: BL=1, RL=0, ABA=0, CO_2_=C_i_=1 | Sucrose=1 |  |
| Sucrose concentration increases during red light-induced stomatal opening. | [35] | Dark: BL=0, RL=0, ABA=0, CO_2_=C_i_=1 | Sucrose=0 | C |
|  |  | Red light: BL=0, RL=1, ABA=0, CO_2_=C_i_=1 | Sucrose=1 |  |
| PLA_2_β knockout mutant exhibits reduced white light-induced stomatal opening compared to wild type. | [S2] | Wild type under white light: BL=1, RL=1, ABA=0, CO_2_=C_i_=1 | Stomatal opening=11.28 | C |
|  |  | PLA_2_β knockout under white light: BL=1, RL=1, ABA=0, CO_2_=C_i_=1, PLA_2_β is kept 0 | Stomatal opening=2 |  |
| PIP_2_ knockout mutant displays reduced white light-induced stomatal opening compared to wild type. | [S16] | Wild type under white light: BL=1, RL=1, ABA=0, CO_2_=C_i_=1 | Stomatal opening=11.28 | IC |
|  |  | PIP_2_ knockout under white light: BL=1, RL=1, ABA=0, CO_2_=C_i_=1, PIP2_PM_ is kept 0 | Stomatal opening=11.28 |  |
| Dominant negative mutant of the small G protein ROP2 exhibits enhanced stomatal opening in response to white light. | [74] | Wild type under white light: BL=1, RL=1, ABA=0, CO_2_=C_i_=1 | Stomatal opening=11.28 | C |
|  |  | Dominant negative small G protein ROP2 mutant under white light: BL=1, RL=1, ABA=0, CO_2_=C_i_=1, ROP2 is kept 0 | Stomatal opening=11.45 |  |
| ABA inhibits white light-induced stomatal opening. | [2, 18, 41, 43, S78] | White light without ABA: BL=1, RL=1, ABA=0, CO_2_=C_i_=1 | Stomatal opening=11.28 | C |
|  |  | White light with ABA: BL=1, RL=1, ABA=1, CO_2_=C_i_=1 | Stomatal opening=2 |  |
| ABA inhibits blue light-induced stomatal opening. | [17] | Blue light without ABA: BL=1, RL=0, ABA=0, CO_2_=C_i_=1 | Stomatal opening=4.15 | C |
|  |  | Blue light with ABA: BL=1, RL=0, ABA=1, CO_2_=C_i_=1 | Stomatal opening=1 |  |
| ABA induces cytosolic Ca^2+^ oscillation. | [43] | BL=1, RL=1, ABA=1, CO_2_=C_i_=1 | In the model cytosolic Ca^2+^ increases, peaks, then it decreases in response to ABA; there is no consecutive increase. | PC |
| ROS inhibits white light-induced stomatal opening. | [S3] | White light without ROS: BL=1, RL=1, ABA=0, CO_2_=C_i_=1 | Stomatal opening=11.28 | C |
|  |  | White light with ROS: BL=1, RL=1, ABA=0, CO_2_=C_i_=1, ROS is kept 1 | Stomatal opening=8.92 |  |
| ROS inhibits blue light-induced stomatal opening. | [17] | Blue light without ROS: BL=1, RL=0, ABA=0, CO_2_=C_i_=1 | Stomatal opening=4.15 | C |
|  |  | Blue light with ROS: BL=1, RL=0, ABA=0, CO_2_=C_i_=1, ROS is kept 1 | Stomatal opening=3.84 |  |
| NO donor SNP inhibits white light-induced stomatal opening. | [S3] | White light without NO donor SNP: BL=1, RL=1, ABA=0, CO_2_=C_i_=1 | Stomatal opening=11.28 | C |
|  |  | White light with NO donor SNP: BL=1, RL=1, ABA=0, CO_2_=C_i_=1, NO is kept 1 | Stomatal opening=8.92 |  |
| NO donor SNP inhibits blue light-induced stomatal opening. | [17, S74] | Blue light without NO donor SNP: BL=1, RL=0, ABA=0, CO_2_=C_i_=1 | Stomatal opening=4.15 | C |
|  |  | Blue light with NO donor SNP: BL=1, RL=0, ABA=0, CO_2_=C_i_=1, NO is kept 1 | Stomatal opening=3.84 |  |
| NO donor SNP does not inhibit red light-induced stomatal opening. | [S74] | Red light without NO donor SNP: BL=0, RL=1, ABA=0, CO_2_=C_i_=1 | Stomatal opening=1 | C |
|  |  | Red light with NO donor SNP: BL=0, RL=1, ABA=0, CO_2_=C_i_=1, NO is kept 1 | Stomatal opening=1 |  |
| NO scavenger PTIO partially restores stomatal opening inhibited by ABA. | [S6] | White light without ABA: BL=1, RL=1, ABA=0, CO_2_=C_i_=1 | Stomatal opening=11.28 | C |
|  |  | White light with ABA: BL=1, RL=1, ABA=1, CO_2_=C_i_=1 | Stomatal opening=2 |  |
|  |  | White light with ABA and NO scavenger PTIO: BL=1, RL=1, ABA=1, CO_2_=C_i_=1, NO is kept 0 | Stomatal opening=7.01 |  |
| Anion channel blocker 9-AC reverses inhibition of white light-induced stomatal opening by ABA. | [41] | White light without ABA: BL=1, RL=1, ABA=0, CO_2_=C_i_=1 | Stomatal opening=11.28 | C |
|  |  | White light with ABA: BL=1, RL=1, ABA=1, CO_2_=C_i_=1 | Stomatal opening=2 |  |
|  |  | White light with ABA and anion channel blocked by 9-AC: BL=1, RL=1, ABA=1, CO_2_=C_i_=1, AnionCh is kept 0 | Stomatal opening=3.73 |  |
| ABA can activate anion efflux channels without the mediation of Ca^2+^. | [S73] | With the mediation of Ca^2+^: BL=1, RL=1, ABA=1, CO_2_=C_i_=1 | AnionCh=1.6 | C |
|  |  | Without the mediation of Ca^2+^: BL=1, RL=1, ABA=1, CO_2_=C_i_=1, [Ca^2+^]_c_ is kept 0 | AnionCh=1.6 |  |
| ABA inhibits blue light-induced H^+^-ATPase activity. | [44] | Without ABA: BL=1, RL=0, ABA=0, CO_2_=C_i_=1 | H^+^-ATPase_complex_=2 | C |
|  |  | With ABA: BL=1, RL=0, ABA=1, CO_2_=C_i_=1 | H^+^-ATPase_complex_=1 |  |
| ROS inhibits blue light-induced H^+^-ATPase activity. | [44] | Without ROS: BL=1, RL=0, ABA=0, CO_2_=C_i_=1 | H^+^-ATPase_complex_=2 | C |
|  |  | With ROS: BL=1, RL=0, ABA=0, CO_2_=C_i_=1, ROS is kept 1 | H^+^-ATPase_complex_=1.8 |  |
| ROS scavenger partially restores blue light-dependent H^+^-ATPase activity inhibited by ABA. | [44] | Blue light without ABA: BL=1, RL=0, ABA=0, CO_2_=C_i_=1 | H^+^-ATPase_complex_=2 | C |
|  |  | Blue light with ABA: BL=1, RL=0, ABA=1, CO_2_=C_i_=1 | H^+^-ATPase_complex_=1 |  |
|  |  | Blue light with ABA and ROS scavenger: BL=1, RL=0, ABA=1, CO_2_=C_i_=1, ROS is kept 0 | H^+^-ATPase_complex_=1.8 |  |
| PA inhibits white light-induced stomatal opening. | [42, S78] | White light: BL=1, RL=1, ABA=0, CO_2_=C_i_=1 | Stomatal opening=11.28 | C |
|  |  | White light with sustained PA: BL=1, RL=1, ABA=0, CO_2_=C_i_=1, PA is kept 1 | Stomatal opening=8.92 |  |
| PA inhibits blue light-induced stomatal opening. | [17] | Blue light: BL=1, RL=0, ABA=0, CO_2_=C_i_=1 | Stomatal opening=4.15 | C |
|  |  | Blue light with sustained PA: BL=1, RL=0, ABA=0, CO_2_=C_i_=1, PA is kept 1 | Stomatal opening=3.84 |  |
| PA does not inhibit red light-induced stomatal opening. | [17] | Red light: BL=0, RL=1, ABA=0, CO_2_=C_i_=1 | Stomatal opening=1 | C |
|  |  | Red light with sustained PA: BL=0, RL=1, ABA=0, CO_2_=C_i_=1, PA is kept 1 | Stomatal opening=1 |  |
| The inhibition (with 1-buOH) of PA production elicited by ABA partially prevents ABA's inhibition of white light-induced stomatal opening. | [42] | White light without ABA: BL=1, RL=1, ABA=0, CO_2_=C_i_=1 | Stomatal opening=11.28 | C |
|  |  | White light with ABA: BL=1, RL=1, ABA=1, CO_2_=C_i_=1 | Stomatal opening=2 |  |
|  |  | White light with ABA and PA inhibitor 1-buOH: BL=1, RL=1, ABA=1, CO_2_=C_i_=1, PA is kept 0 | Stomatal opening=8.73 |  |
| OST1 knockout mutation does not affect light-induced stomatal opening. | [18, S30] | Wild type under light: BL=1, RL=1, ABA=0, CO_2_=C_i_=1 | Stomatal opening=11.28 | C |
|  |  | OST1 knockout mutant under light: BL=1, RL=1, ABA=0, CO_2_=C_i_=1, OST1 is kept 0 | Stomatal opening=11.28 |  |
| OST1 knockout mutation disrupts ABA's inhibition of white light-induced stomatal opening. | [18, S30] | Wild type under white light without ABA: BL=1, RL=1, ABA=0, CO_2_=C_i_=1 | Stomatal opening=11.28 | C |
|  |  | Wild type under white light with ABA: BL=1, RL=1, ABA=1, CO_2_=C_i_=1 | Stomatal opening=2 |  |
|  |  | OST1 knockout mutant under white light with ABA: BL=1, RL=1, ABA=1, CO_2_=C_i_=1, OST1 is kept 0 | Stomatal opening=7.01 |  |
| ABA upregulates NADPH oxidases AtrbohD/F. | [S75] | Without ABA: BL=1, RL=1, ABA=0, CO_2_=C_i_=1 | AtrbohD/F=0 | C |
|  |  | With ABA: BL=1, RL=1, ABA=1, CO_2_=C_i_=1 | AtrbohD/F=1 |  |
| AtrbohD/F double knockout mutation impairs ROS production in response to ABA compared to wild type. | [S75] | Wild type with ABA: BL=1, RL=1, ABA=1, CO_2_=C_i_=1 | ROS=1 | C |
|  |  | AtrbohD/F double knockout mutant with ABA: BL=1, RL=1, ABA=1, CO_2_=C_i_=1, AtrbohD/F is kept 0 | ROS=0 |  |
| Inhibiting NADPH oxidase with DPI partially restores stomatal opening inhibited by ABA. | [76] | White light without ABA: BL=1, RL=1, ABA=0, CO_2_=C_i_=1; | Stomatal opening=11.28 | C |
|  |  | White light with ABA: BL=1, RL=1, ABA=1, CO_2_=C_i_=1 | Stomatal opening=2 |  |
|  |  | White light with ABA and NADPH oxidase inhibitor DPI: BL=1, RL=1, ABA=1, CO_2_=C_i_=1, AtrbohD/F is kept 0 | Stomatal opening=7.01 |  |
